# Epithelial de-differentiation triggered by co-ordinate epigenetic inactivation of the *EHF* and *CDX1* transcription factors drives colorectal cancer progression

**DOI:** 10.1038/s41418-022-01016-w

**Published:** 2022-05-23

**Authors:** Ian Y. Luk, Laura J. Jenkins, Kael L. Schoffer, Irvin Ng, Janson W. T. Tse, Dmitri Mouradov, Stanislaw Kaczmarczyk, Rebecca Nightingale, Allan D. Burrows, Robin L. Anderson, Diego Arango, Higinio Dopeso, Larry Croft, Mark F. Richardson, Oliver M. Sieber, Yang Liao, Jennifer K. Mooi, Natalia Vukelic, Camilla M. Reehorst, Shoukat Afshar-Sterle, Vicki L. J. Whitehall, Lochlan Fennell, Helen E. Abud, Niall C. Tebbutt, Wayne A. Phillips, David S. Williams, Wei Shi, Lisa A. Mielke, Matthias Ernst, Amardeep S. Dhillon, Nicholas J. Clemons, John M. Mariadason

**Affiliations:** 1 Olivia Newton-John Cancer Research Institute, Melbourne, VIC Australia; 2 La Trobe University School of Cancer Medicine, Melbourne, VIC Australia; 3 Department of Medicine, University of Melbourne, Melbourne, VIC Australia; 4 Personalised Oncology Division, The Walter and Eliza Hall Institute of Medial Research, Parkville, VIC Australia; 5 Department of Medical Biology, The University of Melbourne, Parkville, VIC Australia; 6 The Sir Peter MacCallum Department of Oncology, University of Melbourne, Melbourne, VIC Australia; 7 Group of Biomedical Research in Digestive Tract Tumors, CIBBIM-Nanomedicine, Vall d’Hebron University Hospital Research Institute (VHIR), Barcelona, Spain; 8 Group of Molecular Oncology, Biomedical Research institute of Lleida (IRBLleida), Lleida, Spain; 9 School of Life and Environmental Sciences, Deakin University, Geelong, VIC Australia; 10 Department of Surgery, The University of Melbourne, Parkville, VIC Australia; 11 Department of Biochemistry and Molecular Biology, Monash University, Clayton, VIC Australia; 12 QIMR Berghofer Medical Research Institute, Herston, QLD Australia; 13 Conjoint Internal Medicine Laboratory, Pathology Queensland, Brisbane, QLD Australia; 14 The University of Queensland, St Lucia, QLD Australia; 15 Development and Stem Cells Program and the Department of Anatomy and Developmental Biology, Monash Biomedicine Discovery Institute, Monash University, Melbourne, VIC Australia; 16 Peter MacCallum Cancer Centre, Melbourne, VIC Australia; 17 Department of Anatomical Pathology, Austin Health, Heidelberg, VIC Australia; 18 Department of Clinical Pathology, University of Melbourne, Parkville, VIC Australia; 19 School of Computing and Information Systems, University of Melbourne, Parkville, VIC Australia; 20 The Institute for Mental and Physical Health and Clinical Translation, School of Medicine, Deakin University, Geelong, VIC Australia

**Keywords:** Tumour-suppressor proteins, Epigenetics

## Abstract

Colorectal cancers (CRCs) often display histological features indicative of aberrant differentiation but the molecular underpinnings of this trait and whether it directly drives disease progression is unclear. Here, we identify co-ordinate epigenetic inactivation of two epithelial-specific transcription factors, EHF and CDX1, as a mechanism driving differentiation loss in CRCs. Re-expression of EHF and CDX1 in poorly-differentiated CRC cells induced extensive chromatin remodelling, transcriptional re-programming, and differentiation along the enterocytic lineage, leading to reduced growth and metastasis. Strikingly, EHF and CDX1 were also able to reprogramme non-colonic epithelial cells to express colonic differentiation markers. By contrast, inactivation of EHF and CDX1 in well-differentiated CRC cells triggered tumour de-differentiation. Mechanistically, we demonstrate that EHF physically interacts with CDX1 via its PNT domain, and that these transcription factors co-operatively drive transcription of the colonic differentiation marker, *VIL1*. Compound genetic deletion of *Ehf* and *Cdx1* in the mouse colon disrupted normal colonic differentiation and significantly enhanced colorectal tumour progression. These findings thus reveal a novel mechanism driving epithelial de-differentiation and tumour progression in CRC.

## Introduction

Loss of differentiation (de-differentiation) is a histopathological feature of CRC defined by the extent to which a tumour loses the glandular morphology of the normal colonic epithelium. Approximately 80% of CRCs are classified as low grade (well- to moderately-differentiated) and retain a high percentage of glandular structure, while the remainder are classified as high grade (poorly or undifferentiated), and display more solid growth patterns with partial or complete loss of glandular structures [1]. These histological changes are also associated with loss of expression of classical colonic differentiation markers such as villin (VIL1) and keratin 20 (KRT20) [2, 3].

Poorly-differentiated histology is associated with specific molecular features, including microsatellite instability (MSI) [4], the CpG-island methylator phenotype (CIMP) [4, 5] and *BRAF* mutation status [6, 7], and phenotypically, with increased metastatic propensity, reduced response to chemoradiation therapy, and poorer outcome [8, 9]. Re-induction of differentiation therefore represents a significant opportunity to develop new treatment paradigms aimed at reducing tumour progression and metastatic spread and enhancing chemosensitivity. Importantly, while the clinical utility of differentiation therapy is well established by the use of all-*trans* retinoic acid for treating acute promyelocytic leukaemia, which induces the differentiation of APMC cells into granulocytes [10], its potential for the treatment of colorectal cancer (CRC) has not been investigated. Understanding the transcriptional and epigenetic events which underpin differentiation loss is fundamental to this endeavour.

Ets homologous factor (EHF) is a member of the epithelial-specific ETS (ESE) transcription factors comprising ESE-1 (ELF3), ESE-2 (ELF5), ESE-3 (EHF) and SPDEF [11], which regulate multiple processes, including cell proliferation and differentiation [12]. Studies in cell lines have demonstrated that EHF is important for maintaining epithelial identity in prostate [13] and pancreatic cancers [14], and maintenance of intestinal stem cells [15]. Using a novel mouse model in which the *Ehf* was deleted in all tissues or conditionally in the colonic epithelium [16], we recently identified a key role for EHF in maintaining epidermal and intestinal epithelial homoeostasis. However, the significance of EHF deficiency in the pathobiology of human CRCs remains unknown.

Here, we reveal that EHF expression is lost in a subset of poorly-differentiated CRCs, along with several other transcription factors known to be required for colonic differentiation. While re-expression of EHF alone failed to re-induce differentiation of these tumours, combined re-expression of EHF and CDX1 re-induced expression of multiple enterocytic differentiation markers and gland formation, and inhibited tumour growth and metastasis. Conversely, compound deletion of *Ehf* and *Cdx1* in mice reduced expression of colonic differentiation markers and significantly accelerated colorectal tumour progression. Remarkably, re-expression of EHF and CDX1 in non-colonic cells was also sufficient to induce expression of colonic differentiation markers. Finally, we demonstrate that EHF physically interacts with CDX1 via its PNT domain, and that these factors co-operatively drive transcription of the colonic differentiation marker, VIL1. These findings identify a novel mechanism of CRC progression driven by epigenetic inactivation of the key colonic lineage-regulating transcription factors EHF and CDX1.

## Results

### EHF expression is lost in a subset of poorly-differentiated CRCs

**Fig. 1:**
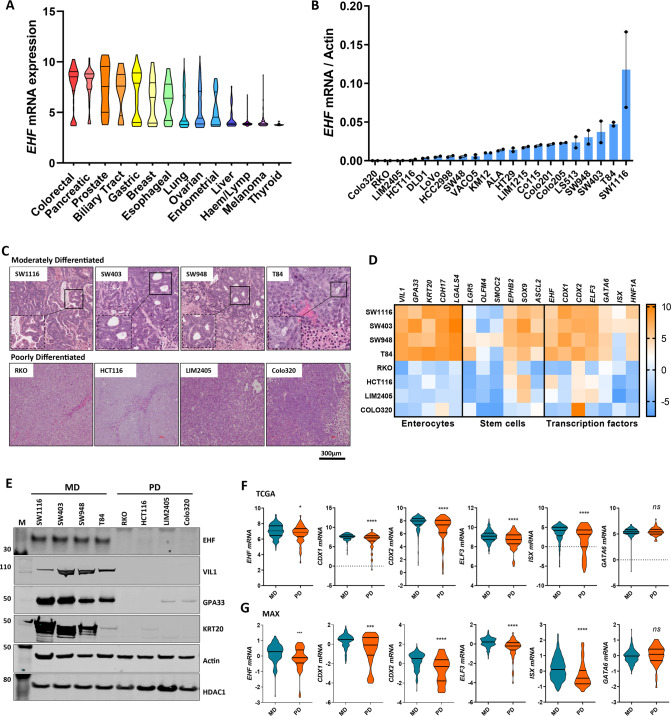
*EHF* expression is downregulated in poorly-differentiated colorectal cancers. **A** *EHF* mRNA expression in 1080 cancer cell lines derived from various tumour types obtained from the Broad Institute Cancer Cell Line Encyclopaedia (CCLE) database. **B** Validation of *EHF* mRNA expression in 20 CRC cell lines by q-RT-PCR. Data shown are mean ± SEM from a single experiment performed in duplicate. **C** Haematoxylin and Eosin (H&E) staining of 4 moderately-differentiated (MD) and four poorly-differentiated (PD) CRC cell lines grown as xenografts showing greater presence of glandular structures in MD lines. **D** mRNA expression of markers of enterocytes, colonic stem cells and colonic lineage determining transcription factors in moderately and poorly-differentiated CRC cell lines. Data shown are the normalised mRNA expression levels from a single RNA analysis per cell line. **E** Western blots of EHF, VIL1, GPA33 and KRT20 protein in 4 moderately-differentiated and 4 poorly-differentiated CRC cell lines. EHF expression was determined in nuclear lysates, and HDAC-1 expression was assessed as a loading control. Actin was used as a loading control for VIL1, GPA33 and KRT20. **F**, **G** Violin plots of the mRNA expression levels of transcription factors implicated in colonic lineage determination in *n* = 181 moderately-differentiated (MD) and *n* = 52 poorly-differentiated (PD) primary CRCs. Data were derived from (**F**) the COAD cohort profiled by the TCGA (The Cancer Genome Atlas) or **G** the phase III MAX clinical trial cohort [18]. *p < 0.05; **p < 0.01; ***p < 0.001; ****p < 0.0001, Student’s *t* test.

Through interrogation of the Broad Institute CCLE (Cancer Cell Line Encyclopaedia) database consisting of >1000 cell lines [17], we found that while many CRC cell lines expressed high levels of EHF transcript, a subset of lines had low or no *EHF* expression (RKO, HCT116, LIM2405 and Colo320) (Fig. 1A, B). Histopathological analysis of xenografts grown from these cell lines revealed that they all formed poorly-differentiated tumours, lacking glandular structures. By contrast, EHF-expressing CRC cell lines (SW116, SW403, SW948 and T84) formed well-to-moderately-differentiated tumours (Fig. 1C). We also noted that EHF^low^ CRC cells expressed low levels of colonic differentiation markers (VIL1, GPA33, KRT20), and several other transcription factors (CDX1, ELF3, GATA6, ISX) known to be enriched in the colonic epithelium (Fig. 1D, E). Strikingly, markers of normal colon stem cells (LGR5, ASCL2, SOX9), were also strongly downregulated EHF^low^ CRC cells, suggesting that these lines give rise to tumours that are de-differentiated to the point of losing their colonic identity.

To assess the relevance of these findings in primary CRCs, we examined *EHF* levels in well and poorly-differentiated CRCs profiled by the TCGA consortium, and in an independent gene expression dataset of 233 CRCs profiled in-house [18]. Consistent with our cell line data, expression of *EHF*, as well as colonic differentiation markers, colonic stem cell markers and colon-specific transcription factors were significantly reduced in poorly-differentiated tumours (Fig. 1F, G,  S1A, B).

### EHF cooperates with CDX1 to promote CRC differentiation

To directly test if *EHF* regulates CRC differentiation, we stably re-expressed *EHF* in the poorly-differentiated CRC cell lines HCT116 and RKO (Fig. S2A, B). Despite inducing EHF to similar levels observed endogenously in moderately-differentiated SW948 cells, there was only a marginal increase in expression of differentiation markers (VIL1, GPA33 and KRT20) (Fig. S2A, B).

**Fig. 2:**
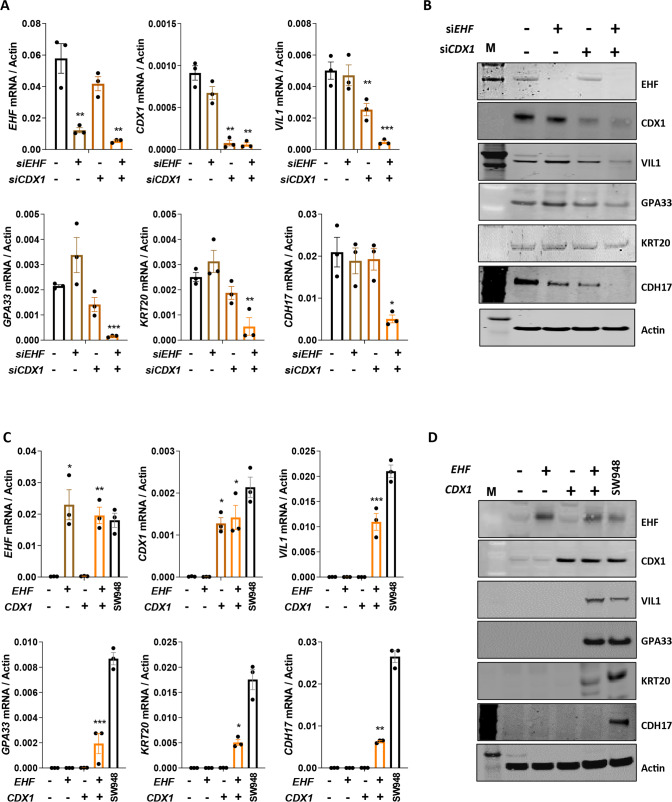
*EHF* and *CDX1* co-operatively regulate differentiation of CRC cells. **A**, **B** Moderately-differentiated SW948 CRC cells were transiently transfected with siRNAs targeting EHF and CDX1 alone and in combination and expression of EHF, CDX1 and differentiation markers was determined by (**A**) q-RT-PCR or (**B**) western blot. **C**, **D** Poorly-differentiated HCT116 CRC cells were stably transfected with *EHF* and *CDX1* alone and in combination, and expression of EHF, CDX1 and differentiation markers determined by (**C**) q-RT-PCR or (**D**) western blot. Values shown in panels A and C are mean ± SEM from a representative experiment performed in triplicate. *p < 0.05; **p < 0.01; ***p < 0.001, one-way ANOVA with Tukey’s post hoc test.

As gene regulation often involves cooperativity between multiple transcription factors, we postulated that the effects of *EHF* loss may only be evident in the context of loss of one or more other transcription factors that we found were also downregulated in poorly-differentiated CRCs (Fig. 1F, G,  S1A, B). To investigate this, we systematically depleted EHF in combination with each of these transcription factors. While simultaneous knockdown of *EHF* with *GATA6*, *ISX* or *ELF3* did not alter expression of differentiation markers (Fig. S3), combined *EHF/CDX1* knockdown markedly repressed expression of multiple differentiation markers in SW948 cells (Fig. 2A, B), findings that were confirmed in a second moderately-differentiated CRC cell line, SW403 (Fig. S4A, B).

To further validate these data, we stably re-expressed EHF and CDX1 alone and in combination in poorly-differentiated HCT116 cells to levels comparable with their endogenous expression in moderately-differentiated SW948 cells (Fig. 2C, D). As with EHF, re-expression of CDX1 alone had minimal effect on CRC cell differentiation. By contrast, stable re-expression of both EHF and CDX1 robustly induced multiple differentiation markers (VIL1, GPA33 and KRT20) to levels seen in moderately-differentiated SW948 cells (Fig. 2C, D). These findings were confirmed in two additional poorly-differentiated CRC cell lines, SW480 and LIM2405 (Fig. S4C). Finally, to determine whether expression of EHF and CDX1 was able to induce expression of colonic differentiation markers in non-colonic cells, we expressed EHF and CDX1 in human embryonic kidney cells (293T) and mouse embryonic fibroblasts (3T3). Strikingly, we observed a robust increase in endogenous expression of the differentiation markers VIL1 and CDH17 in cells transfected with both EHF and CDX1, demonstrating these factors are able to reprogramme non-colonic epithelial cells to express colonic differentiation markers (Fig. S4D, E).

### EHF and CDX1 co-operate to drive extensive transcriptional re-programming and chromatin remodelling in CRC cells

**Fig. 3:**
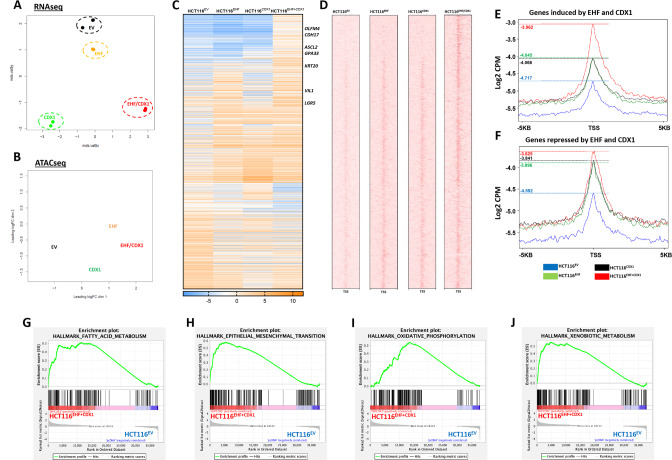
*EHF* and *CDX1* re-expression induces extensive transcriptional re-programming and chromatin remodelling in CRC cells. **A**, **B** Unsupervised cluster analysis of the **A** RNA-seq and **B** ATAC-seq datasets from HCT116^EV^, HCT116^EHF^, HCT116^CDX1^ and HCT116^EHF+CDX1^ cells. Data represents 2 biological replicates in the panel A and a representative experiment in panel B. **C** RNA-seq analysis of the differentially expressed genes (*n* = 1406) both up and downregulated upon EHF and CDX1 re-expression in HCT116 cells compared to empty vector control (HCT116^EV^). **D** Corresponding ATAC-seq analysis (5 kb upstream and downstream of the transcription start site—TSS) of the differentially expressed genes shown in **C**. **E**, **F** Quantitation of the levels of open chromatin of the genes (**E**) upregulated and **F** downregulated in HCT116^EHF+CDX1^ cells compared to control shown in panel C from a representative experiment. **G**–**J** Gene Set Enrichment Analysis (GSEA) of genes differentially expressed between HCT116^EHF + CDX1^ and HCT116^EV^ cells showing enrichment in **G** fatty acid metabolism, **H** epithelial to mesenchymal transition, **I** oxidative phosphorylation and **J** xenobiotic metabolism.

To gain insight into how re-expression of EHF and CDX1 promotes differentiation of CRC cells, we performed parallel RNA-seq and ATAC-seq analysis on HCT116^EV^, HCT116^EHF^, HCT116^CDX1^ and HCT116^EHF + CDX1^ cells to assess global transcriptomic changes and associated chromatin remodelling events. Unsupervised clustering of the two datasets demonstrated distinct clustering of the samples according to *EHF* and *CDX1* expression status (Fig. 3A, B), demonstrating EHF/CDX1 re-expression drives extensive transcriptional re-programming and chromatin remodelling. The RNA-seq analysis identified 862 significantly induced and 543 significantly repressed genes in HCT116^EHF + CDX1^ cells compared to HCT116^EV^ cells, including multiple enterocytic differentiation markers (*VIL1*, *GPA33*, *KRT20* and *CDH17*), and markers of normal colonic stem cells (*OLFM4*, *LGR5* and *ASCL2)* (Fig. 3C). Gene set enrichment analyses (GSEA) demonstrated that re-expression of EHF and CDX1 induced expression of genes involved in a number of biological processes associated with normal colonic differentiation and functioning, including fatty acid metabolism, epithelial-to-mesenchymal transition, oxidative phosphorylation and xenobiotic metabolism (Table S3, Fig. 3G–J).

Examination of the corresponding regulatory regions 5 kb up and downstream of the transcription start site (TSS) of these genes revealed a marked increase in chromatin accessibility centred around the TSS upon EHF/CDX re-expression (Fig. 3D–F).

### EHF and CDX1 co-operatively drive transcription of colonic differentiation markers

**Fig. 4:**
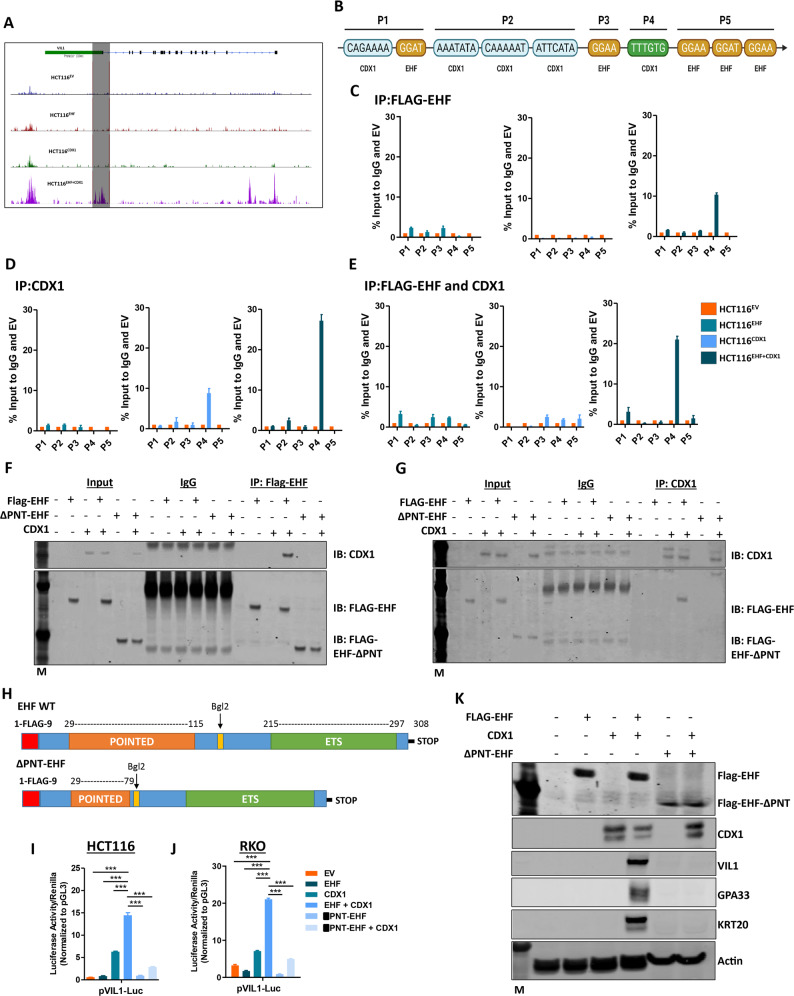
*EHF* and *CDX1* physically interact to regulate activity of differentiation markers in CRC cells. **A** Representative ATAC-Seq tracks of the *VIL1* gene in HCT116^EV^, HCT116^EHF^, HCT116^CDX1^ and HCT116^EHF + CDX1^ cells. **B** Schematic of the structure of the VIL1 promoter. Shown are CDX1 and EHF binding sites. **C**, **D** Binding of **C** FLAG-EHF and **D** CDX1 to different regions of the VIL1 promoter in HCT116^EHF^, HCT116^CDX1^ and HCT116^EHF + CDX1^ cells normalised to IgG control and HCT116^EV^ by ChIP. **E** Re-ChIP analysis assessing binding of FLAG-EHF and CDX1 to various regions of the VIL1 promoter in HCT116^EHF^, HCT116^CDX1^ and HCT116^EHF+CDX1^ cells normalised to IgG control and HCT116^EV^. Values shown are mean ± SEM from a representative experiment in which the q-RT-PCR analysis was performed in triplicate. Similar results were obtained in a separate experiment. **F**, **G** Immunoprecipitation of **F** anti-FLAG-EHF probing for CDX1 and **G** anti-CDX1 probing for FLAG-EHF in HCT116 cells transiently transduced with either EHF, CDX1 or ΔPNT-EHF expression plasmids alone or in combination. **H** Schematic diagram of full-length EHF and ΔPNT-EHF expression plasmids. **I**, **J** Luciferase promoter reporter analysis of VIL1 co-transfected with EHF and CDX1 expression constructs in **I** HCT116 and **J** RKO cells for 72 h. Values shown are mean ± SEM from a representative experiment performed in quadruplicate, with results expressed as fold induction relative to pGL3 and normalised to *Renilla* luciferase activity. Similar results were obtained in a separate experiment. **K** HCT116 cells transiently transduced with EHF, CDX1 or ΔPNT-EHF expression plasmids alone or in combination and expression of EHF, CDX1 and differentiation markers determined by western blot.

To elucidate the mechanism by which *EHF* and *CDX1* co-operate to drive CRC differentiation, we utilised the ATAC-seq data to interrogate how re-expression of these factors alters chromatin accessibility at the *VIL1* promoter. We found that expression of EHF and CDX1 induced open chromatin in a 3 kb region spanning the TSS of the *VIL1* promoter (Fig. 4A). This region contained multiple putative EHF and CDX1 binding sites (Fig. 4B), including a site previously shown to directly bind CDX1 in chromatin immunoprecipitation (ChIP) studies [2]. To determine if EHF and CDX1 directly bind to this region, we performed ChIP analyses on the four isogenic cell lines. While minimal EHF binding was observed in cells re-expressing EHF alone, strong CDX1 binding to region P4 (overlapping the previously reported CDX1 binding site [2]) was observed in cells overexpressing CDX1 alone or in combination with EHF (Fig. 4C). Notably, EHF binding was also enriched at the P4 site in cells expressing both EHF and CDX1, despite the lack of EHF binding sites in this region, suggesting EHF may be recruited to this site by CDX1 (Fig. 4C, D). To directly test this, we performed a re-ChIP experiment in which FLAG-EHF and CDX1 were sequentially immunoprecipitated. This analysis revealed significant enrichment of the P4 region in cells expressing both EHF and CDX1, but not either factor alone (Fig. 4E), demonstrating that binding of EHF at this site requires CDX1.

To test if CDX1 recruits EHF to the *VIL1* promoter to drive transcription, we performed co-immunoprecipitation (IP) experiments in HCT116 cells expressing EHF and CDX1. We found that CDX1 was present in FLAG-EHF immunoprecipitates, while FLAG-EHF was present in CDX1 immunoprecipitates, suggesting that these proteins physically interact in CRC cells (Fig. 4F, G). Given the importance of the PNT domain of Ets transcription factors in mediating protein-protein interactions [19], we next asked if this region is required for EHF interactions with CDX1. We found that an EHF mutant lacking most of the PNT domain (ΔPNT-EHF) failed to immunoprecipitate CDX1, demonstrating that the PNT domain is required for specific binding between these proteins (Fig. 4F–H).

To validate the impact of these findings on gene transcription, we next examined the effects of *EHF*/*CDX1* re-expression on *VIL1* promoter activity, in HCT116 and RKO CRC cells. While *EHF* re-expression alone had minimal effect, CDX1 re-expression significantly increased *VIL1* promoter activity, which was further enhanced in the presence of EHF (Fig. 4I, J). Comparatively, cells transfected with ΔPNT-EHF and CDX1 failed to induce *VIL1* promoter activity (Fig. 4I, J). Re-expression of CDX1 and ΔPNT-EHF also failed to induce expression of colonic differentiation markers, collectively demonstrating that interactions between EHF and CDX1 are important for driving colonic epithelial differentiation (Fig. 4K).

### Re-expression of EHF and CDX1 inhibits proliferation, motility and survival in vitro

**Fig. 5:**
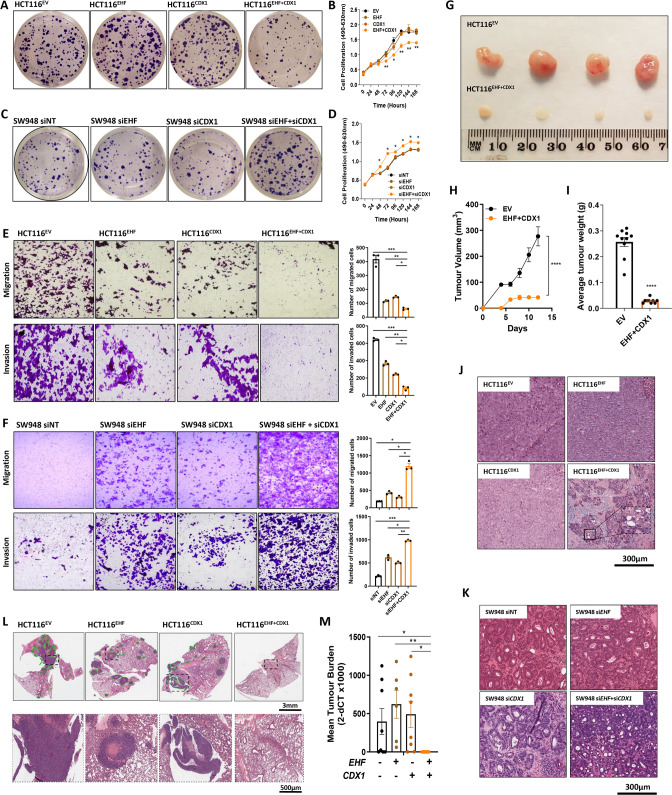
Re-expression of EHF and CDX1 promotes differentiation in poorly-differentiated CRC cells and inhibits tumour growth and metastasis in vitro and in vivo. **A**, **B** Effect of EHF and CDX1 re-expression in poorly-differentiated HCT116 cells on **A** colony formation and **B** cell proliferation determined by MTS assay. **C**, **D** Effect of EHF and CDX1 knockdown alone and in combination in moderately-differentiated SW948 cells on **C** colony formation and **D** cell proliferation determined by MTS assay. Colony formation was assessed 14 days after seeding. Values shown for MTS assay (**B**, **D**) are mean ± SEM of a representative experiment performed in technical quadruplicate. **E**, **F** Effect of EHF and CDX1 re-expression alone and in combination in HCT116 cells on cell migration and cell invasion. **F** Effect of EHF and CDX1 knockdown, alone and in combination, in moderately-differentiated SW948 cells on cell migration and invasion. Values shown in **E**, **F** are mean ± SEM of the number of migrated and invaded cells, respectively, after 24 h, experiments performed in biological triplicates. **G–I** HCT116 stably re-expressing EHF and CDX1, alone or in combination were grown as xenografts in immune compromised mice. **G** Representative tumours at endpoint of HCT116^EV^ and HCT116^EHF+CDX1^ cells. **H** Tumour volume was measured every second day for 15 days, and (**I**) tumour weight was determined at the experimental endpoint on day 15. Values shown are mean ± SEM of *n* = 5 mice, with 2 tumours injected per mouse (right and left flanks). **J** Histopathological assessment of differentiation grade in HCT116 xenografts following re-expression of EHF and CDX1 alone and in combination. **K** Histopathological assessment of differentiation grade in SW948 cells transfected with siRNAs targeting EHF and CDX1 alone and in combination. Mice were culled and xenografts removed 14 days post injection. **L**, **M** Effect of EHF and CDX1 re-expression on metastasis. HCT116 stably re-expressing EHF and CDX1, alone and in combination, were injected via the tail vein into NSG mice (*n* = 8 mice per isogenic line). Metastasis formation in the lung was determined after 8 weeks by **L** H&E staining of whole lungs or **M** quantitation of tumour burden by measuring human *Vimentin* DNA levels in the whole lung by q-RT-PCR. Values shown are mean ± SEM of whole lungs collected from *n* = 8 mice. *p < 0.05; **p < 0.01; ****p < 0.0001. Student’s *t* test in all cases.

To determine the functional consequences of transcriptional re-programming by EHF and CDX1, we assessed changes in cell proliferation, motility and survival. Whereas re-expression of EHF and CDX1 in poorly-differentiated HCT116 cells significantly reduced colony formation and proliferation (Fig. 5A, B), combined EHF/CDX1 knockdown in moderately-differentiated SW948 cells had the opposite effect (Fig. 5C, D).

Re-expression of EHF and CDX1, but not either factor alone, also inhibited migration and invasion of HCT116 cells (Fig. 5E), whereas combined EHF/CDX1 knockdown in SW948 cells had the opposite effect (Fig. 5F).

### Re-expression of EHF and CDX1 increases gland formation and reduces metastatic spread

To determine if the above observations are also evident in an in vivo setting, we performed xenograft studies which revealed that HCT116^EHF + CDX1^ cells grow significantly slower than HCT116^EV^ controls (Fig. 5G–I). Histological examination revealed that HCT116^EHF + CDX1^ tumours were more differentiated, evidenced by increased gland formation and expression of the differentiation markers VIL1 and KRT20, compared to HCT116^EV^, HCT116^EHF^ or HCT116^CDX1^ tumours (Fig. 5J, S5A). Conversely, combined knockdown of *EHF* and *CDX1* in SW948 resulted in xenografts that had increased numbers of smaller, polyformed glands, and reduced expression of VIL1 and KRT20 (Fig. 5K, S5B).

Next, we assessed the impact of EHF/CDX1 re-expression on metastasis by injecting HCT116^EV^, HCT116^EHF^, HCT116^CDX1^ and HCT116^EHF + CDX1^ cells into the tail vein of NOD/SCID gamma mice, and monitoring formation of lung metastases. Consistent with their reduced cell motility and invasiveness in vitro, HCT116^EHF+CDX1^ cells generated significantly fewer lung metastasis compared to cells expressing the vector control or EHF or CDX1 alone (Fig. 5L, M).

### Compound deletion of Ehf and Cdx1 in the mouse colon accelerates colonic tumour formation

**Fig. 6:**
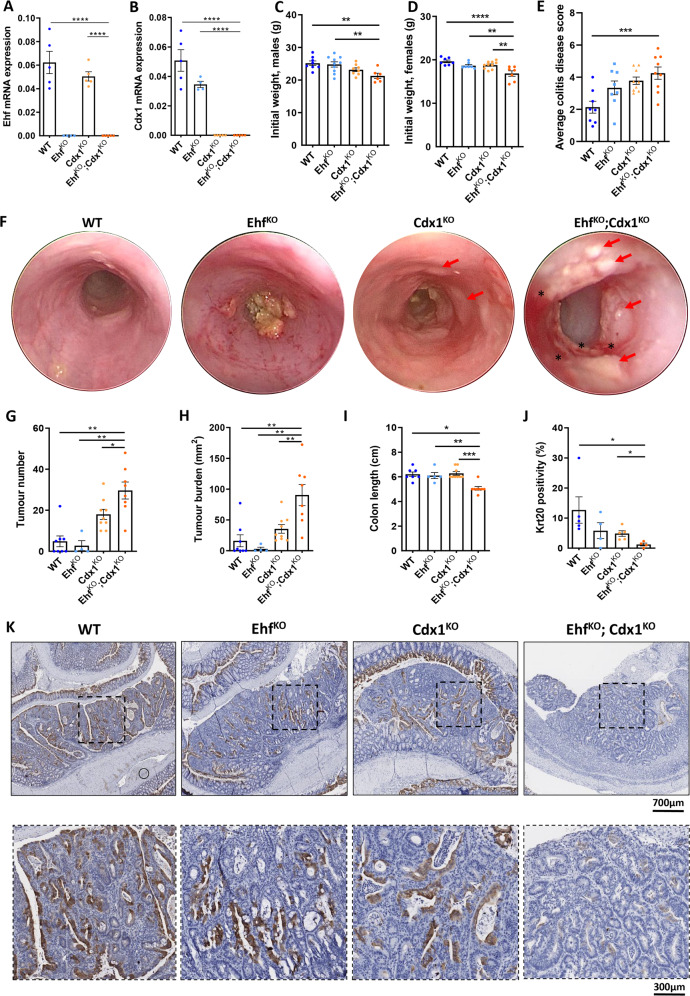
*Ehf/Cdx1* deletion increases susceptibility of mice to AOM/DSS-induced colorectal tumourigenesis. **A**, **B** Confirmation of (**A**) *Ehf* and (**B**) *Cdx1* knockout in the colonic epithelium of *Ehf* and *Cdx1* knockout mice. Colonic epithelial cells were isolated from 6-week-old *WT*, *Ehf*^*KO*^, *Cdx1*^*KO*^ and *Ehf*^*KO*^*;Cdx1*^*KO*^ mice and *Ehf* and *Cdx1* mRNA determined by q-RT-PCR. Values shown are mean ± SEM of *n* = 5 mice. **C**, **D** Body weights of 8-week-old (**C**) male and (**D**) female *WT* (*n* = 7 and *n* = 7 for male and female cohorts, respectively), *Ehf*^*KO*^ (*n* = 9 and *n* = 7), *Cdx1*^*KO*^ (*n* = 7 and *n* = 10) and *Ehf*^*KO*^*;Cdx1*^*KO*^ (*n* = 6 and *n* = 7) mice. Values shown are mean ± SEM. **E** Average colitis disease score of *WT* (*n* = 8), *Ehf*^*KO*^ (*n* = 8), *Cdx1*^*KO*^ (*n* = 9) and *Ehf*^*KO*^*;Cdx1*^*KO*^ (*n* = 9) mice following the second round of DSS treatment. Values shown are mean ± SEM. **F** Representative endoscopy images from *WT*, *Ehf*^*KO*^, *Cdx1*^*KO*^ and *Ehf*^*KO*^*;Cdx1*^*KO*^ mice showing inflammation (*) and colon tumour formation (arrows) in *Cdx1*^*KO*^ and *Ehf*^*KO*^*;Cdx1*^*KO*^ mice. Endoscopies were performed in 13-week-old mice after receiving 2 injections of AOM and 2 rounds of DSS. **G**–**I** Quantitation of (**G**) tumour number, **H** overall tumour burden and (**I**) colon length in *WT* (*n* = 7), *Ehf*^*KO*^ (*n* = 4), *Cdx1*^*KO*^ (*n* = 9) and *Ehf*^*KO*^*;Cdx1*^*KO*^ (*n* = 8)mice at endpoint. Values shown are mean ± SEM, with some *Ehf*^*KO*^ having to be euthanized for non-tumour related phenotypes (genital abscess). **J** Quantitation of KRT20 staining in tumours derived from *WT* (*n* = 5), *Ehf*^*KO*^ (*n* = 4), *Cdx1*^*KO*^ (*n* = 5) and *Ehf*^*KO*^*;Cdx1*^*KO*^ (*n* = 4) mice. Values shown are mean ± SEM. **K** Representative immunohistochemistry images in the colon from *WT, Ehf*^*KO*^*, Cdx1*^*KO*^ and *Ehf*^*KO*^*;Cdx1*^*KO*^ mice stained with anti-KRT20 antibody. *p < 0.05; **p < 0.01; ***p < 0.001; ****p < 0.0001, one-way ANOVA with Tukey’s post hoc test.

To extend the above studies, we determined the impact of co-ordinate loss of EHF and CDX1 on colon tumourigenesis in vivo, by generating compound *Ehf* /*Cdx1* knockout mice (*Ehf* ^*KO*^*;Cdx1*^*KO*^), in which loss of *Ehf* and *Cdx1* expression in colonic epithelial cells was confirmed by q-RT-PCR (Fig. 6A, B).

*Ehf* ^*KO*^*;Cdx1*^*KO*^ mice were born at the expected Mendelian ratio, but both male and female *Ehf* ^*KO*^*;Cdx1*^*KO*^ mice had significantly lower body weight than WT mice, or mice lacking either transcription factor alone (Fig. 6C, D). Expression of the enterocytic differentiation markers *Vil1*, *Gpa33* and *Cdh17* and colonic stem cell marker *Lgr5* were also significantly reduced in *Ehf* ^*KO*^*;Cdx1*^*KO*^ mice compared to WT controls (Fig. S6), consistent with the requirement for these factors in maintaining normal colonic differentiation.

Next, to determine if *Ehf* and *Cdx1* loss affects colonic tumourigenesis, wild-type, *Ehf* ^*KO*^*, Cdx1*^*KO*^ and *Ehf* ^*KO*^*;Cdx1*^*KO*^ mice were treated with the carcinogen azoxymethane (AOM) followed by two cycles with the inflammation-inducing agent dextran sodium sulphate (DSS). *Ehf* ^*KO*^*;Cdx1*^*KO*^ mice displayed the highest sensitivity to the DSS-induced colitis as evidenced by a higher disease score, and greater body weight loss, reaching an ethical endpoint significantly more rapidly compared to control of single gene deleted mice (Fig. 6E).

Endoscopic analyses prior to endpoint revealed that loss of *Cdx1* alone was sufficient to increase colon tumour formation, which was further increased in mice with compound *Cdx1* and *Ehf* deletion (Fig. 6F). Consistent with these findings, tumour number and burden were increased in *Cdx1*^*KO*^ mice compared to wild-type controls and *Ehf* ^*KO*^ mice. Strikingly however, a further increase in both tumour number and burden was observed in compound *Ehf* ^*KO*^*;Cdx1*^*KO*^ mice compared to all other genotypes (Fig. 6G, H, S7). *Ehf* ^*KO*^*;Cdx1*^*KO*^ mice also had a significantly shorter colon length compared to wild-type controls or *Ehf* and *Cdx1* single deletion mice (Fig. 6I). To determine if *Ehf/Cdx1* deletion altered expression of differentiation markers, an additional cohort of mice were aged until tumours development in all genotypes. IHC staining for the differentiation marker KRT20 revealed a significant reduction of positive staining in both the normal colonic mucosa as well as in tumours arising in *Ehf* ^*KO*^*;Cdx1*^*KO*^ mice compared to WT mice or mice lacking either factor alone (Fig. 6J, K). Taken together, these data indicate that EHF and CDX1 are critical regulators of CRC differentiation and suppressors of tumour progression.

### EHF and CDX1 are co-ordinately methylated in poorly-differentiated CRCs

**Fig. 7:**
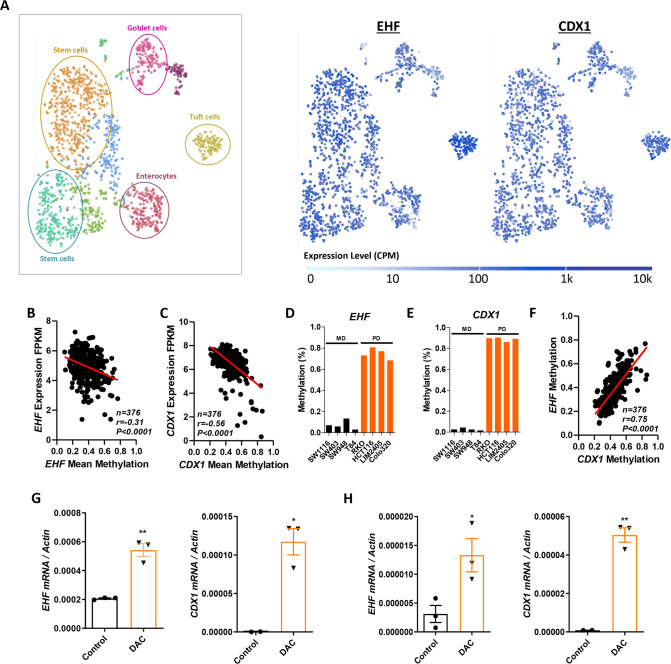
EHF and CDX1 have overlapping expression profiles across intestinal epithelial cell subtypes and are co-ordinately methylated in CRCs. **A** t-SNE plots of EHF and CDX1 mRNA expression in different intestinal cell types determined by interrogation of the Haber et al. dataset [20] using the single cell expression atlas. **B**, **C** Pearson’s correlation of **B** EHF (cg05503887, cg18414381 and cg18560551) and **C** CDX1 (cg11524248, cg24216701, cg25132276, cg26531174 and cg11117637) mean promoter methylation and mRNA expression in primary colorectal tumours (*n* = 376). Data obtained from the TCGA portal. **D**, **E** Methylation profiling of the (**D**) EHF gene body and **E** CDX1 promoter in four moderately-differentiated (MD) and four poorly-differentiated (PD) CRC cell lines determined using human methylation arrays. Value shown are the mean methylation value (Beta values) from a single methylation profiling array of each cell line. **F** Pearson’s correlation of EHF and CDX1 methylation in primary colorectal tumours from the TCGA cohort (*n* = 376). **G**, **H** Effect of 5-aza-2’-deoxycytidine (Decitabine, DAC) treatment (1 µM) on EHF and CDX1, and differentiation marker mRNA expression in **G** HCT116 and **H** RKO cells. q-RT-PCR data shown are mean ± SEM from a representative experiment performed in triplicate. *p < 0.05; **p < 0.01, Student’s *t* test.

We next asked if EHF and CDX1 are co-ordinately expressed in the normal intestinal epithelium, and in primary CRCs. Analysis of publicly available single cell RNA-seq data from the normal mouse intestine [20] revealed the co-expression of both transcription factors in intestinal stem cells, enterocytes, goblet cells, tuft cells and enteroendocrine cells (Fig. 7A).

Given that *EHF* and *CDX1* are rarely mutated in CRCs, we examined whether loss of their expression in poorly-differentiated CRCs may occur epigenetically. Integration of methylation and corresponding gene expression data from primary CRCs profiled by the TCGA revealed a significant inverse correlation between *EHF* promoter methylation and mRNA expression (Fig. 7B). Similarly, we observed a significant inverse correlation between CDX1 promoter methylation and mRNA expression (Fig. 7C). Remarkably, *EHF* and *CDX1* were co-ordinately methylated in poorly-differentiated CRC cell lines (Fig. 7D, E), and across 376 primary CRCs profiled by the TCGA, indicating these factors are co-ordinately methylated in CRC (Fig. 7F).

Finally, to determine if promoter methylation contributes to EHF and CDX1 silencing in CRC, we treated the poorly-differentiated HCT116 and RKO cells with the de-methylating agent 5-aza-2′-deoxycytidine (Decitabine), which significantly increased mRNA expression of both EHF and CDX1 (Fig. 7G, H). These results suggest that epigenetic silencing is a key mechanism underlying co-ordinate loss of these transcription factors in poorly-differentiated CRCs.

## Discussion

Tumour de-differentiation is a fundamental feature of human CRCs is associated with poorer patient outcome and reduced response to chemoradiation therapy [8, 9]. However, how de-differentiation occurs, and whether it is a driver or consequence of tumour progression is not fully understood. Here, using isogenic CRC cell lines and *Ehf* /*Cdx1* compound deletion mice we provide direct evidence that the ETS family transcription factor, EHF, and the caudal homeobox transcription factor, CDX1, co-operate to control the differentiation state of CRCs, and that their co-ordinate epigenetic silencing in a subset of poorly-differentiated CRCs drives colorectal tumour progression. These findings provide new insight into the molecular basis underlying the emerging paradigm that CRC progression is driven by loss of key drivers of colonic differentiation [21, 22].

Our finding that CRCs that have lost EHF and CDX1 expression also lose expression of colonic stem cell markers, and the capacity of these factors to functionally co-operate to drive re-expression of these markers, also highlights a novel mechanism for maintaining colonic identity. The robust capacity of these two factors to drive colonic identity is also underscored by their ability to drive colonic differentiation markers in cells of non-colonic origin.

By interrogating the mechanism by which these factors regulate *VIL1* expression we also unveil a specific transcriptional regulatory model whereby CDX1 directly binds to the VIL1 promoter and subsequently recruits EHF to enhance expression. Notably, a similar model has been proposed for the related homoeodomain protein CDX2, which directly binds to chromatin and maintains transcription-permissive chromatin in the mouse intestine, and subsequently recruits transcription factors such as HNF4A to drive target gene expression [23]. We also note however that EHF and CDX1 may function by additional mechanisms at different loci, including direct binding of EHF to regulatory elements to control gene expression, or the induction of chromatin looping events mediated by both the direct DNA biniding and physical interaction between these transcription factors.

The convergence of multiple master transcription factors to enable chromatin access and orchestrate transcriptional networks is emerging as a key mechanism driving lineage-specific differentiation. For example, enhancer profiling has identified interconnected transcriptional regulatory networks formed by multiple master transcription factors in oesophageal [24], lung [25] and subtypes of pancreatic cancer [26]. Furthermore, multiple transcription factor networks involving *CDX2* have been implicated in driving normal intestinal cell differentiation [23, 27–29]. Interestingly, we observed an increase in CDX2 expression following EHF and CDX1 re-expression in CRC cells, suggesting CDX2 may be a target of EHF/CDX1, although this needs to be further investigated.

Our findings in CRC cell lines revealed that the induction of differentiation promoted by EHF/CDX1 re-expression invoked a subsequent reduction in cell proliferation and cell survival, reflecting the phenotypic changes normal colonic epithelial cells undergo as they migrate along the crypt axis towards the luminal surface. These findings highlight a potential mechanism whereby differentiation loss may contribute to colonic tumour formation by enabling normal colonic epithelial cells to evade their inherent terminal differentiation programme. Differentiation loss may also contribute to tumour initiation and/or progression by additional mechanisms. For example, normal differentiated colonic epithelial cells are exposed to multiple environmental insults including those derived from dietary factors and the microbiota. While normal colonocytes have evolved mechanisms to safeguard against the damage induced by these factors such as the expression of high levels of xenobiotic detoxifying enzymes and mucins, the loss of differentiation may impact the expression of these factors in turn increasing the risk of DNA damage and transformation. Additional studies are needed to explore the relative contributions of these differentiation-associated processes in the progression of CRC, and may provide important insight into the potential of differentiation therapy in the prevention of CRC.

An important finding of this study is the identification of co-ordinate methylation of the *EHF* and *CDX1* promoters, which may drive their loss of expression in poorly-differentiated CRCs. Loss of transcription factor binding has been postulated to be a potential mechanism enabling promoter methylation [30], raising the possibility that the co-ordinate methylation of *EHF* and *CDX1* may be triggered by loss of a common upstream transcription factor. Further elucidation of the signalling and transcriptional events which regulate EHF and CDX1 expression may therefore further unveil the hierarchy of differentiation loss in CRC, and uncover potential therapeutic strategies for CRC based on the re-induction of EHF/CDX1-driven tumour differentiation.

In summary, this study reveals a novel mechanism of CRC progression triggered by the co-ordinate epigenetic inactivation of the EHF and CDX1 transcription factors and loss of colonic differentiation.

## Material and methods

### Cell culture and reagents

Human CRC cell lines SW1116, SW948, SW403, T84, RKO, HCT116, Colo320, DLD1, LOVO, HCC2998, SW48, SW480, VACO5, KM12, ALA, HT29, Co115, Colo201, Colo205 and LS513 were obtained from the American Type Culture Collection (ATCC, Manassas, VA, USA) and LIM2405 and LIM1215 were obtained from The Ludwig Institute for Cancer Research Melbourne. The retroviral packaging cell line Phoenix-Ampho was also obtained from ATCC. Cell line authentication was performed on parental and isogenic lines using the GenePrint^®^ 10 System (Promega, Madison, WI, USA) and Fragment Analysis, and all cell lines for which reference short tandem repeat (STR) profiles existed produced close matches confirming their authenticity. Cells were routinely tested for mycoplasma status by the MycoAlert Mycoplasma Detection Kit (Lonza, Basel, Switzerland) and confirmed to be negative.

All cell lines were maintained at 37 °C and 5% CO_2_ in base medium Dulbecco’s Minimal Essential Media DMEM F12 supplemented with 10% FCS (v/v), 2mM L-glutamine, 100 U/mL Penicillin and 100 µg/mL Streptomycin all from Thermo Fisher Scientific (Waltham, MA, USA). 5-aza-2′-deoxycytidine (DAC) was purchased from Sigma-Aldrich (St. Louis, MO, USA).

### Plasmids and viral vectors

MSCV-EHF was generated by sub-cloning EHF cDNA from the pcDNA3.1-EHF plasmid kindly provided by Dr. Giuseppina Carbone [13]. Full-length EHF cDNA was excised from the XhoI restriction sites in the multiple cloning sites of pcDNA3.1 and cloned into MSCV-IRES-GFP vector. The EHF-ΔPNT construct was generated by first excising the wild-type POINTED domain (258 bp) from the MSCV-EHF plasmid at the MfeI and BglII restriction sites. A shorted POINTED domain with a premature STOP codon (150 bp) was cloned into the MfeI and BglII restriction sites in place of the wild-type POINTED domain.

### Stable cell line generation

HCT116 and RKO cells stably expressing EHF were generated by transfecting 5 μg of *EHF* expression plasmid (pcDNA3.1-EHF) or empty vector control. Stably transfected lines were selected by isolating clones resistant to 1000 μg/mL and 600 μg/mL geneticin (G418) for HCT116 and RKO cells, respectively.

HCT116 cells stably expressing CDX1 (HCT116^CDX1^) were generated by retroviral transduction with MSCV-IRES-GFP-CDX1 which was kindly provided by Dr. Nicholas Clemons. The GFP positive subset was sorted by fluorescence activated cell sorting (FACS) and expanded.

HCT116 cells overexpressing both *EHF* and *CDX1* were generated by transfecting HCT116^CDX1^ cells with 5 μg of *EHF* expression plasmid as above and selection of geneticin-resistant clones.

### Transient transduction

The retroviral packaging cell line Phoenix-Ampho was transfected with 5 μg of MSCV-IRES-GFP-EHF or MSCV-IRES-GFP-CDX1 expression vectors for 24 h using Lipofectamine. HCT116, LIM2405, and SW480 cell lines were then transduced retrovirally for 48 h and the entire cell population lysed in NP-40 buffer.

### Quantitative RT-PCR

Total RNA was purified using the High Pure RNA Isolation Kit (Roche, Germany) and reverse transcribed using random hexamers from the Transcriptor High Fidelity cDNA Synthesis Kit (Roche), as per manufacturer’s protocol. Gene expression was determined by quantitative RT-PCR using Power SYBR Green PCR Master Mix (Applied Biosystems, USA) on the ViiA 7 Real Time PCR system (Applied Biosystems) according to manufacturer’s protocol. 10 ng of cDNA was amplified with 75 nM forward and reverse primers in a 5 µL reaction. Primers used are listed in Table S1.

### Western blot analysis

Cell lines were lysed in NP-40 buffer (1% NP-40/IGEPAL, 50 mM Tris-HCl, 150 mM NaCl, 0.5% Sodium Deoxycholate, 1 mM EDTA), containing protease inhibitor cocktail Roche cOmplete and phosphatase inhibitor PhosSTOP. Nuclear extracts were prepared by lysing cell pellets in cytosolic lysis buffer (10 mM HEPES buffer, 1.5 mM MgCl_2_, 10 mM KCl, 0.5 mM DTT, 0.05% NP-40) and centrifuged at 17,000 × *g* for 20 min. Pelleted nuclei were then lysed in nuclear buffer (5 mM HEPES buffer, 1.5 mM MgCl_2,_ 0.2 mM EDTA, 0.5 mM DTT, 26% Glycerol). Proteins were separated on NuPAGE 4–12% Bis-Tris pre-cast polyacrylamide gels (Novex, Thermo Fisher Scientific) and transferred to PVDF membranes (Life Technologies). Membranes were blocked using Odyssey PBS Blocking Buffer (LI-COR, Lincoln, NE, USA) for 60 min and incubated with primary antibodies overnight at 4 °C. Primary antibodies and dilutions used were: EHF (Abcam, ab126963, 1:500), CDX1 (Abcam, ab88148, 1:1000), FLAG-DYKDDDDK Tag (CST, 8146, 1:1000), Villin-1 R814 (CST, 2369S, 1:1000), GPA33 EPR4240 (Abcam, ab108938, 1:1000), KRT20 D9Z1Z (CST, 13063, 1:2500), LI-Cadherin (CDH17) EPR3997 (Abcam, ab109220, 1:1000), HDAC-1 C-19 (Santa Cruz, SC-6298, 1:500) and β-Actin (Sigma, A5316, 1:20,000). The membranes were then incubated with fluorescent IRDye secondary antibody (LI-COR) of the matching species and visualised using the Odyssey Classic Infra-red Imaging System and Odyssey software (LI-COR). Full-length original western blots are provided in Supplementary File 1.

### Chromatin immunoprecipitation (ChIP) assay

HCT116^EV^, HCT116^EHF^, HCT116^CDX1^ and HCT116^EHF + CDX1^ cells were seeded on a 10 cm tissue culture dish at a density of 2 × 10^6^ and incubated overnight at 37 °C. Formaldehyde was added to the cells to a final concentration of 1%, incubated for 10 min, and quenched with glycine at a final concentration of 0.125 M. Cells were then rinsed with PBS, collected by centrifugation and lysed in cell lysis buffer (Millipore, Massachusetts, USA) on ice for 10 min. The nuclei pellet was then collected by centrifugation and lysed in nuclear lysis buffer (Millipore, Massachusetts, USA) on ice for 10 min. Sonication was then performed to shear the chromatin for 20 min (10 s on and 10 s off). Samples were then incubated with anti-FLAG, CDX1 or IgG antibodies with rocking at 4 °C, and 20 µL of magnetic protein A/G beads (Millipore, Massachusetts, USA) were added to each sample and incubated overnight. The magnetic beads were washed with low salt immune complex, high salt immune complex, LiCl and TE buffer, respectively, and DNA was eluted in elution buffer (1% SDS and 0.1 M NaHCO_3_). The samples were then reverse cross-linked with 5 M NaCl at 65 °C for 4 h, then the DNA was purified by column centrifugation for q-RT-PCR analysis. Re-ChIP analysis was performed on cross-linked chromatin from HCT116^EV^, HCT116^EHF^, HCT116^CDX1^ and HCT116^EHF + CDX1^ cells prepared from the ChIP assay described above. Samples were incubated with an anti-FLAG antibody and magnetic protein A/G beads overnight at 4 °C, then washed and eluted. Eluted chromatin was then diluted and subjected to a second immunoprecipitation with anti-CDX1 antibody and magnetic protein A/G beads overnight at 4 °C. The samples were then washed, eluted, reverse cross-linked and purified for q-RT-PCR analysis as described above. Primers used are listed in Table S1.

### Immunoprecipitation

For the detection of FLAG-EHF and CDX1 interaction, HCT116 cells transfected with either empty vector, EHF, CDX1, Δ-EHF-PNT expression constructs or the combination were lysed in NP-40 buffer, containing protease inhibitor cocktail Roche cOmplete and phosphatase inhibitor PhosSTOP. Whole cell lysates were incubated with Pierce^TM^ Protein A/G Plus Agarose beads (Thermo Fisher) and either CDX1 (Abcam, ab88148, 1:1000) or FLAG-Tag 9A3 (CST, 8146S, 1:1000). Immunoprecipitates were then washed in NP-40 lysis buffer, boiled in sample buffer and ran on NuPAGE 4–12% Bis-Tris pre-cast polyacrylamide gels (Novex, Thermo Fisher Scientific) and transferred to PVDF membranes (Life Technologies). Membranes were blocked using Odyssey PBS Blocking Buffer (LI-COR, Lincoln, NE, USA) for 60 min and incubated with primary antibodies overnight at 4 °C. The membranes were then incubated with fluorescent IRDye secondary antibody (LI-COR) of the matching species and visualised using the Odyssey Classic Infra-red Imaging System and Odyssey software (LI-COR).

### RNAi-mediated knockdown

Individual ON-Target-Plus small interfering RNAs (siRNAs) targeting different regions of genes of interest were purchased from GE Dharmacon (Colorado, USA) and reconstituted in 1X siRNA buffer (Thermo Scientific, USA) to a concentration of 20 μM. siRNA transfection was performed using Lipofectamine RNAiMAX (Life Technologies) according to manufacturer’s instructions, using a reverse transfection protocol. Briefly, the siRNA/Lipofectamine complex was incubated for 20 min at room temperature and added to cell lines to a final siRNA concentration was 30 nM, and cells incubated at 37 °C for 24 h and changed into fresh media for another 24 h. The siRNAs used are listed in Table S2.

### Dual-luciferase reporter assay

Promoter reporter activity was measured using the Dual-Luciferase Reporter assay System (Promega). HCT116 and RKO cells were transfected with either the pGL3-basic or pVIL1-Luc promoter reporter constructs for 72 h in the presence or absence of an EHF and CDX1 expression construct. Transfected cells were lysed in 1X passive lysis buffer (Promega).

### Cell proliferation and colony formation assays

Cell proliferation was measured by MTS assay by seeding 5000 cells/per well in a volume of 100 μL in 96-well plates. At the end of the experimental period, cells were treated with 20 µL of 2 mg/mL MTS/PMS solution, incubated for 90 min at 37 °C, and absorbance at 630 nm and 490 nm was measured using a microplate reader.

Colony forming capacity were determined by seeding 500 cells per well of a 6-well plate. Colony formation was allowed to occur over 14 days, with the medium replenished every 3 days. Once colonies had formed, plates were washed in PBS and colonies fixed in 10% buffered formalin for 5 min at room temperature. Colonies were then washed and stained with 0.1% crystal violet solution (Sigma-Aldrich) for 15 min at room temperature. Following this step, colonies were washed twice in PBS and allowed to air dry. Plates were analysed for the number for colonies established using the ImageJ software.

### Cell migration and invasion assays

Cell migration and invasion assays were assessed using 8.0 µm pore size Boyden-Chamber transwells (Corning). For cell invasion assays, chambers were first pre-coated with 20 µL (1:10 dilution) of low serum Matrigel (Cultrex) and allowed to set for 60 min. Cells were serum starved overnight and then seeded into the upper chamber (2 × 10^4^ cells) in 500 µL serum free medium. 500 µL medium with 10% FCS was added to the lower chambers. After 24-h, non-migrated/non-invasive cells in the upper chamber were removed using a cotton swab and migrating/invasive cells were fixed in 10% buffered formalin and stained with 0.1% crystal violet. The number of migrated/invaded cells was analysed using the ImageJ software.

### Xenograft studies

All animal studies were performed with the approval of the Austin Health Animal Ethics Committee. 8-week-old male Balb/c *nu/nu* mice were obtained from the Animal Resources Centre (ARC, Perth, Australia) and maintained in specific pathogen free (SPF) microisolators. SW1116, SW403, SW948, T84, RKO, HCT116, LIM2405, Colo320, HCT116^EV^, HCT116^EHF^, HCT116^CDX1^ and HCT116^EHF + CDX1^, SW948-NT, SW948-siEHF, SW948-siCDX1 and SW948-siEHF+siCDX1 (2 × 10^6^ cells) CRC cell lines were injected subcutaneously into the left and right flank of each animal in a 150 µL suspension of 1:1 mixture of DMEM:Matrigel Basement Matrix (BD Sciences). Ten mice were used per isogenic cell line. Tumour growth was monitored three times per week by caliper measurement and tumour volume calculated according to the formula pi/6 × large diameter × small diameter^2^. Once the tumours reached a size of 1 cm^3^, animals were euthanized, and tumours were fixed in 10% formalin and paraffin embedded.

### Endoscopy procedure

Mice were anaesthetised using (1 L O_2_/min and 2–3% Isoflurane. The procedure was performed using a miniature endoscope (scope 1.9 mm outer diameter), a light source, an IMAGE 1 camera, and an air pump (Karl Storz, Germany), to achieve regulated inflation of the mouse colon.

### Metastatic burden assay

All mice used were 8-week-old male NOD SCID Gamma (NSG) mice bred in-house at the Austin Health Bio Resources Facility and were maintained in specific pathogen free microisolators. HCT116^EV^, HCT116^EHF^, HCT116^CDX1^ and HCT116^EHF + CDX1^ cells (1 × 10^5^) were intravenously injected into the tail vein in 100 µL of PBS. After 8 weeks, mice were euthanized and whole lungs were formalin fixed and paraffin embedded. A section of lung was also fresh frozen and genomic DNA (gDNA) extracted by homogenising in digestion buffer (100 mM NaCl, 10 mM Tris-HCl pH 8.0, 25 mM EDTA, 5% SDS) overnight at 55 °C. The lung digests were purified with Phenol:Chloroform:Isoamyl Alcohol (IAA) solution (24:24:1) and the dried gDNA pellets resuspended in TE buffer. Metastatic burden was determined microscopically by counting the number of tumour nodules and measurement of the relative levels of human vimentin and mouse Rsp27a DNA in a whole lung by q-RT-PCR.

### *Ehf* and *Cdx1* knockout mice

Mice with floxed Ehf were generated by flanking exon 8 and 9, containing the ETS domain, with loxP sites in C57Bl/6 embryonic stem (ES) cells. The targeting vector contained a branch site, splice acceptor and a neomycin cassette for selection in ES cells, flanked by FRT sites for Flp-recombinase-mediated removal of the neomycin cassette. Positive ES clones were selected based on neomycin resistance, microinjected into C57Bl/6 murine blastocysts, and implanted into pseudo-pregnant females. Resulting chimeras were bred to wild-type C57Bl/6 mice to generate mice heterozygous for the floxed allele. Heterozygous mice were then crossed with OzFLP mice to induce removal of the neomycin cassette. Mice heterozygous for the floxed Ehf allele were mated to generate homozygous Ehf^lox/Lox^ mice. Ehf^lox/lox^ mice were subsequently mated with C57Bl/6 CMVCre-deleter mice, Original Source: Mouse Genetics Cologne (MGC) Foundation and provided to us by Prof. Warren Alexander at the Walter and Eliza Hall Institute (Melbourne, Australia), to induce constitutive Ehf deletion. CMVCre was then bred out of the colony to produce germline Ehf^KO^ mice.

B6.129-Cdx1^*tm1Pgr*^/Mmucd (Cdx1^KO^) mice, RRID:MMRRC_012031-UCD, were obtained from the Mutant Mouse Resource and Research Centre (MMRRC) at University of California at Davis, an NIH-funded strain repository; donated to the MMRRC by Kevin Haigis, Ph.D., Massachusetts Institute of Technology.

Germline Ehf homozygous KO mice were bred to Cdx1 homozygous KO mice to produce heterozygous F1 offspring, F1 offspring were then bred to generate F2 consisting of the four experimental genotypes, Ehf^+/+^;Cdx1^+/+^, Ehf^KO^;Cdx1^+/+^, Ehf^+/+^;Cdx1^KO^ and Ehf^KO^;Cdx1^KO^ on a mixed C57BL/6 × 129 genetic background.

2–3 mm of mouse tail biopsy was collected at weaning for genotyping purposes. DNA extraction was performed by incubation in 30 µl QuickExtract DNA extraction solution (Lucigen) at 65 °C for 15 min followed by 95 °C for 5 min. 2 µl of DNA was used in the genotyping reaction. MyTaq™ Red DNA Polymerase (Bioline) was used for gene amplification.

Primers used for DNA genotyping were as follows: CMVCre (F): CTGACCGTACACCAAAATTGCCTG, (R): GATAATCGCGAACATCTTCAGGTT. Exon 8 (outside floxed region)-intron 6 Ehf (F): GTCCAAAATGAAGCCCAGGGTA, Ehf (R): CGTCCGGTTCTTCATTGATCAG. Exon 8 (inside floxed region)-intron 6 Ehf (F): TGTGTCTTGCTTTCCACCAG, (R): CGTCCGGTTCTTCATTGATCAG. Cdx1^KO^ genotyping primers as per MMRRC recommendation: Cdx1 (Neo): ATGAGACGAGCCGATTGTCTGTTGTGC. (F1): GGAAGGGCAGTCACAGAACACGGAG. (R1): ACACAGCCCAGCCAACGGAGG.

The resultant products were run on a 1.5% Agarose gel (Bioline) with SYBR Safe DNA gel stain added (Invitrogen) at 100 V for 70 min for band separation and genotype determination.

All animal breeding and procedures were performed in concordance with approval obtained from the Animal Ethics Committee, Austin Health (Melbourne). All animals were housed in open-top boxes with basic enrichment and provided with standard mouse chow and drinking water *ad libitum*.

### Azoxymethane/Dextran sodium sulphate (AOM/DSS) induced colitis associated colorectal cancer

Ehf^+/+^;Cdx1^+/+^, Ehf^KO^;Cdx1^+/+^, Ehf^+/+^;Cdx1^KO^ and Ehf^KO^;Cdx1^KO^ mice (8 weeks old) were intraperitoneally injected with 2 doses of azoxymethane (AOM) at 10 mg/kg, a week apart. Mice were then given 1% dextran sodium sulphate (DSS) in the drinking water for 5 days, then returned to normal drinking water for 2 weeks, for a total of two cycles. Immediately following periods of treatment with DSS, when animals are returned to normal drinking water, is the anticipated peak of severity of colitis; during this time, animals were supplemented with wet mashed chow and vanilla sustagen, and placed on a heat pad. Mice were monitored closely, and disease severity scored according to a set of criteria that included general appearance, weight loss, stool consistency and presence of blood in the stool. After the last cycle of DSS, mice were euthanized and the whole colon (Swiss rolls) fixed in 10% formalin and paraffin embedded. Investigators undertaking the animal monitoring were blinded to the genotype of the mice, and *WT* (*n* = 8), *Ehf* ^*KO*^ (*n* = 8), *Cdx1*^*KO*^ (*n* = 9) and *Ehf* ^*KO*^*;Cdx1*^*KO*^ (*n* = 9) mice were analysed.

### Immunohistochemistry

Tissue sections (4 μm) were cut and placed onto electrostatic glass slides and processed by deparaffinising, rehydrating and quenching of endogenous peroxidase. Antigen retrieval was performed by boiling slides in 50 mM Tris-HCl (pH 8.5) (Dako) for 45 min. Primary Antibodies and dilutions used were: Villin-1 R814 (CST, 2369 S, 1:200) and KRT20 D9Z1Z (CST, 13063, 1:250). Antibodies were added to the slides and incubated overnight at 4 °C in a humidified chamber. Slides were then washed in PBS and incubated with Dako envision anti-rabbit labelled polymer-HRP (Dako) secondary antibody for 30 min at room temperature. Slides were washed again in PBS and stained with DAB solution (3,3-diaminobenzidine) for 30–60 s with colour development carefully monitored by eye. Slides counterstained using pre filtered Mayer’s haematoxylin (Amber Scientific, Australia) and then dehydrated and sealed with glass coverslips using DPX mounting solution (Sigma-Aldrich). The presence of tumours in the colon was histologically confirmed by anatomical pathologist (DSW) by examination of haematoxylin and eosin (H&E) stained sections.

### ATAC-seq Library preparation

ATAC-seq was performed as described previously [31]. Nuclei were extracted from 50,000 cells per condition by resuspending in 50 µl of ATAC-seq lysis buffer (10 mM Tris-HCl (pH 7.4), 10 mM NaCl, 3 mM MgCl_2_, 0.1% IGEPAL). Digestion of the DNA (transposition) and ligation of adapters was performed according to the manufacturer’s instructions as provided in the Nextera kit (Illumina, FC-121-1030). Briefly, DNA was digested by incubation with Tn5 Transposase enzyme at 37 °C for 30 min. Transposed DNA was then purified using the Qiagen MinElute PCR purification kit (Qiagen, 28004), and PCR-amplified using Nextera custom barcoded primers for 5 cycles. To reduce GC-bias, the number of additional PCR cycles required was determined using SYBR Green q-RT-PCR reaction by calculating the number of cycles that correspond to 1/3 of the maximum fluorescent intensity as described by Buenrostro et al. [31]. Following sample amplification for the optimised number of additional PCR cycles, libraries were purified using Qiagen MinElute PCR purification kit and DNA quantity and quality was analysed using the Tapestation 2000 (Agilent). All libraries were sequenced on the Illumina NextSeq500 with pair end 80 bp reads.

ATAC-seq reads were mapped to the GRCh38/hg38 build of the human genome using the Subread aligner [32]. Only uniquely mapped reads were retained. ATAC peaks were called using Homer [33] with a false discovery rate (FDR) cutoff of 10^−5^.

Peaks called from different samples were merged to form a set of unique regions that covered all the peaks called from each sample. Mapped reads were then assigned to these regions using the featureCounts programme [34]. Read counts were generated from each region in each sample. Regions were excluded from analysis if they failed to achieve a CPM (counts per million) value of at least 3 in at least 1 sample. Counts for regions were converted to log_2_CPMs, quantile-normalised and precision weighted with the limma voom function [35, 36]. When assessing the chromatin accessibility around the transcriptional start site (TSS) of each gene, we divided into 50 bp intervals the 5 kb region upstream from the TSS of each gene and also the 5 kb region downstream from the TSS. We then counted the number of reads overlapping with each interval for each gene and converted the counts to log_2_CPM values using all the counts generated for these intervals. The log_2_CPM values were then transformed to Z-scores and displayed in the heatmaps.

### RNA-sequencing

 RNA was isolated from HCT116^EV^, HCT116^EHF^, HCT116^CDX1^ and HCT116^EHF+CDX1^ and RNA samples were quantified with High-sensitivity assays (Invitrogen, USA) using a Qubit 4.0 Fluorometer (Invitrogen, USA) and the quality of the RNA is accessed on 4200 TapeStation System (Agilent, USA).

For each sample, equal amount of RNA was pooled from different tissue regions into a tube. 500 ng of RNA (in a volume of 50 µL) was used to prepare the library using the NEBNext^®^ Ultra™ II Directional RNA Library Prep Kit for Illumina^®^ (New England Biolabs, USA) according to the manufacturer’s protocol. In adaptor ligation step, the NEBNext adaptor for Illumina (provided at 15 μM) was diluted fivefold in dilution buffer (10 mM Tris-HCI, 10 mM NaCl) (Astral Scientific, Australia). The PCR enrichment of adaptor-ligated DNA cycling conditions, denaturation, and annealing/extension cycle steps were repeated with a total of 8 cycles. Quantification and size estimation of the libraries was performed on both the Qubit 3.0 Fluorometer and 4200 TapeStation System (Agilent, USA).

2 µL of each library were pooled into a new microfuge tube and enzymatically treated with Illumina Free Adapter Blocking Reagent (Illumina, San Diego, CA). The pooled library was pre-sequenced on the MiniSeq Sequencer (2 ×150 bp paired-end reads) (Illumina, San Diego, CA) to obtain the read distribution of each sample. Each library was then re-pooled to equal molar concentrations, enzymatic treated, denatured and normalised to 2 nM. Finally, the pooled library was sequenced on the NovaSeq 6000 Sequencer (2 × 150 bp paired-end reads) (Illumina, San Diego, CA) at the Deakin University Genomics Centre.

RNA-seq reads were aligned to the GRCh38/hg38 build of the human genome using the Subread aligner. Only uniquely mapped reads were retained. Genewise counts were obtained using featureCounts. Reads overlapping exons in annotation build 38.2 of NCBI RefSeq database were included. Genes were excluded from downstream analysis if they failed to achieve a CPM value of 0.5 or greater in at least three libraries. Read counts were converted to log_2_CPMs, quantile-normalised and precision weighted with the limma voom function. Log_2_CPM values were then converted to log_2_RPKM (reads per kilo exonic bases per million mapped reads) values. A linear model was fitted to each gene. Differentially expressed genes were assessed using the limma treat function [37], which tested against a 1.5-fold expression changes of genes based on the empirical Bayes moderated *t* statistic. The differentially expressed genes were called with a FDR cutoff of 0.05.

### Gene set enrichment analysis

Gene set enrichment analysis was performed using GSEA v4.1.0 software obtained from the Broad institute (https://www.gsea-msigdb.org/gsea/index.jsp) [38]. We ran our data against the MSigDB “HALLMARKS” gene set signatures [39] and an intestinal lineage signature which was derived and modified from single cell RNAseq from murine intestine [20]. “Signal2Noise” metric used for gene ranking. Threshold for FDR was set at *q* < 0.05.

### Statistics and animal models

Values shown are mean ± SEM unless otherwise stated. Groups were compared using two-sided students *t* tests, or 1-way ANOVA (with Tukey’s correction) as stated in the Figure legends. Correlative analyses were performed using Pearson’s correlation coefficient. *P* < 0.05 considered statistically significant in all cases. For the xenograft study, *n* = 5 mice were used per isogenic cell line, with two tumours grown per mouse (left and right flank) as per previous studies [40]. For the lung metastasis study, *n* = 8 mice were used per isogenic cell line. For studies involving genetically modified mice, the number of animals per group was based on the availability of mice of the different genotypes and is stated in the figure legends. Some *Ehf* ^*KO*^ mice had to be euthanized prematurely due the development of non-tumour related pathologies such as genital abscess [16] and were therefore excluded from tumour counts. For xenograft and metastasis studies, all recipient mice were age and gender matched (male) therefore no formal randomisation was performed. Analyses were performed on Prism v5.04 (GraphPad Software).

## Supplementary information

Supplementary Table 1

Supplementary Table 2

Supplementary Table 3

Supplementary Figure 1

Supplementary Figure 2

Supplementary Figure 3

Supplementary Figure 4

Supplementary Figure 5

Supplementary Figure 6

Supplementary Figure 7

Supplementary Figure Legends

Supplementary File 1 - Full length western blots

Checklist

## Data Availability

RNA-seq and ATAC-seq data have been deposited in GEO under the following accession numbers: RNA-Seq: GSE202073, ATAC-Seq: GSE202072 and SuperSeries: GSE202074.
