# Supplementary material for: Epithelial de-differentiation triggered by co-ordinate epigenetic inactivation of the EHF and CDX1 transcription factors drives colorectal cancer progression
Source: Cell Death Differ. 2022 May 23;29(11):2288–302. doi: 10.1038/s41418-022-01016-w (PMC9613692; doi:10.1038/s41418-022-01016-w)
Supplement: Supplementary file 1 — Supplementary Table 1 [file 41418_2022_1016_MOESM1_ESM.docx]

**Table S1. List of q-RT-PCR primers used.**

| Target gene | Forward Primer Sequence | Reverse Primer Sequence |
| --- | --- | --- |
| *Actin* | CACCTTCACCGTTCCAGTTT | GATGAGATTGGCATGGCTTT |
| *EHF* | TATTGGATTTCCCACCCAGA | CTGGTGAAGGAGGTTGTTGC |
| *CDX1* | ACTGAACGGCAGGTGAAGA | GCTGTTTCTTCTTGTTCACTTTGC |
| *ELF3* | AGCGATGGTTTTCGTGACTG | GCGTGCTTGCTCTTCTTG |
| *ISX* | CAGGAAGGAAGGAAGAGCAA | TGGGTAGTGGGTAAAGTGGAA |
| *GATA6* | CAAACCAGGAAACGAAAACC | AAGAGGTGGAAGTTGGAGTCA |
| *VIL1* | AGCCAGATCACTGCTGAGGT | TGGACAGGTGTTCCTCCTTC |
| *GPA33* | TGACCTGCCAATCAAAGGAG | TAACCCGATGTGTCTGTGGA |
| *KRT20* | ACGCCAGAACAACGAATACC | ACGACCTTGCCATCCACTAC |
| *CDH17* | AGGCCAAGAACCGAGTCAAAT | GCAACCTGGAGATTGTGAGTAGA |
| *mActin* | ACTGGGACGACATGGAGAAG | GGGGTGTTGAAGGTCTCAAA |
| *mEhf* | CAGGAGTTCGACATTAGCGGA | TCTACTGTGCTACCATAGCTGG |
| *mCdx1* | GGCTCCTTGGCCCGGCGG | CCGAGCTGGCTGCTAACC |
| *mVil1* | TGGAGGAGGAGGATGTGTTC | GGGTCTCAAGGTCTCGGTTT |
| *mGpa33* | CTACATCTGCACCTCCAGCA | GCAGCAGCAGCAGTAGACAA |
| *mCdh17* | GACAACCGACCCACGTTTCT | CCCTGTTTTGCTGTCGATTTG |
| *mLgr5* | GGACCAGATGCGATACCGC | CAGAGGCGATGTAGGAGACTG |
| *VIL1 P1* | gaccctacctggacagtgct | gccaggctggacttgaact |
| *VIL1 P2* | aacatggtgaaaccctgtctc | tgagcctcactctgtcattca |
| *VIL1 P3* | TTGCCACAATTCCCTGAGAT | cccgagaaaagcagaggac |
| *VIL1 P4* | ggtctccctgacctcactcc | gaggtcccaggagtgtcaga |
| *VIL1 P5* | cgggtgtccattccctct | acttgagttgcggctctgtt |
