# Supplementary material for: Epithelial de-differentiation triggered by co-ordinate epigenetic inactivation of the EHF and CDX1 transcription factors drives colorectal cancer progression
Source: Cell Death Differ. 2022 May 23;29(11):2288–302. doi: 10.1038/s41418-022-01016-w (PMC9613692; doi:10.1038/s41418-022-01016-w)
Supplement: Supplementary file 2 — Supplementary Table 2 [file 41418_2022_1016_MOESM2_ESM.docx]

**Table S2. List of siRNAs used.**

| siRNA Gene | Target | Sequence of siRNA |
| --- | --- | --- |
| ON-Target-Plus EHF#1 | ORF | AGACAAGAACCCAGGAUUA |
| ON-Target-Plus EHF#2 | ORF | GACGAGAACUAUUUAUAUG |
| ON-Target-Plus CDX1#1 | ORF | GGACAAGGAUUCGCCCGUG |
| ON-Target-Plus CDX1#2 | ORF | GUAAGACUCGGACCAACCA |
| ON-Target-Plus EHF | Pooled | AGACAAGAACCCAGGAUUA  GACGAGAACUAUUUAUAUG  GAGAGGGACUCACUUAUGG  GGAAGGAGGUGGUGUAAUG |
| ON-Target-Plus ELF3 | Pooled | GAACUGAGGGUUGGAACUA  GGAGCUGCGUCUGGUCUUU  GCCAUUGACUUCUCACGAU  GAACAAGUACGACGCAAGC |
| siGenome ISX | Pooled | CAUCUGUGCUACUUCAACA  CCAUUGAGGCGAUCCUAAA  GUCCAUCGGCUCAAGAUCA  CCAGCGCACCCAUGGGAAA |
| siGenome GATA6 | Pooled | UCAAAGACUUGCUCUGGUA  GAACAGCGAGCUCAAGUAU  CAAGAUGGGCUCUACAUAG  GCAGAAACGCCGAGGGUGA |
| siGenome CDX1 | Pooled | GGACAAGGAUUCGCCCGUG  GUAAGACUCGGACCAAGGA  CAUUACAGCCGUUACAUCA  CUCCAAUGCCUGUGAAAGA |
| Non-Targeting ON-Target-Plus | Pooled | UGGUUUACAUGUCGACUAA  GGUUUACAUGUUUUCUGA  UGGUUUACAUGUUUUCCUA  UGGUUUACAUGUUGUGUGA |
