# Supplementary material for: Epithelial de-differentiation triggered by co-ordinate epigenetic inactivation of the EHF and CDX1 transcription factors drives colorectal cancer progression
Source: Cell Death Differ. 2022 May 23;29(11):2288–302. doi: 10.1038/s41418-022-01016-w (PMC9613692; doi:10.1038/s41418-022-01016-w)
Supplement: Supplementary file 3 — Supplementary Table 3 [file 41418_2022_1016_MOESM3_ESM.docx]

**Table S3. List of significantly enriched hallmark genesets in HCT116^EHF+CDX1^ versus HCT116^EV^ cells.**

| GS DETAILS | NES | Nominal p-value | FDR q-value |
| --- | --- | --- | --- |
| HALLMARK_INTERFERON_ALPHA_RESPONSE | 1.87 | 0.000 | 0.000 |
| HALLMARK_INTERFERON_GAMMA_RESPONSE | 1.71 | 0.000 | 0.000 |
| HALLMARK_E2F_TARGETS | 1.54 | 0.000 | 0.003 |
| HALLMARK_IL6_JAK_STAT3_SIGNALING | 1.53 | 0.000 | 0.003 |
| HALLMARK_G2M_CHECKPOINT | 1.53 | 0.000 | 0.002 |
| HALLMARK_COMPLEMENT | 1.51 | 0.000 | 0.003 |
| HALLMARK_INFLAMMATORY_RESPONSE | 1.51 | 0.000 | 0.002 |
| HALLMARK_KRAS_SIGNALING_UP | 1.50 | 0.000 | 0.002 |
| HALLMARK_EPITHELIAL_MESENCHYMAL_TRANSITION | 1.47 | 0.000 | 0.004 |
| HALLMARK_HEDGEHOG_SIGNALING | 1.45 | 0.004 | 0.005 |
| HALLMARK_MYC_TARGETS_V1 | 1.45 | 0.000 | 0.005 |
| HALLMARK_XENOBIOTIC_METABOLISM | 1.41 | 0.000 | 0.009 |
| HALLMARK_COAGULATION | 1.40 | 0.000 | 0.011 |
| HALLMARK_HYPOXIA | 1.38 | 0.000 | 0.017 |
| HALLMARK_OXIDATIVE_PHOSPHORYLATION | 1.38 | 0.000 | 0.016 |
| HALLMARK_REACTIVE_OXYGEN_SPECIES_PATHWAY | 1.37 | 0.028 | 0.015 |
| HALLMARK_PI3K_AKT_MTOR_SIGNALING | 1.35 | 0.006 | 0.022 |
| HALLMARK_MTORC1_SIGNALING | 1.35 | 0.000 | 0.021 |
| HALLMARK_IL2_STAT5_SIGNALING | 1.33 | 0.001 | 0.026 |
| HALLMARK_ALLOGRAFT_REJECTION | 1.33 | 0.001 | 0.025 |
| HALLMARK_PROTEIN_SECRETION | 1.31 | 0.017 | 0.032 |
| HALLMARK_ANDROGEN_RESPONSE | 1.29 | 0.030 | 0.040 |
| HALLMARK_FATTY_ACID_METABOLISM | 1.29 | 0.011 | 0.041 |
| HALLMARK__MITOTIC_SPINDLE | 1.28 | 0.004 | 0.042 |
| HALLMARK_TNFA_SIGNALING_VIA_NFKB | 1.28 | 0.005 | 0.040 |
| HALLMARK_UNFOLDED_PROTEIN_RESPONSE | 1.26 | 0.034 | 0.050 |
