# Supplementary figures and images for: Epithelial de-differentiation triggered by co-ordinate epigenetic inactivation of the EHF and CDX1 transcription factors drives colorectal cancer progression
Source: Cell Death Differ. 2022 May 23;29(11):2288–302. doi: 10.1038/s41418-022-01016-w (PMC9613692; doi:10.1038/s41418-022-01016-w)

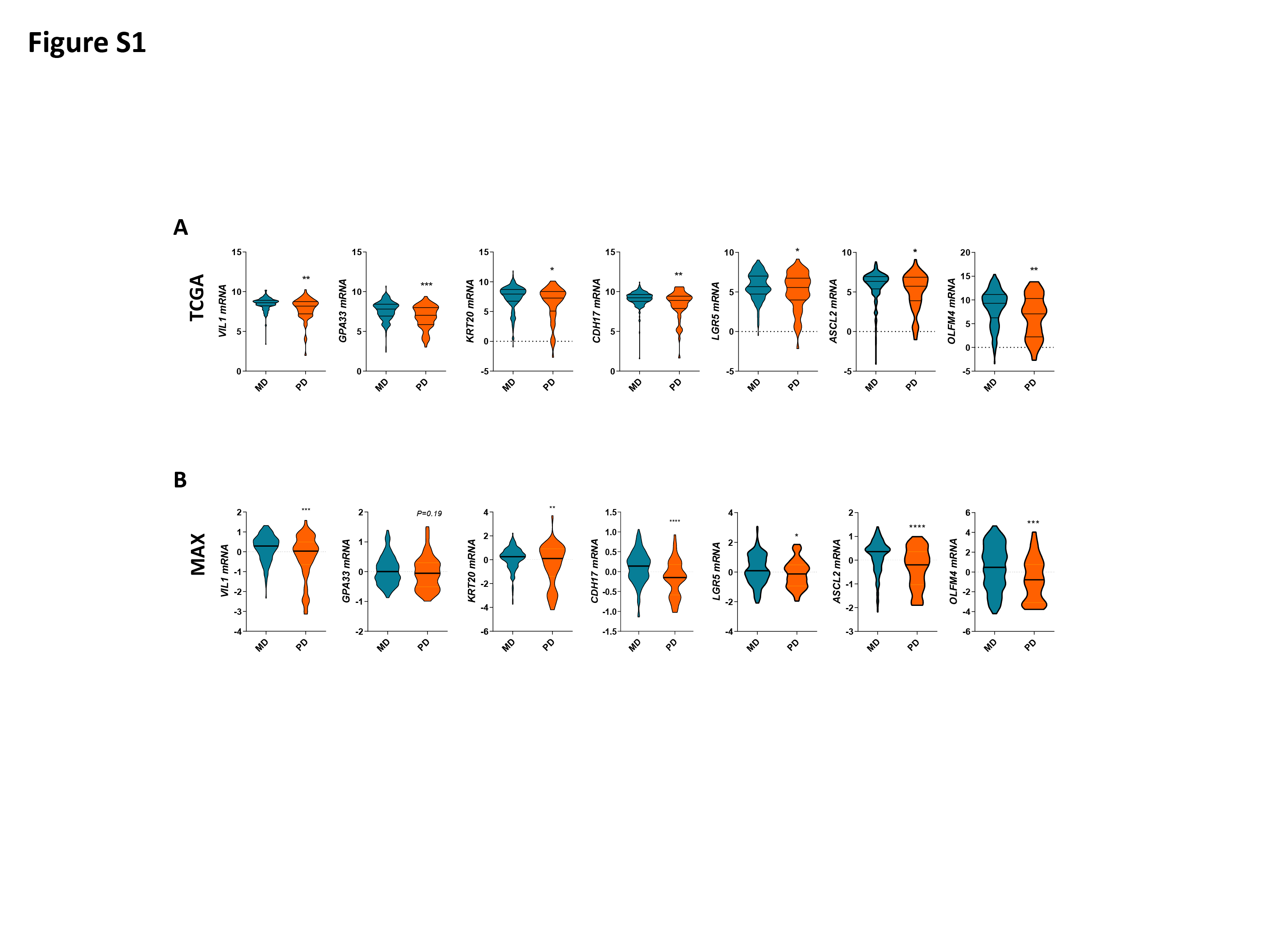

Supplement: Supplementary file 4 — Supplementary Figure 1 [file 41418_2022_1016_MOESM4_ESM.png]

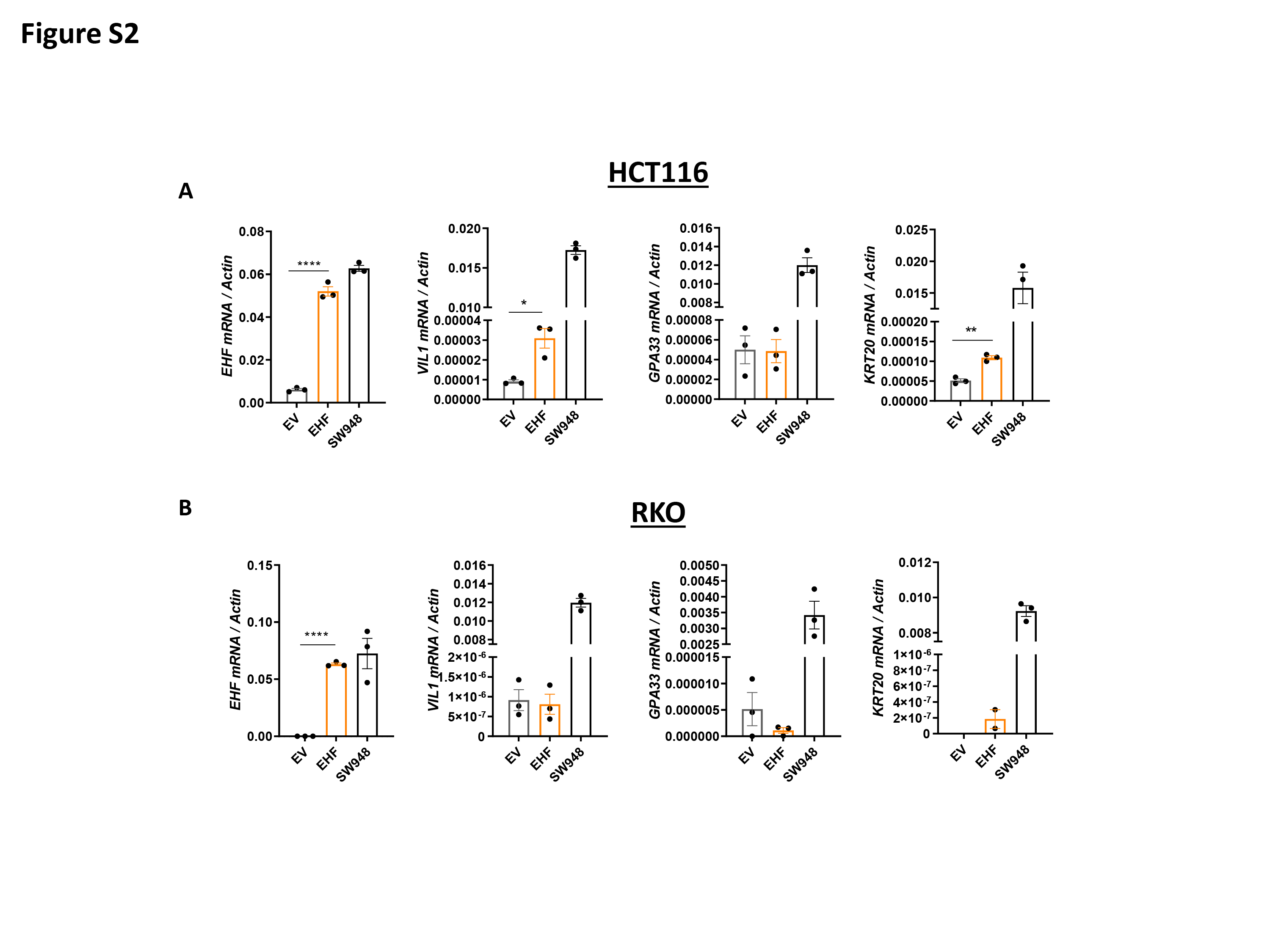

Supplement: Supplementary file 5 — Supplementary Figure 2 [file 41418_2022_1016_MOESM5_ESM.png]

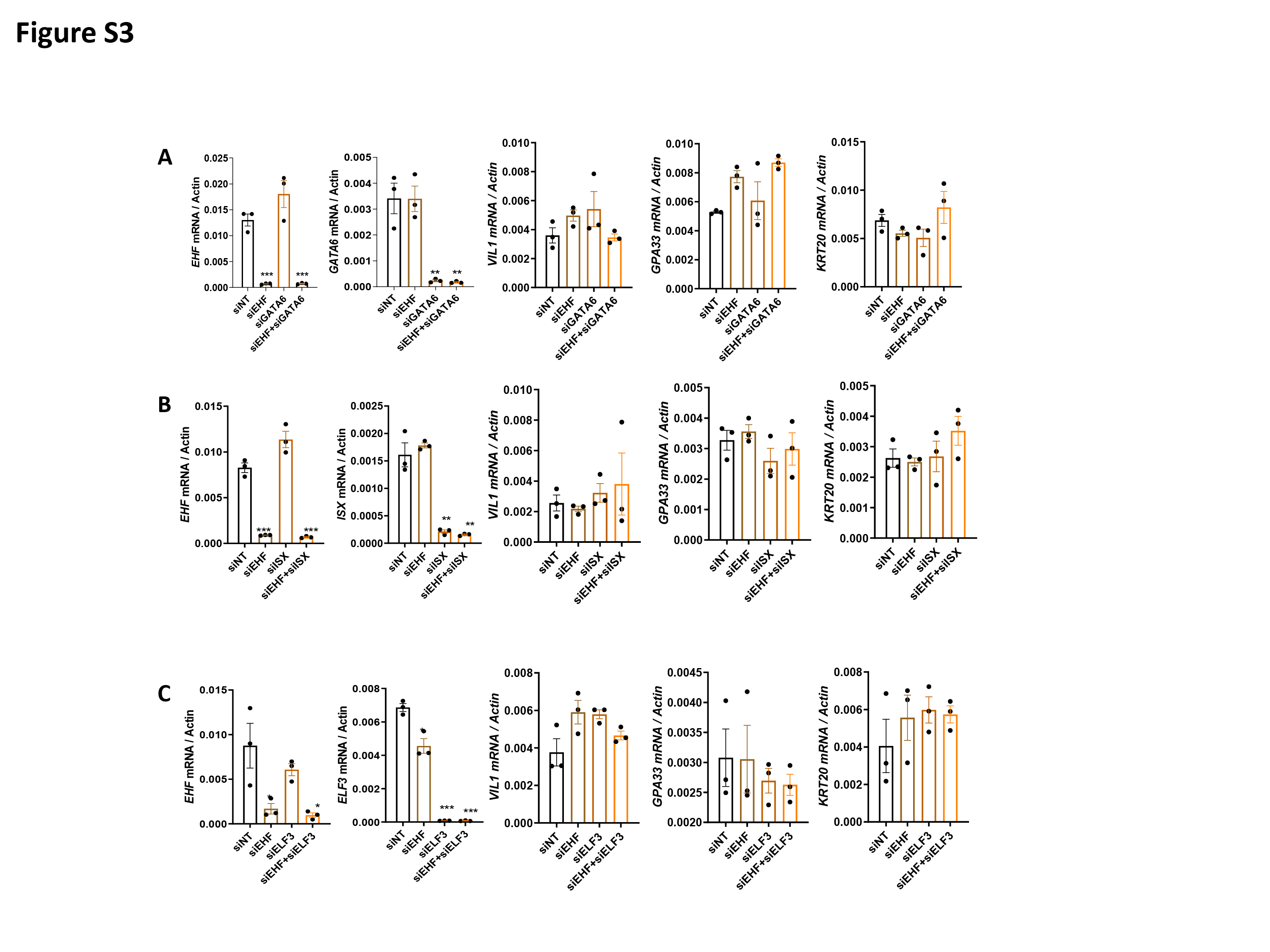

Supplement: Supplementary file 6 — Supplementary Figure 3 [file 41418_2022_1016_MOESM6_ESM.png]

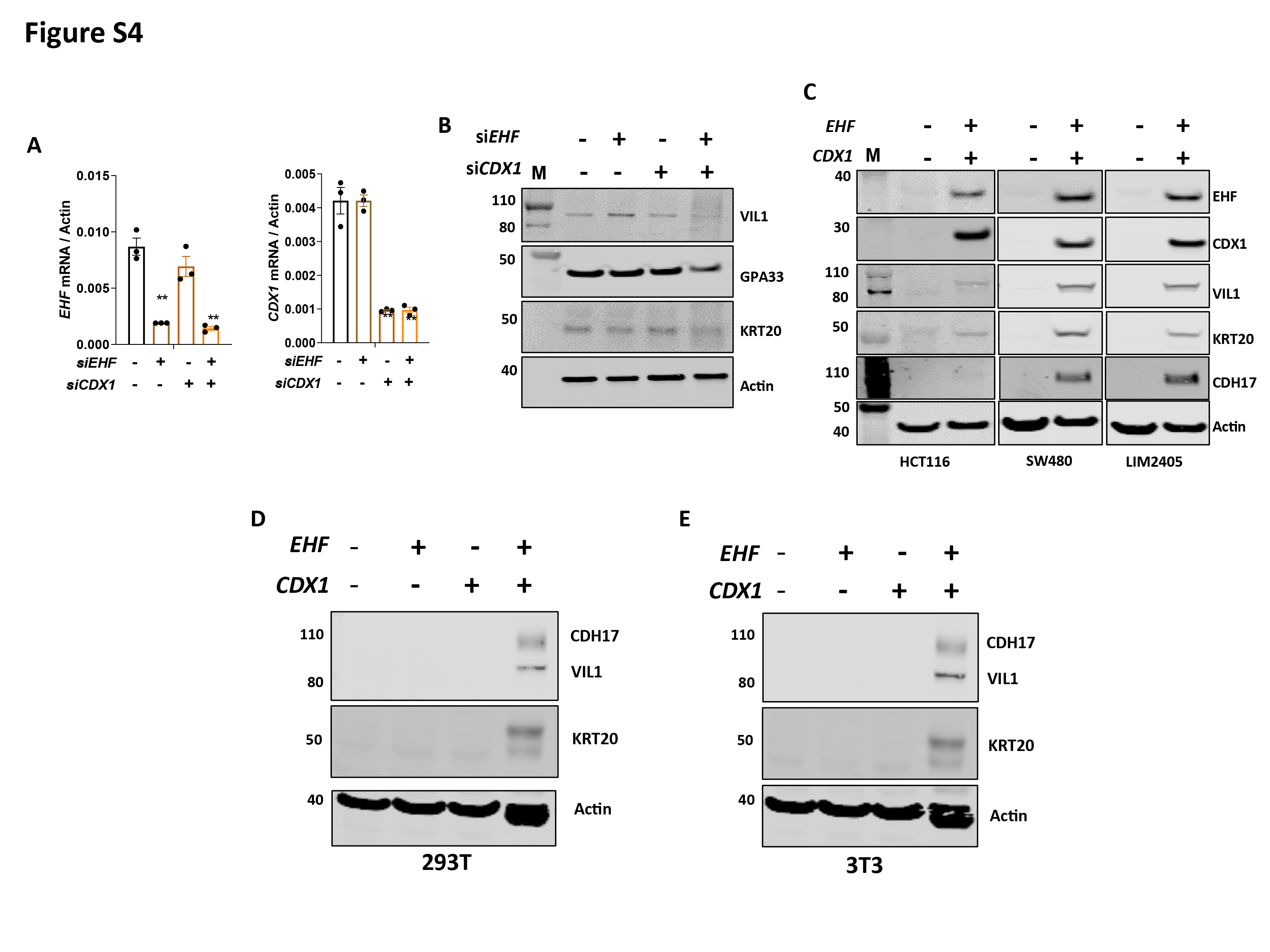

Supplement: Supplementary file 7 — Supplementary Figure 4 [file 41418_2022_1016_MOESM7_ESM.png]

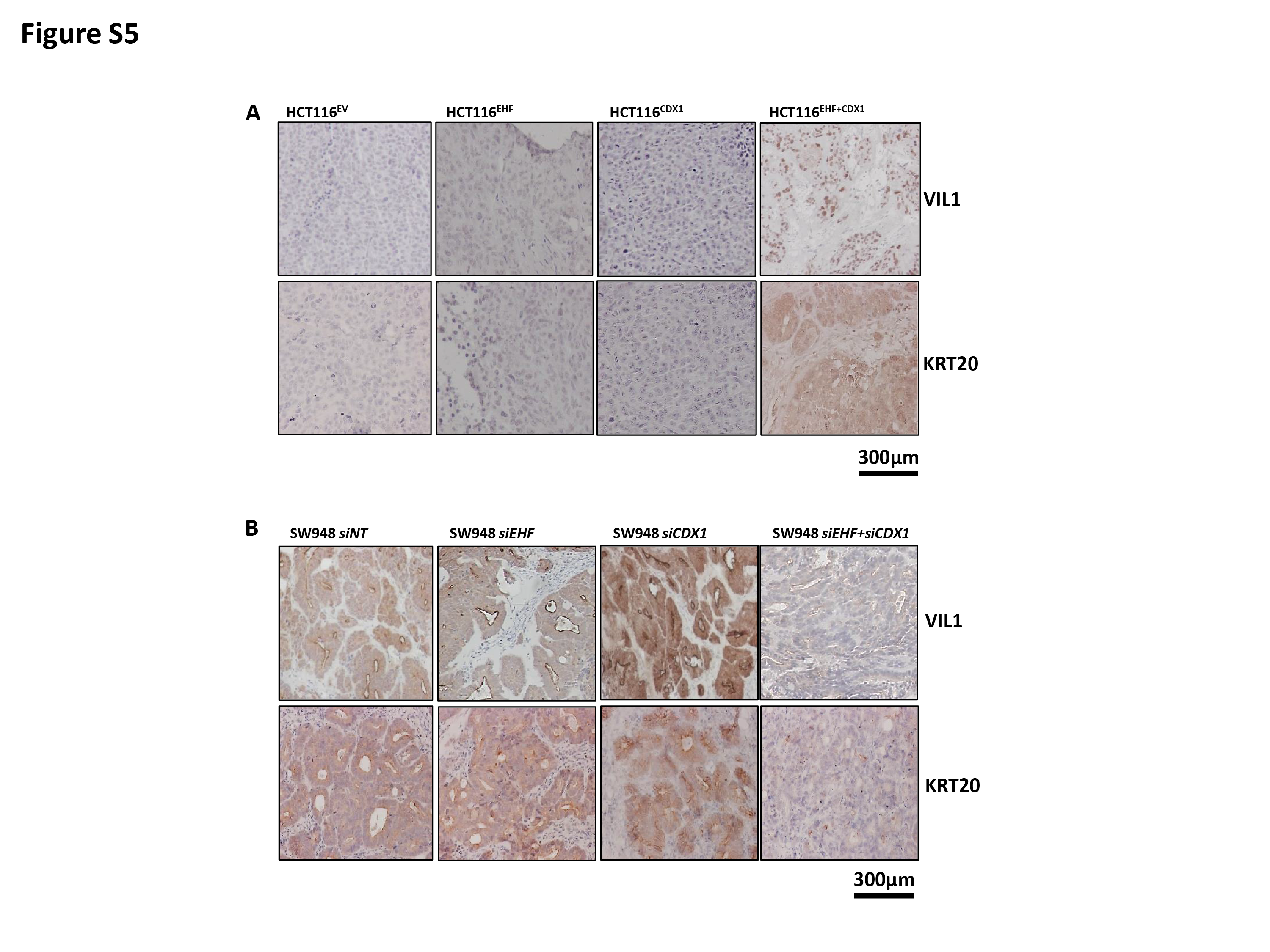

Supplement: Supplementary file 8 — Supplementary Figure 5 [file 41418_2022_1016_MOESM8_ESM.png]

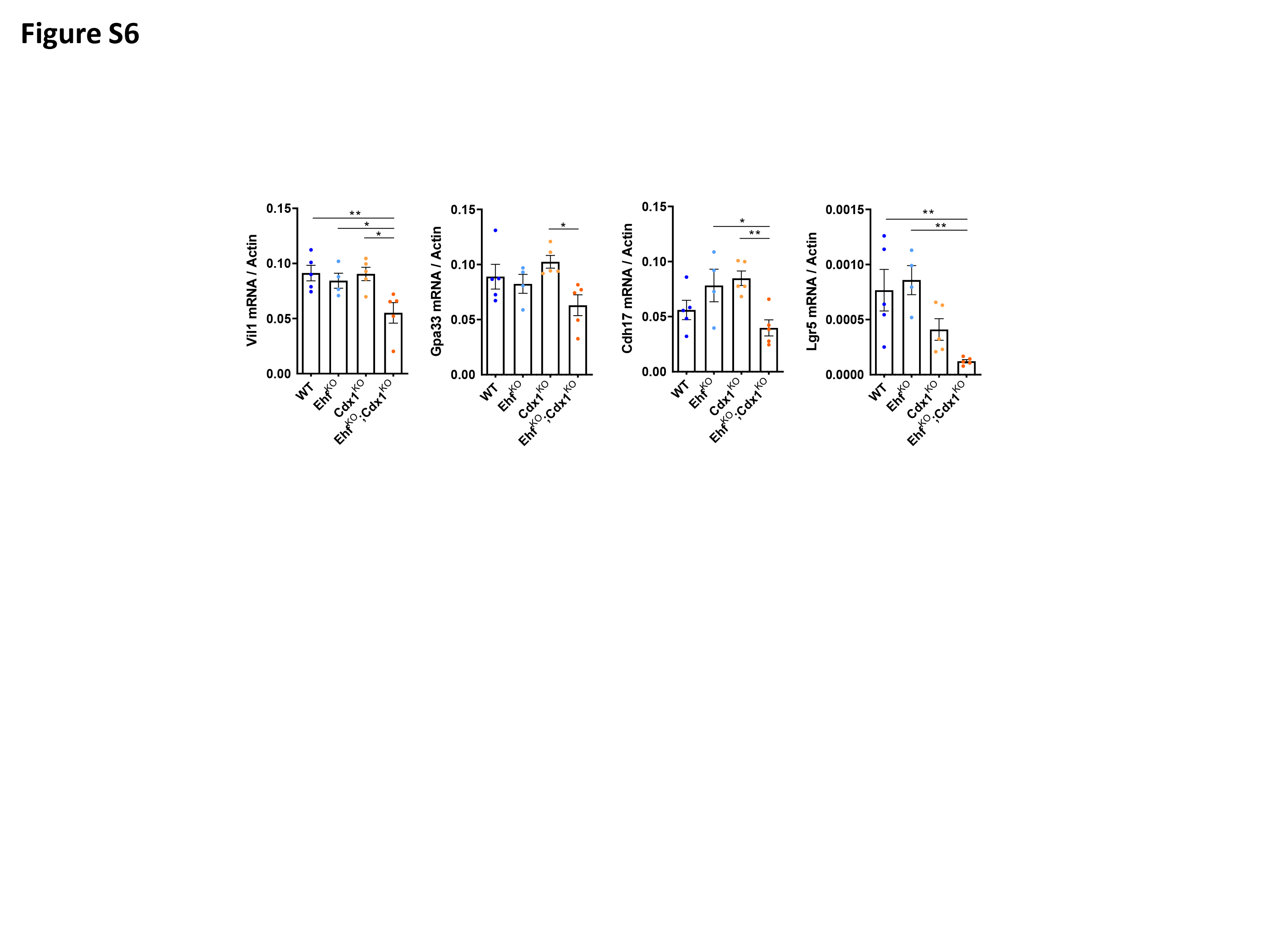

Supplement: Supplementary file 9 — Supplementary Figure 6 [file 41418_2022_1016_MOESM9_ESM.png]

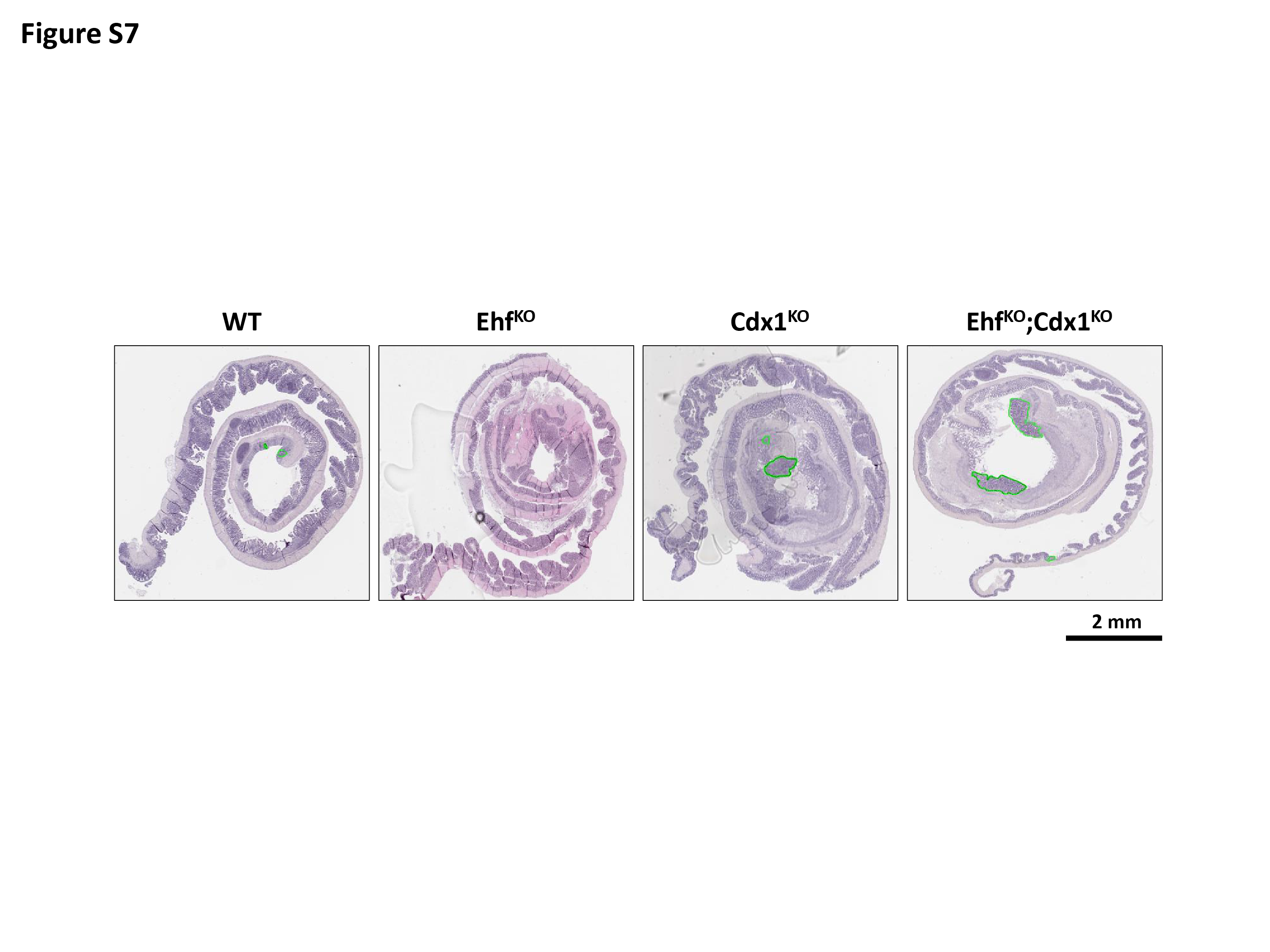

Supplement: Supplementary file 10 — Supplementary Figure 7 [file 41418_2022_1016_MOESM10_ESM.png]
