# Supplementary material for: Epithelial de-differentiation triggered by co-ordinate epigenetic inactivation of the EHF and CDX1 transcription factors drives colorectal cancer progression
Source: Cell Death Differ. 2022 May 23;29(11):2288–302. doi: 10.1038/s41418-022-01016-w (PMC9613692; doi:10.1038/s41418-022-01016-w)
Supplement: Supplementary file 11 — Supplementary Figure Legends [file 41418_2022_1016_MOESM11_ESM.docx]

**Supplementary Figure legends**

**Figure S1. Differentiation marker expression is downregulated in poorly-differentiated CRCs.**

**(A-B)** Violin plots of the mRNA expression levels of colonic differentiation markers and intestinal stem cells markers determined in n=181 moderately differentiated (MD) and n=52 poorly differentiated (PD) primary colorectal cancers. Data were derived from **(A)** the COAD cohort profiled by the TCGA (The Cancer Genome Atlas) or **(B)** the phase III MAX clinical trial cohort [44]. Data shown are mean±SEM. **p*<0.05, ***p*<0.01, ****p*<0.001, *****p*<0.0001, Student’s t-test.

**Figure S2. Altering EHF expression alone does not impact differentiation of CRC cells**.

**(A-B)** Poorly-differentiated **(A)** HCT116 and **(B)** RKO CRC cells were stably transfected with *EHF* expression construct and mRNA expression of differentiation markers determined by q-RT-PCR. Moderately-differentiated SW948 CRC cells was used as control. q-RT-PCR data shown are mean±SEM from a representative experiment performed in triplicate. **p*<0.05, *****p*<0.0001, Student’s t-test.

**Figure S3. Combinatorial knockdown of EHF with GATA6, ISX and ELF3.**

**(A-C)** Moderately-differentiated SW948 CRC cells were transiently transfected with siRNAs targeting *EHF* and **(A)** *GATA6*, **(B)** *ISX* or **(C)** *ELF3* alone and in combination and expression of differentiation markers VIL1, GPA33 and KRT20 determined by q-RT-PCR. Values shown are mean±SEM from a representative experiment performed in triplicate. Data shown are mean ± SEM. **p*<0.05, ***p*<0.01, ****p*<0.001. One-way ANOVA with Tukey’s post hoc test.

**Figure S4. *EHF* and *CDX1* cooperatively regulate differentiation of CRC cells.**

**(A-B)** Moderately-differentiated SW403 CRC cells were transiently transfected with siRNAs targeting EHF and CDX1 alone and in combination. Expression of EHF and CDX1 were determined by **(A)** q-RT-PCR and differentiation markers VIL1, GPA33 and KRT20 determined by **(B)** western blot. **(C-E)** Transient re-expression of EHF and CDX1 in **(C)** poorly-differentiated CRC cell lines HCT116, SW480 and LIM2405, and non-colonic epithelial cells **(D)** 293T and **(E)** 3T3 on differentiation marker (VIL1, KRT20 and CDH17) and transcription factor (EHF and CDX1) protein expression. Cell lines were transduced for 72 hours before harvesting for subsequent experiments by western blot. Values shown in panel A is mean±SEM from a representative experiment performed in triplicate. **p*<0.05, ***p*<0.01, ****p*<0.001. One-way ANOVA with Tukey’s post hoc test.

**Figure S5. Immunohistochemical analysis of VIL1 and KRT20 in SW498 and HCT116 cell line xenografts.**

**(A-B)** Immunohistochemical analysis of the differentiation markers VIL1 and KRT20 in xenografts of **(A)** HCT116 cells stably transfected with *EHF* and *CDX1* alone and in combination and **(B)** SW948 cells transiently transfected with siRNAs targeting EHF and CDX1 alone and in combination.

**Figure S6. Characterization of 6-week-old *Ehf^KO^; Cdx1^KO^* mice.**

Colonic epithelial cells were isolated from 6 week old *WT*, *Ehf^KO^*, Cdx*1^KO^* and *Ehf^KO^;CDX1^KO^* mice and mRNA expression determined by q-RT-PCR of markers of enteroctyes (*Vil1, Gpa33* and *Cdh17*) and intestinal stem cell (*Lgr5*). Values shown are mean±SEM of n=5 mice from a representative experiment performed in triplicate. **p*<0.05, ***p*<0.01. One-way ANOVA with Tukey’s post hoc test.

**Figure S7. Immunohistochemical analysis of Ehf^ko^; Cdx1^ko^ mice post AOM/DSS.**

Representative images of H&E stained colonic Swiss rolls of *WT* (n=7), *Ehf^KO^* (n=4), *Cdx1^KO^* (n=9) and *Ehf^KO^;Cdx1^KO^* (n=8) mice 37-43 day post AOM injection. Tumour burden (green) was determined microscopically performed using the Aperio ImageScope software.
