# Supplementary material for: Epithelial de-differentiation triggered by co-ordinate epigenetic inactivation of the EHF and CDX1 transcription factors drives colorectal cancer progression
Source: Cell Death Differ. 2022 May 23;29(11):2288–302. doi: 10.1038/s41418-022-01016-w (PMC9613692; doi:10.1038/s41418-022-01016-w)
Supplement: Supplementary file 12 — Supplementary File 1 - Full length western blots [file 41418_2022_1016_MOESM12_ESM.pdf]

Supplementary File 1: Full length western blots

Fig 1E

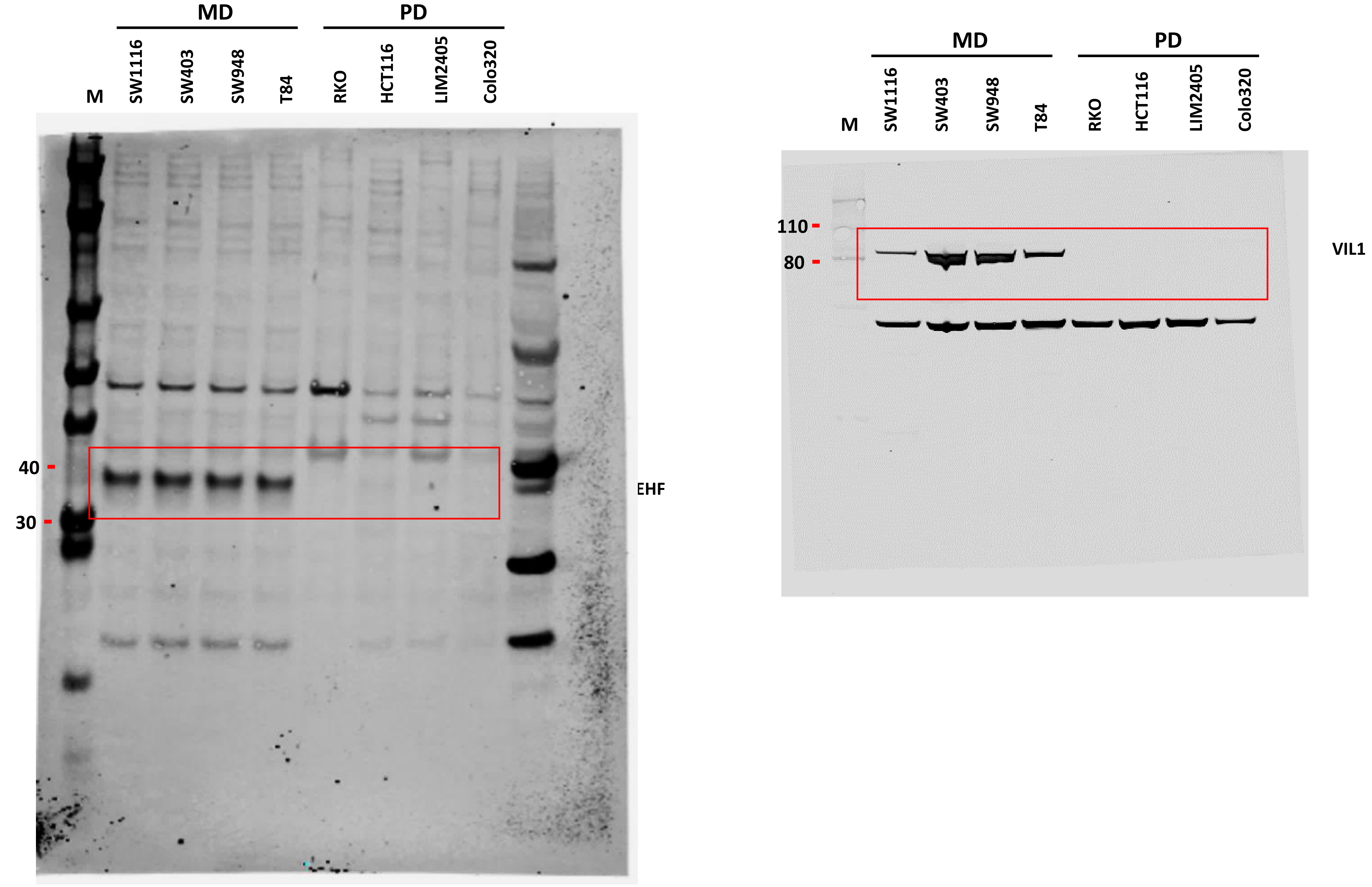
MD
PD
M
SW1116
SW403
SW948
T84
RKO
HCT116
LIM2405
Colo320
40
30
EHF
MD
PD
M
SW1116
SW403
SW948
T84
RKO
HCT116
LIM2405
Colo320
110
80
VIL1

Fig 1E

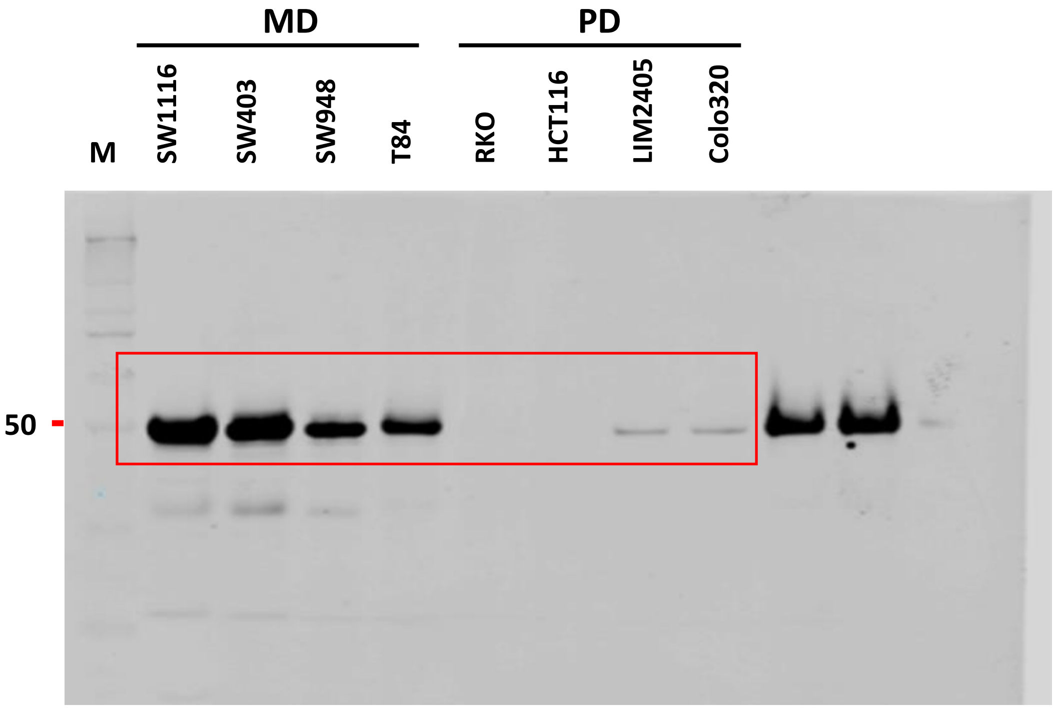

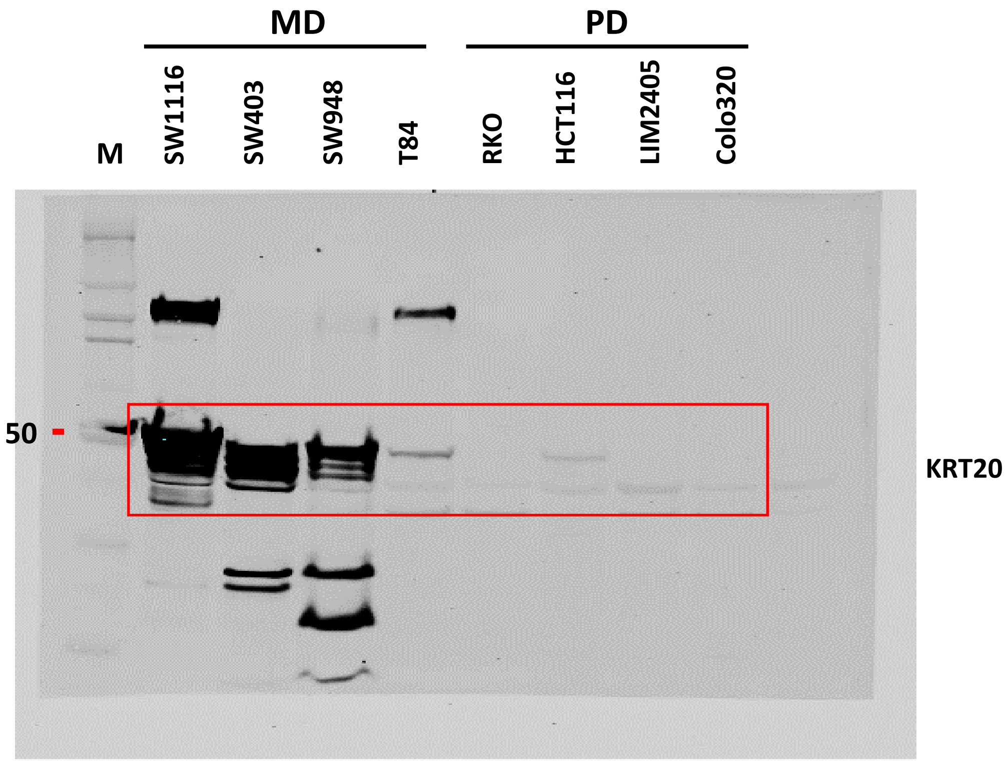

* This image shown represents the full western blot. Edges not present due to the scanned image border being smaller than the western membrane.

Fig 1E

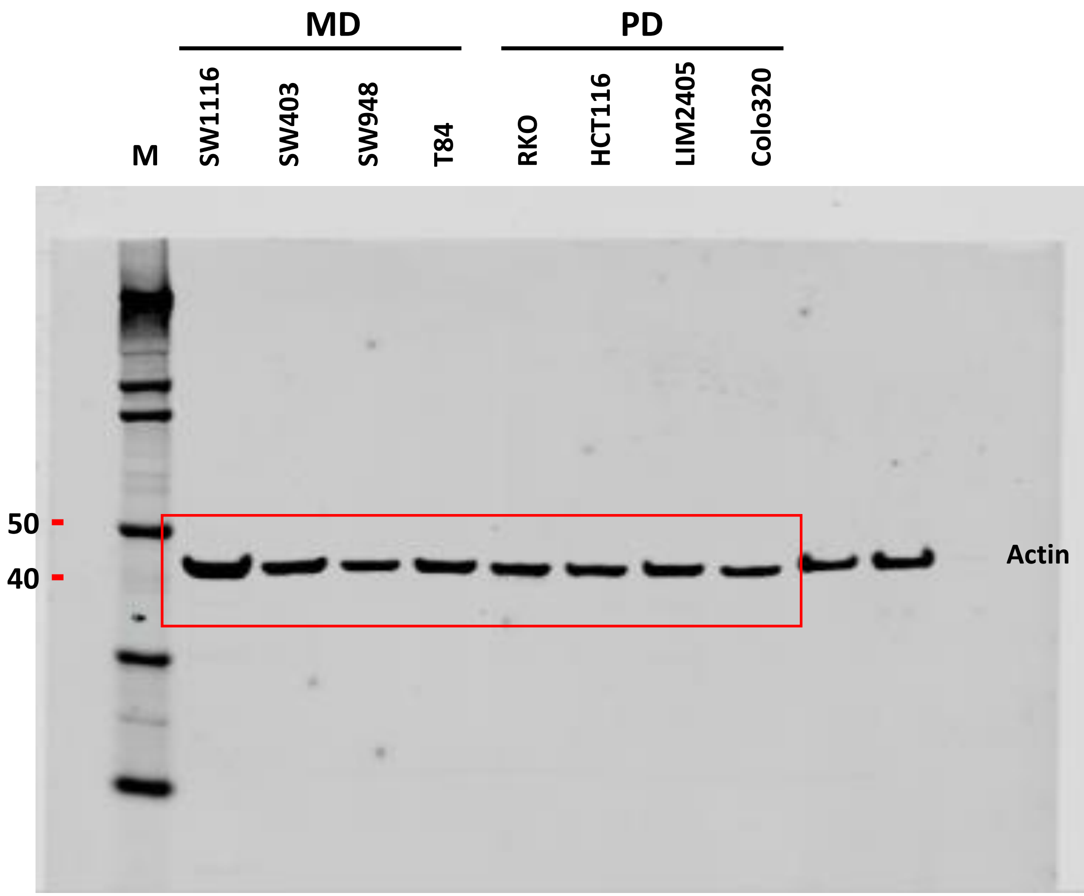
MD
PD
M
SW1116
SW403
SW948
T84
RKO
HCT116
LIM2405
Colo320
50
40
Actin

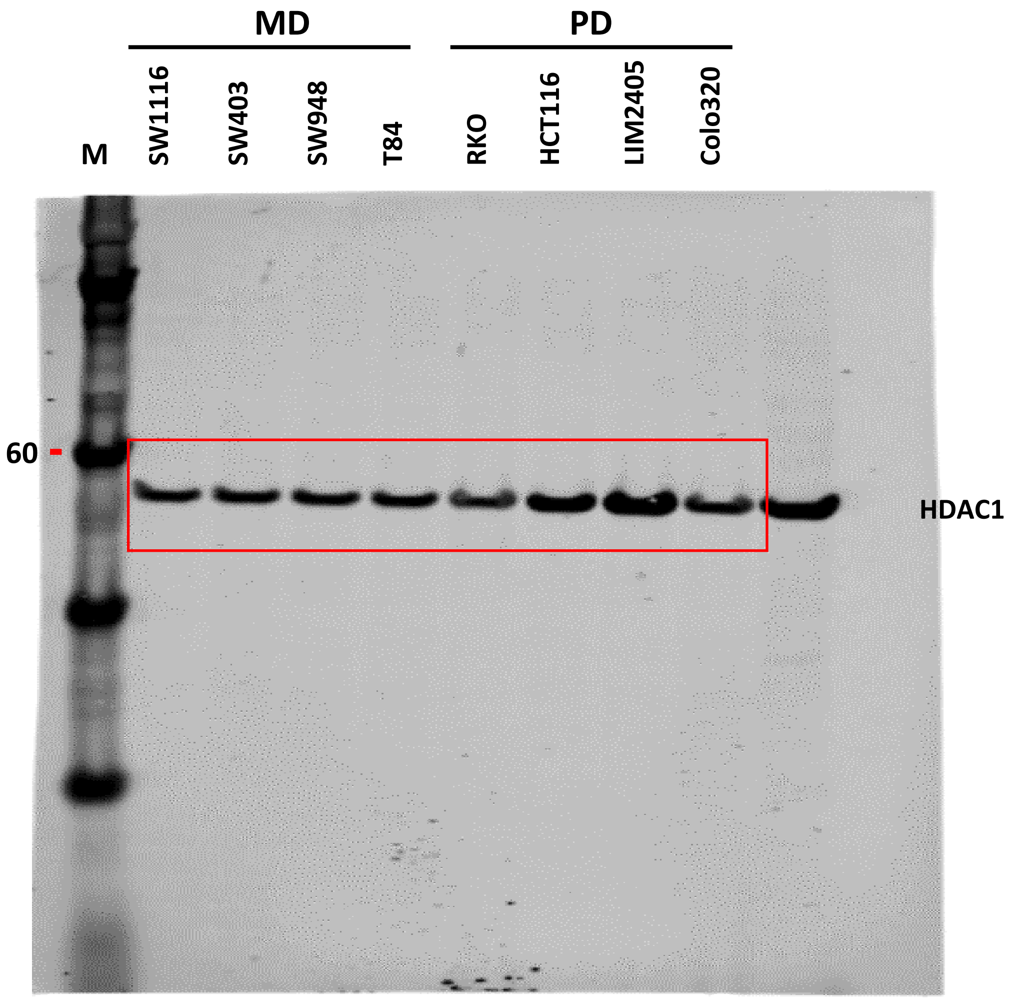
MD
PD
M
SW1116
SW403
SW948
T84
RKO
HCT116
LIM2405
Colo320
60
HDAC1

Fig 2B

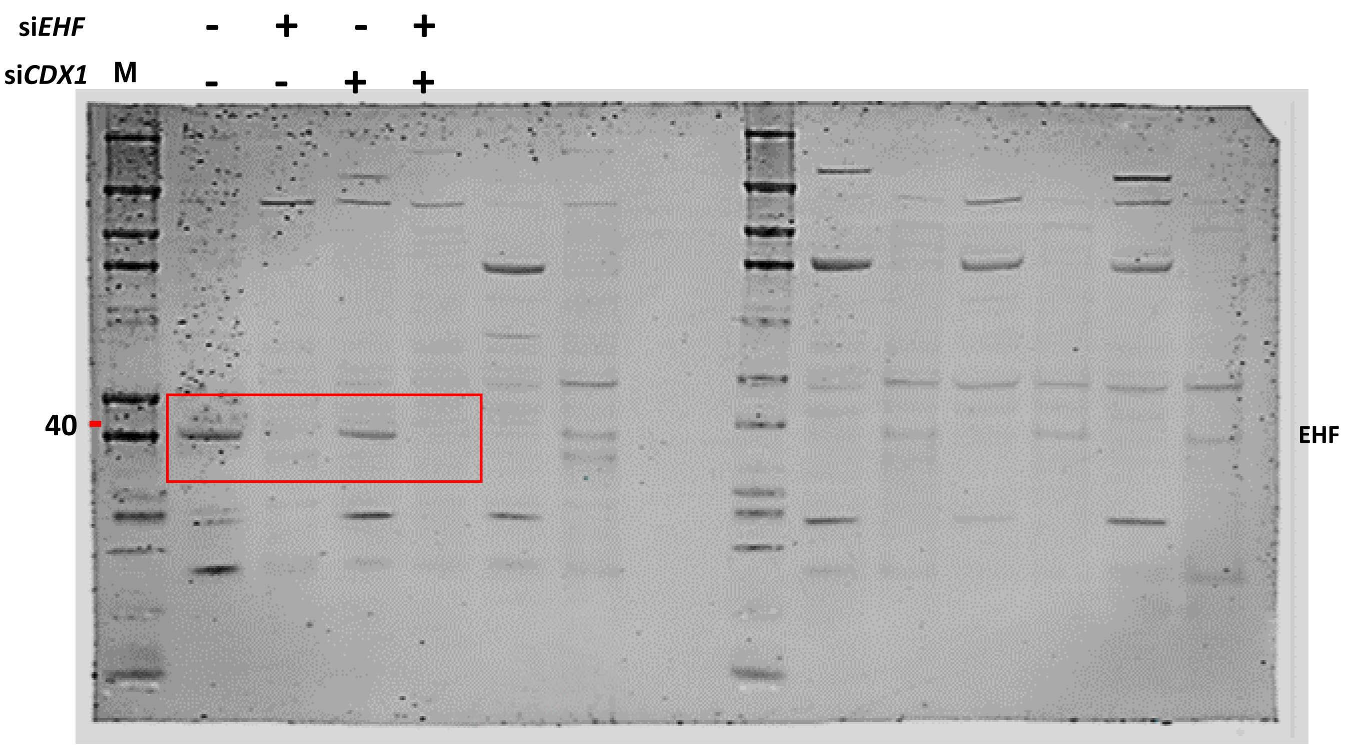

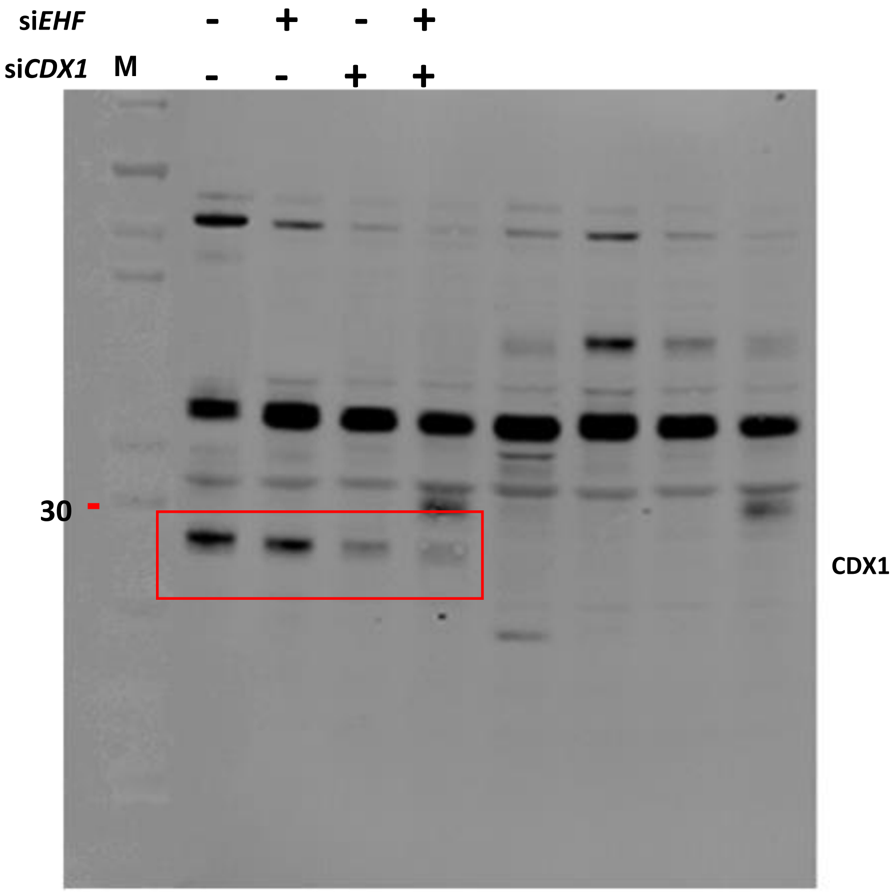

* This image shown represents the full western blot. Edges were not present due to the scanned image border being smaller than the western membrane.

Fig 2B

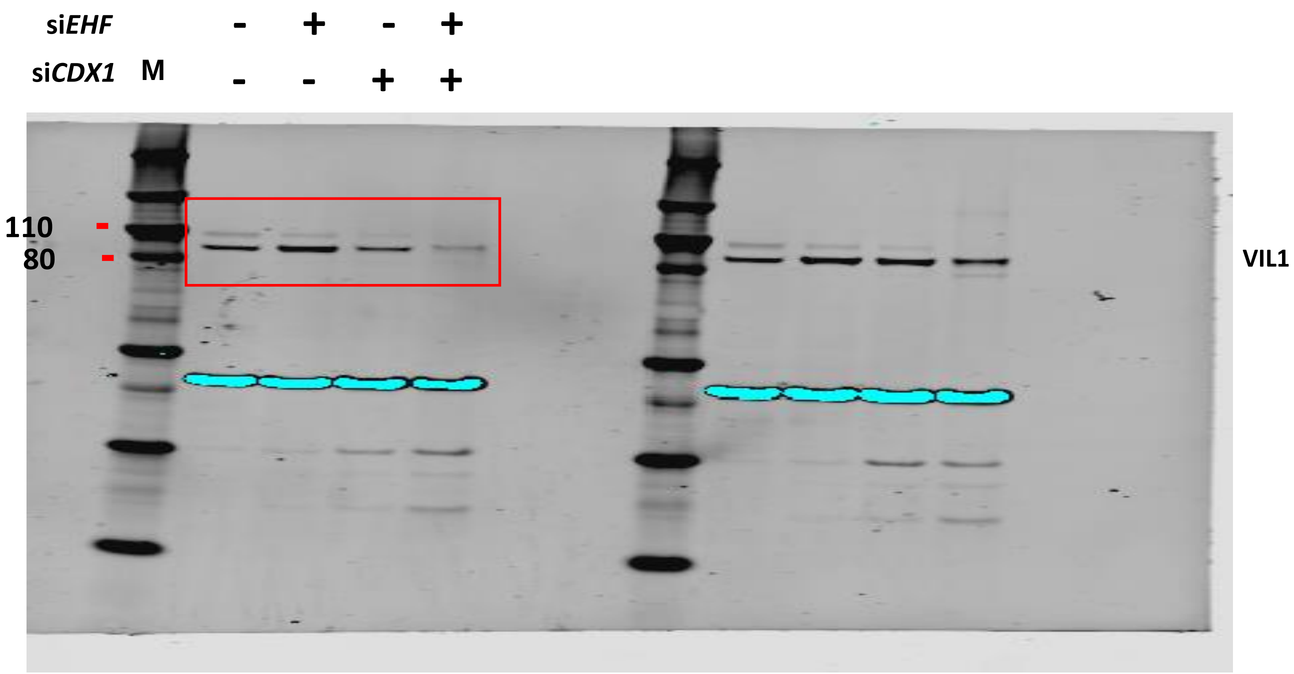

Fig 2B

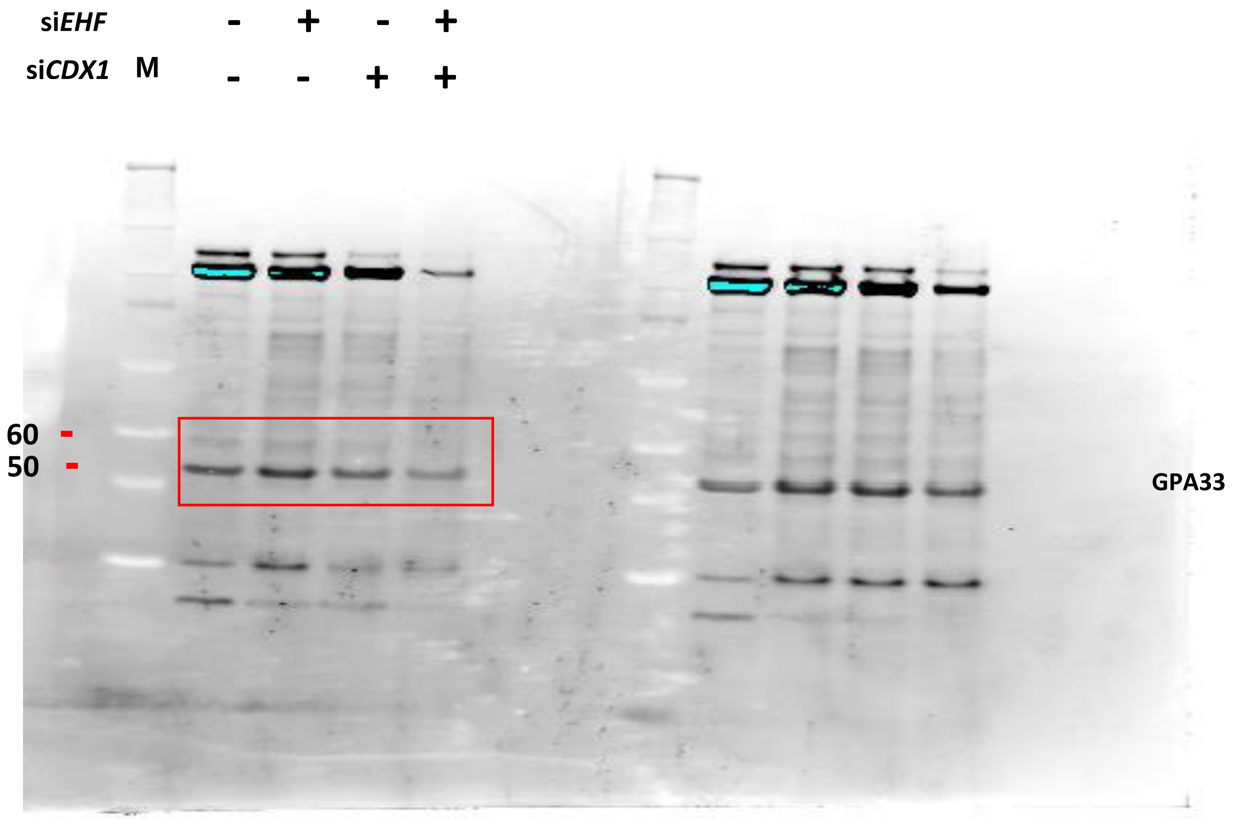

Fig 2B

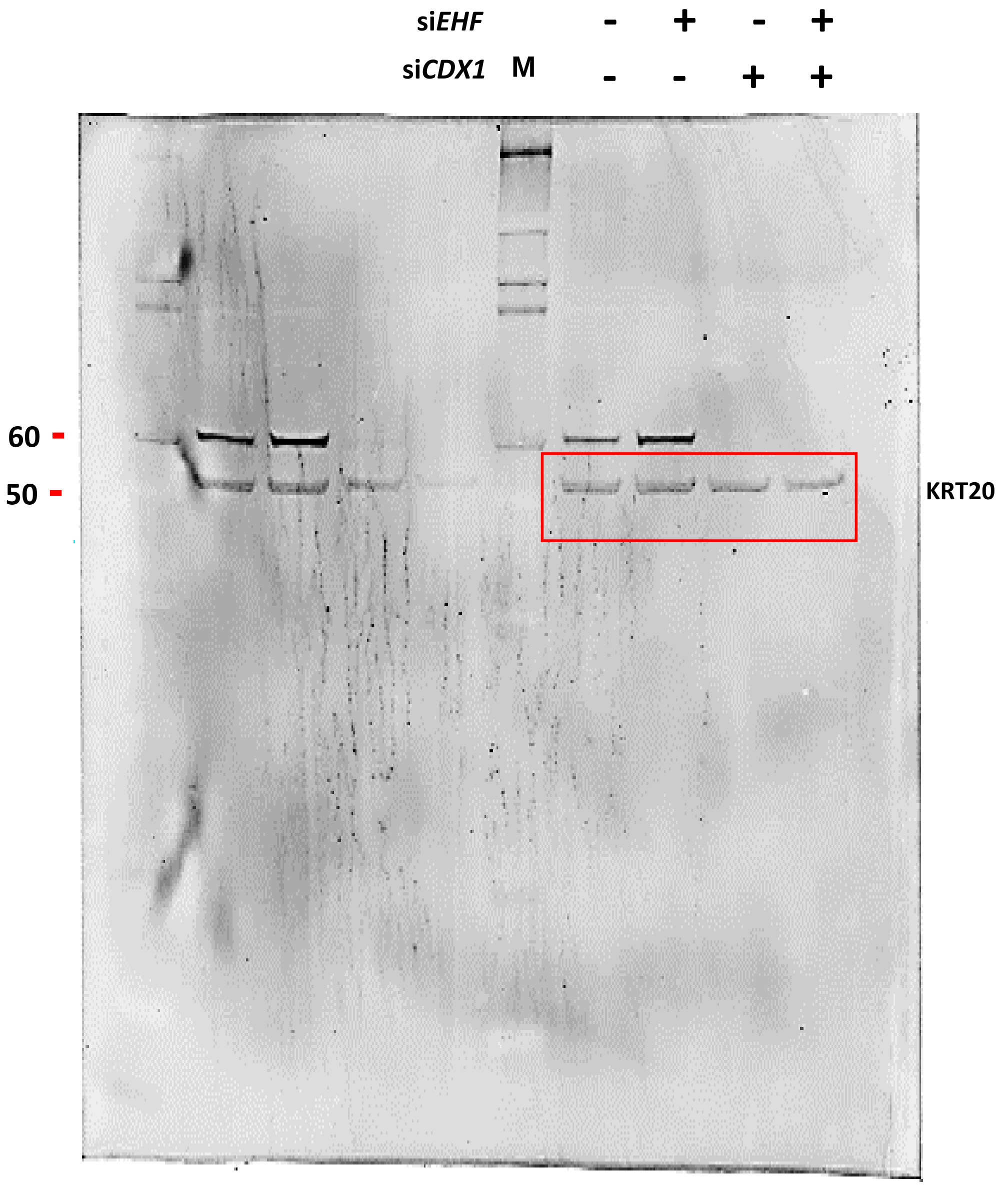

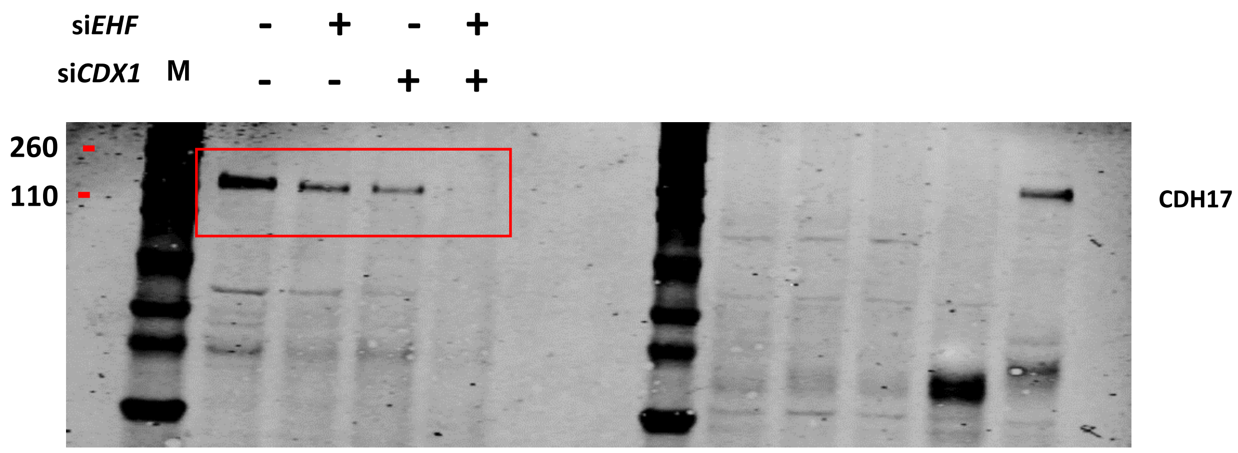

* This image shown represents the full western blot. Edges were not present due to the scanned image border being smaller than the western membrane.

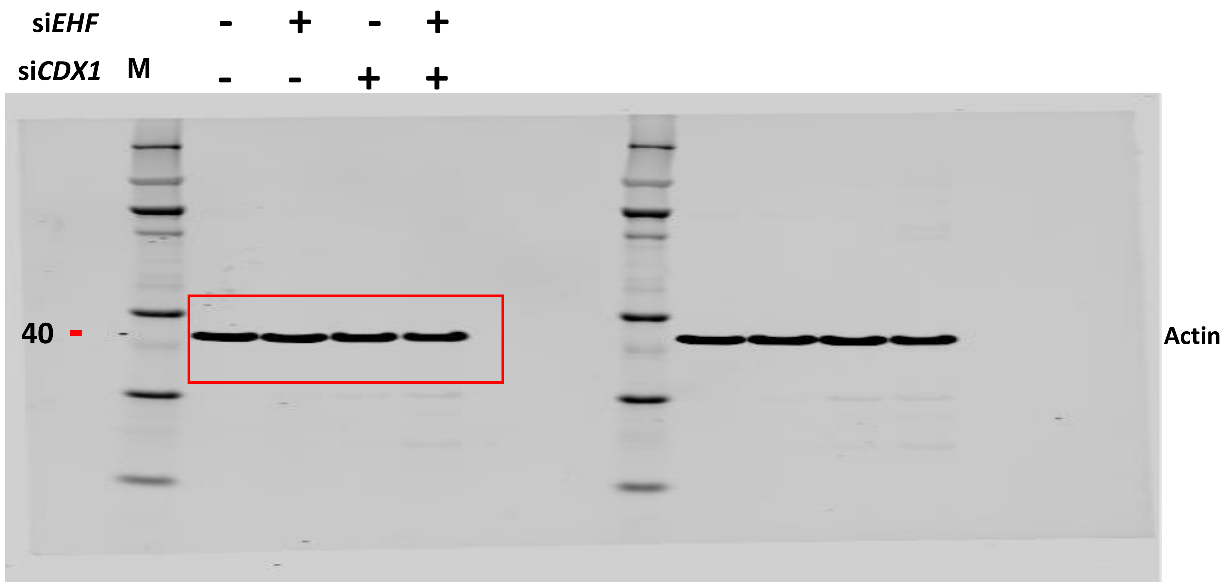

Fig 2D

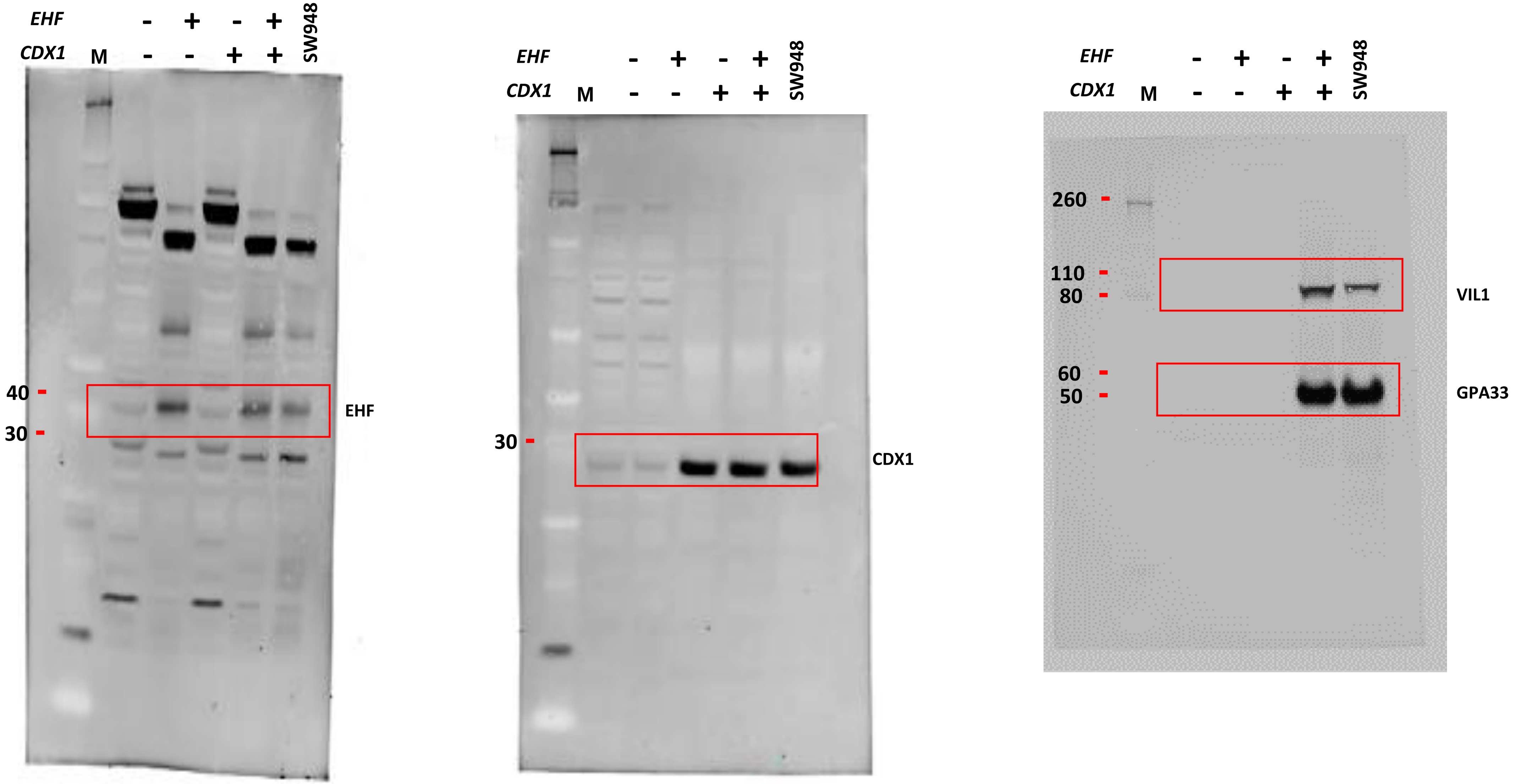

EHF
CDX1
M
SW948
40
30
EHF
EHF
CDX1
M
SW948
30
CDX1
EHF
CDX1
M
SW948
260
110
80
60
50
VIL1
GPA33

Fig 2D

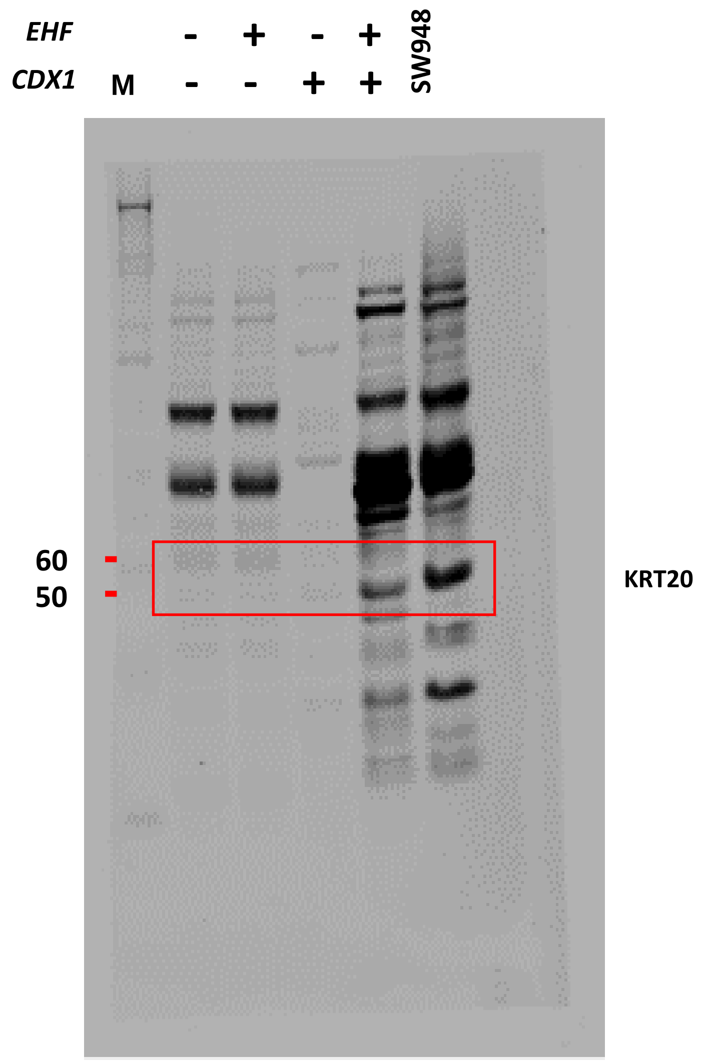

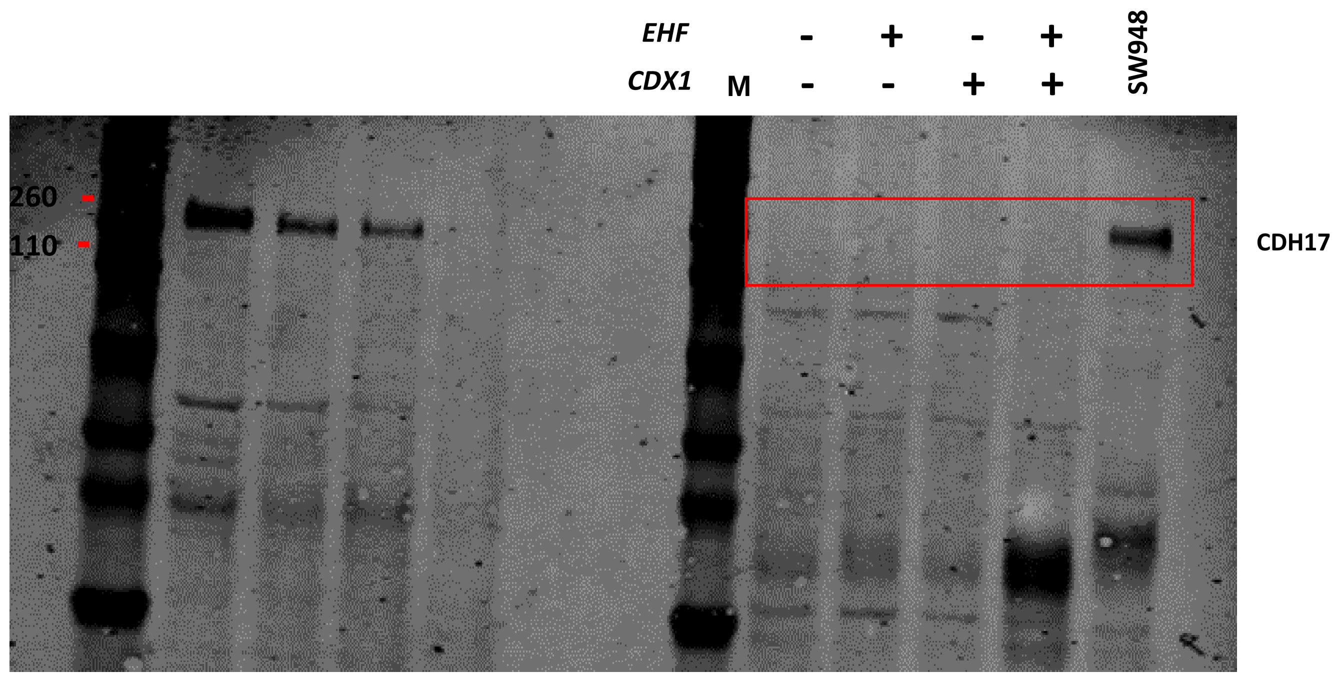

* This image shown represents the full western blot. Edges were not present due to the scanned image border being smaller than the western membrane.

Fig 2D

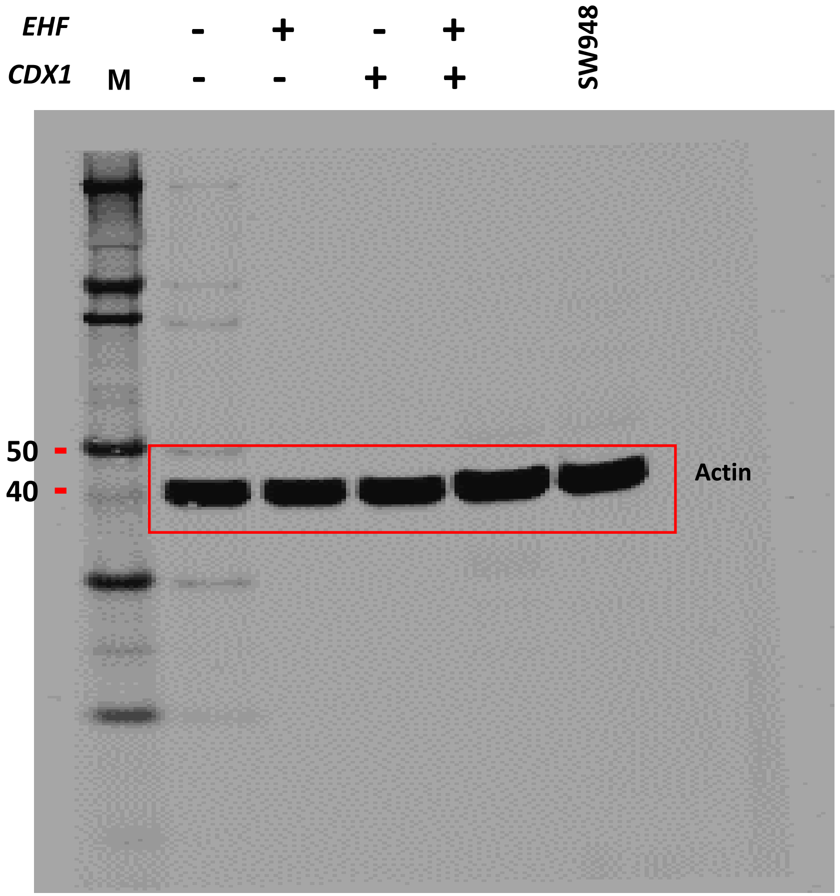

Fig 4F

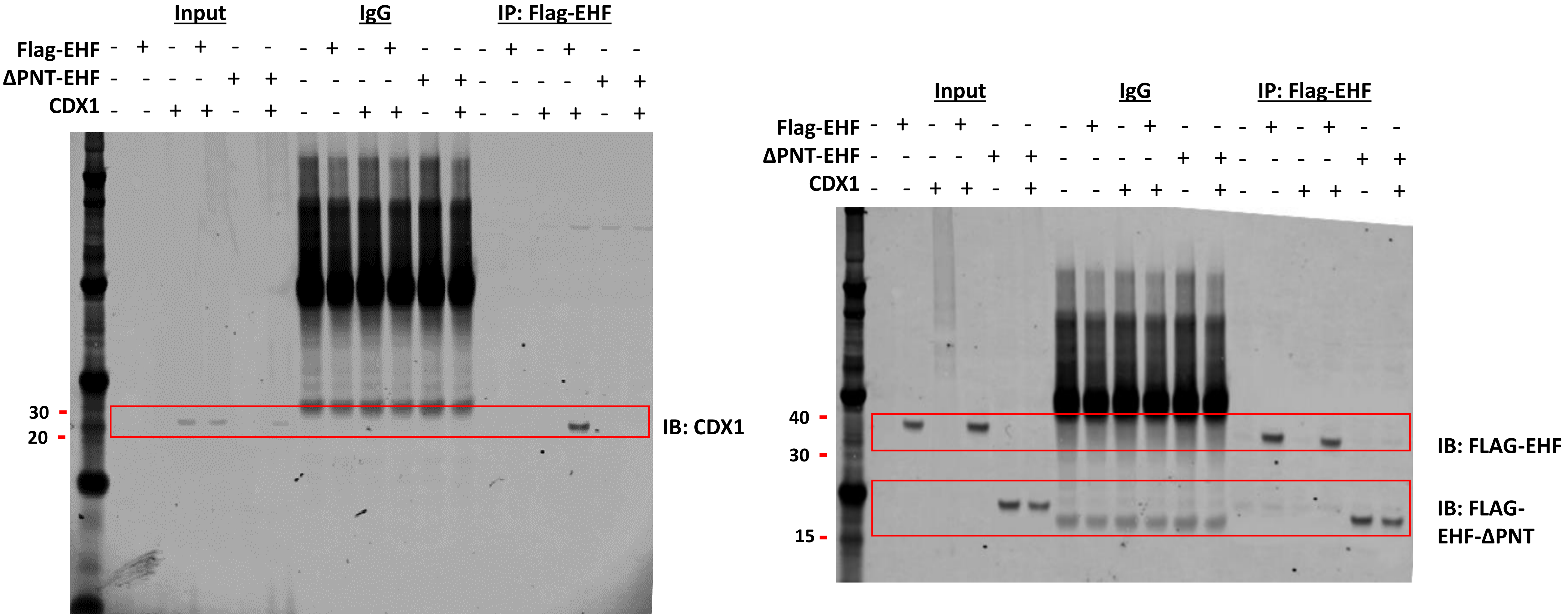

* This image shown represents the full western blot. Edges were not present due to the scanned image border being smaller than the western membrane.

Fig 4G

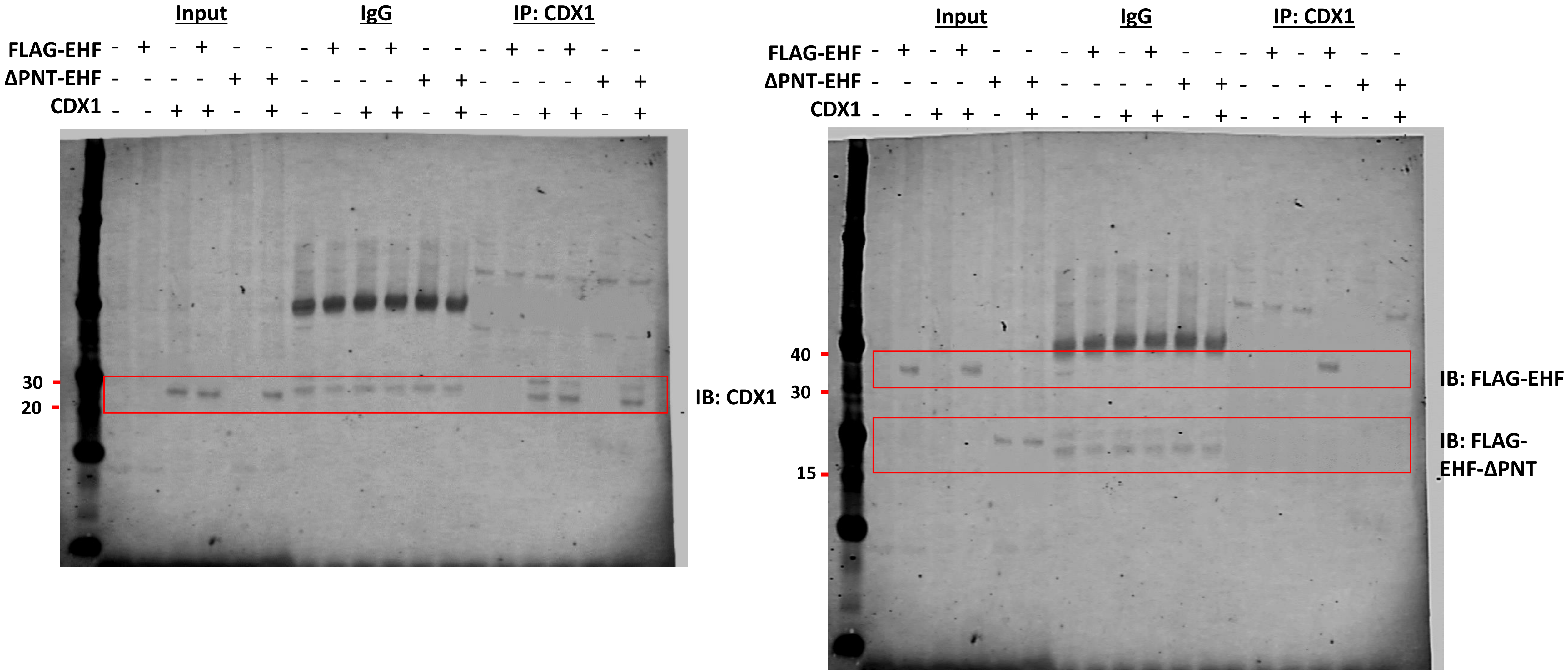
Input
IgG
IP: CDX1
FLAG-EHF
ΔPNT-EHF
CDX1
30
20
IB: CDX1
Input
IgG
IP: CDX1
FLAG-EHF
ΔPNT-EHF
CDX1
40
30
15
IB: FLAG-EHF
IB: FLAG-EHF-ΔPNT

Fig 4K

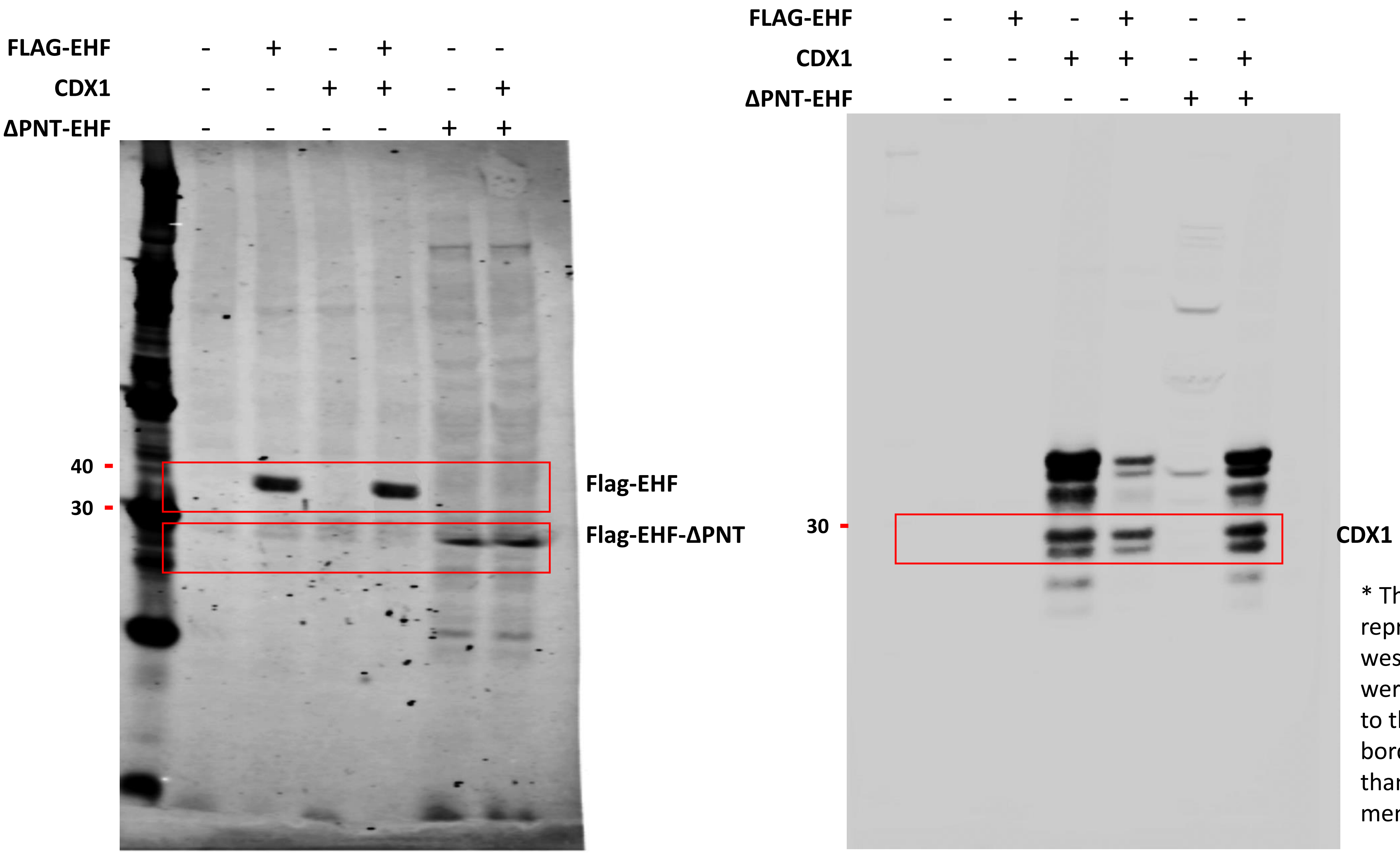

* This image shown represents the full western blot. Edges were not present due to the scanned image border being smaller than the western membrane.

Fig 4K

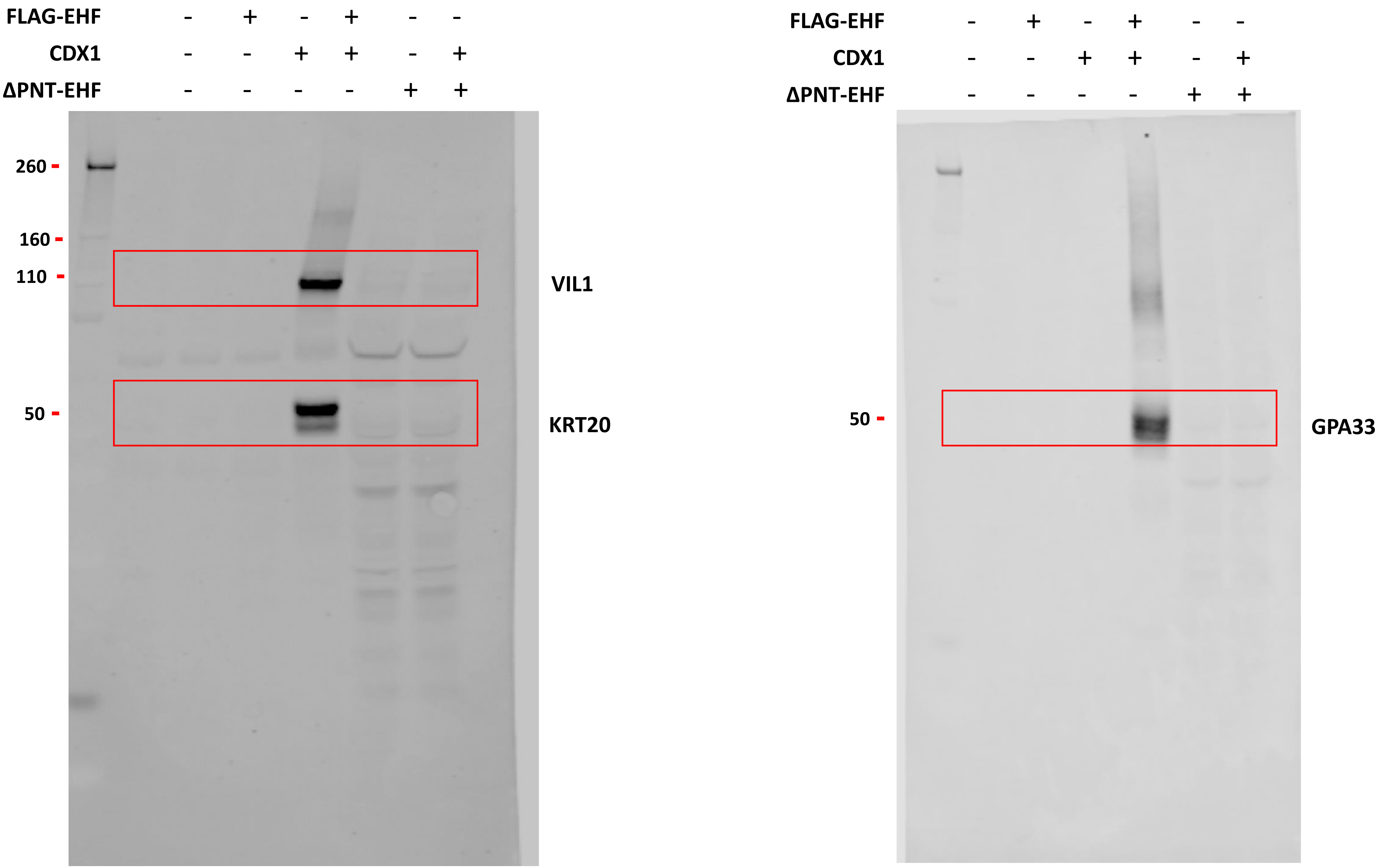
FLAG-EHF - + - + - -
CDX1 - - + + - +
ΔPNT-EHF - - - - + +
260
160
110
50
VIL1
KRT20
FLAG-EHF - + - + - -
CDX1 - - + + - +
ΔPNT-EHF - - - - + +
50
GPA33

Fig 4K

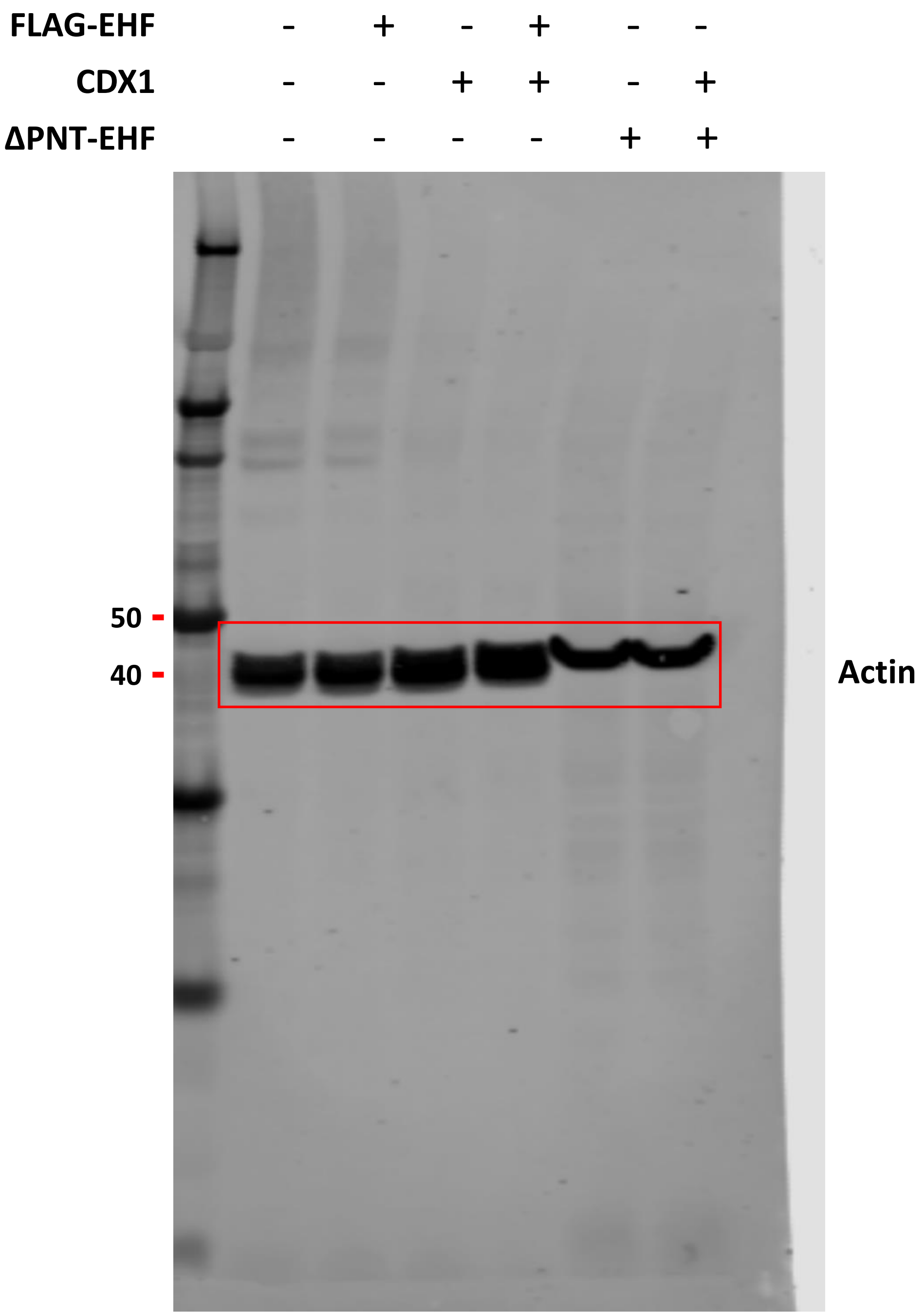

Fig S4B

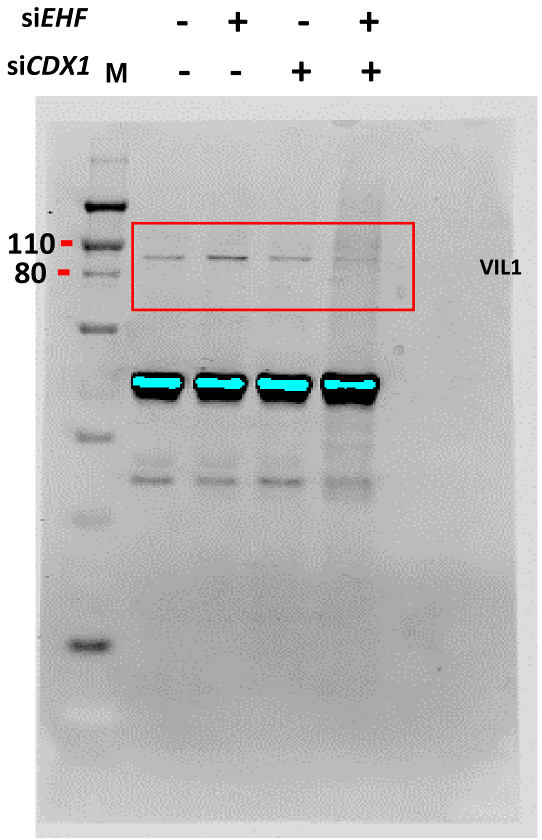

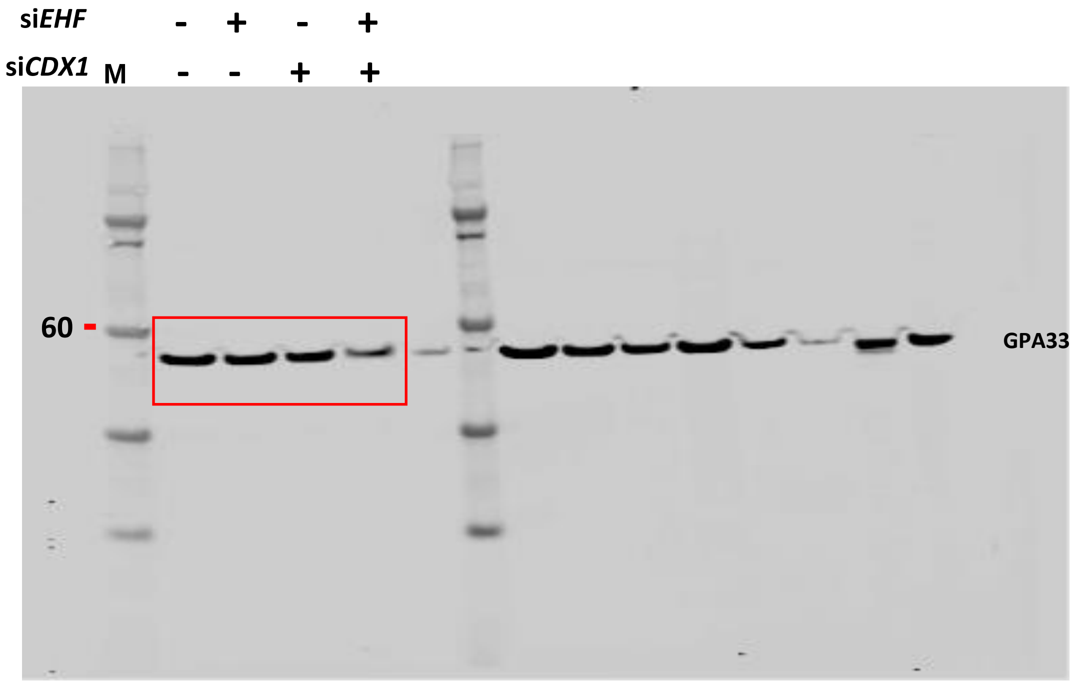

* This image shown represents the full western blot. Edges were not present due to the scanned image border being smaller than the western membrane.

Fig S4B

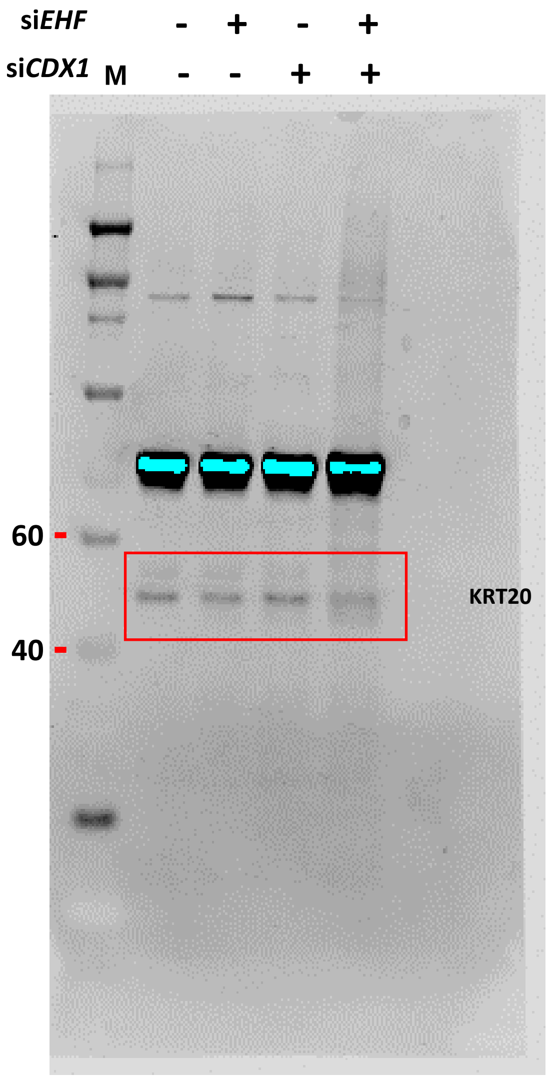

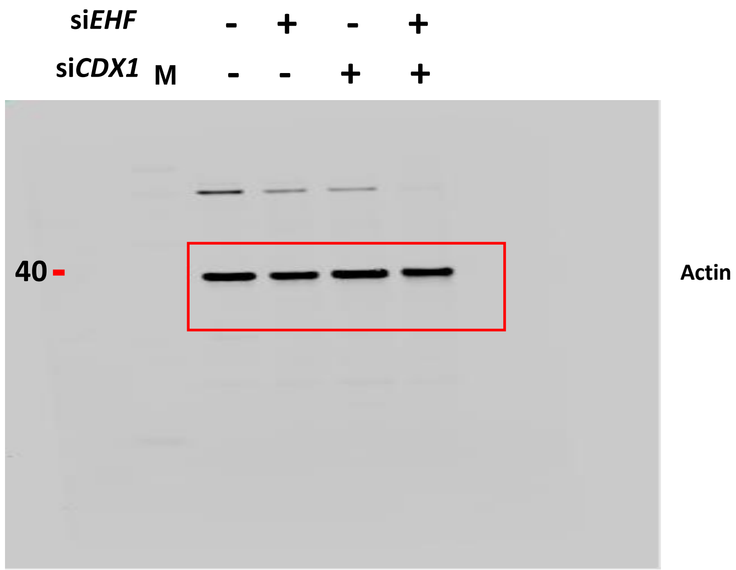

* This image shown represents the full western blot. Edges were not present due to the scanned image border being smaller than the western membrane.

Fig S4C

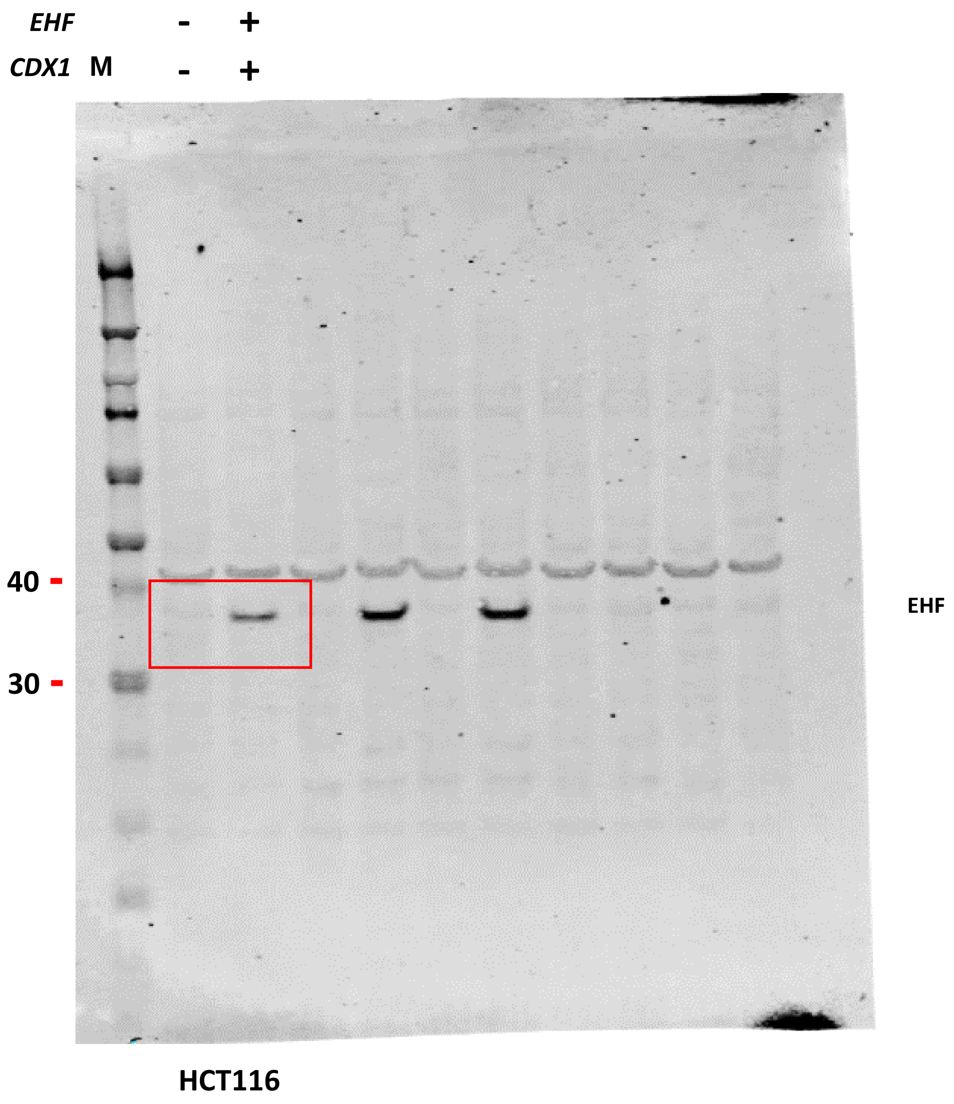

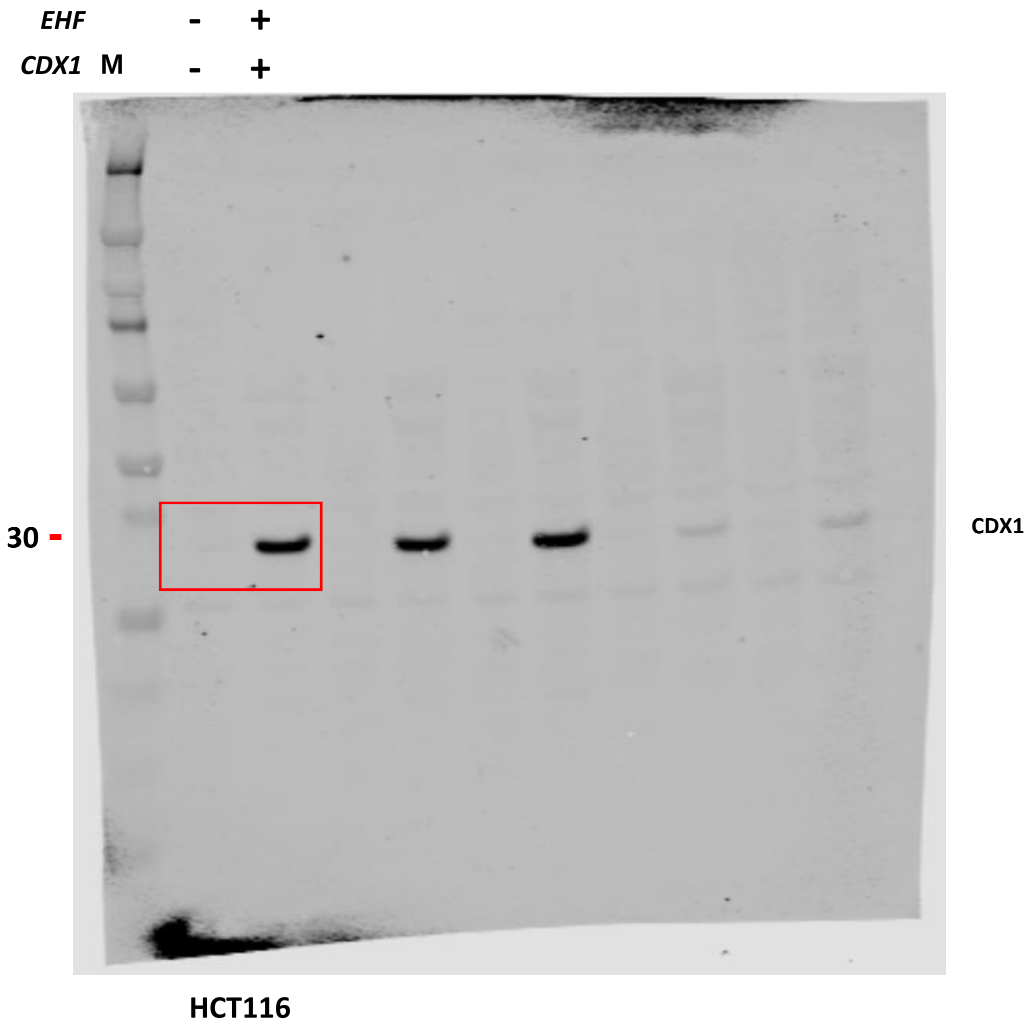

Fig S4C

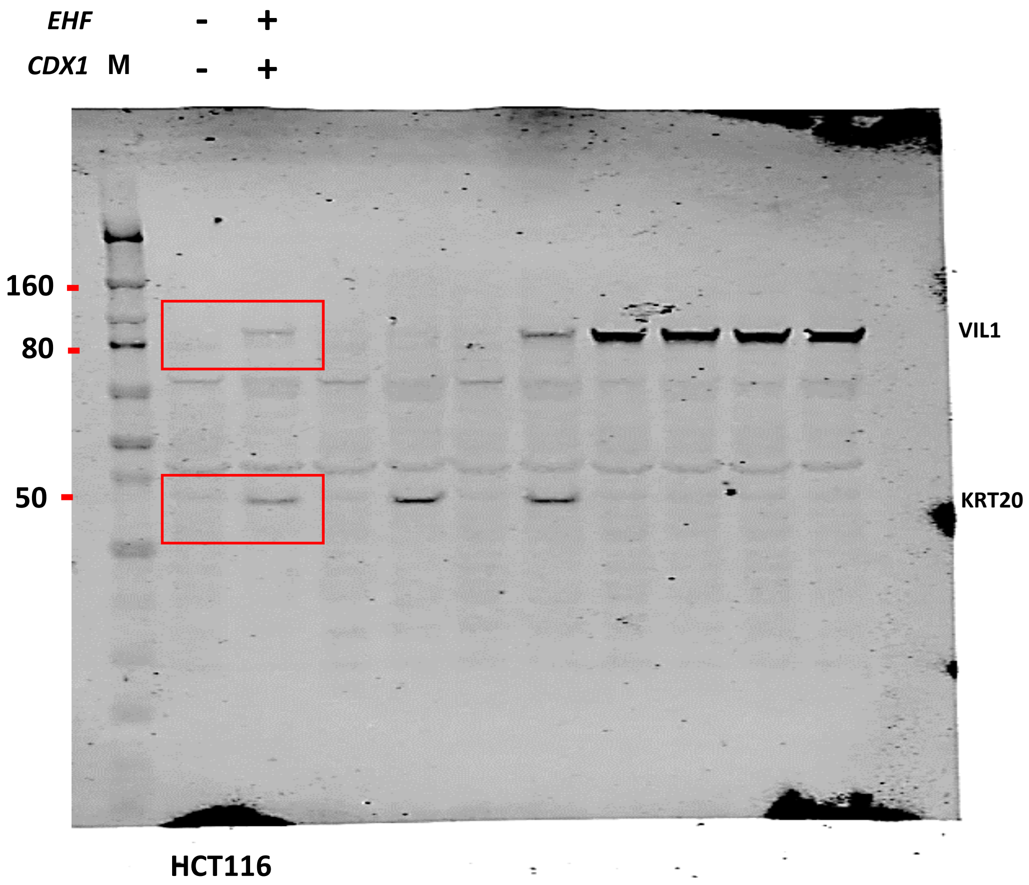
EHF - +
CDX1 M - +
160
80
50
VIL1
KRT20
HCT116

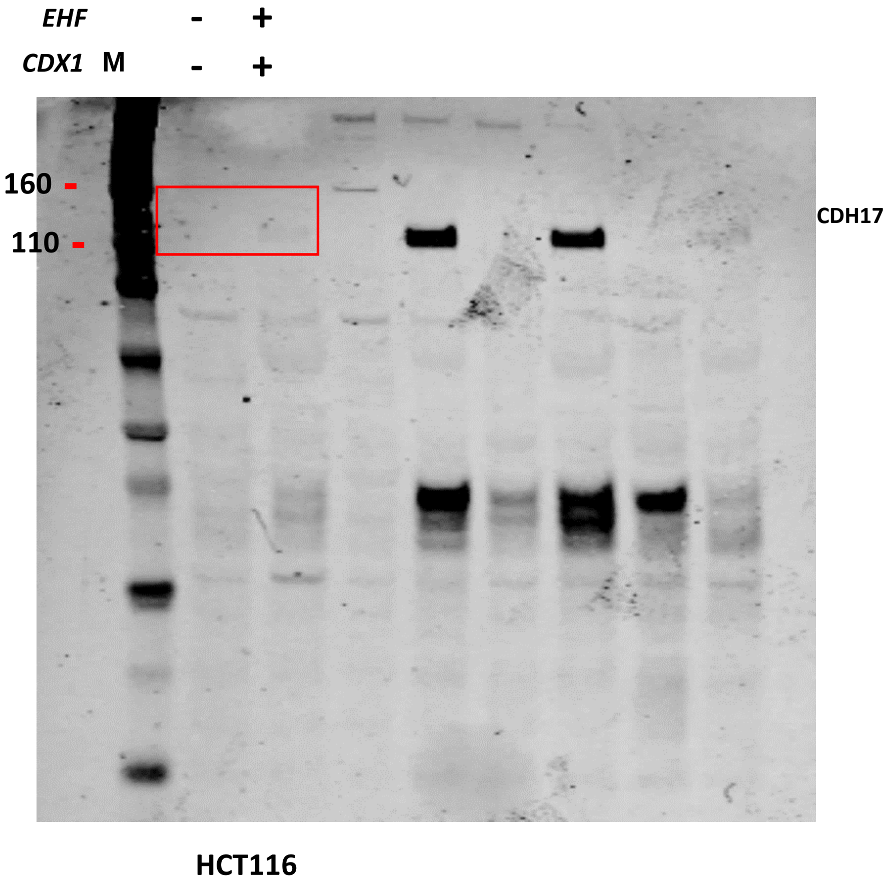
EHF - +
CDX1 M - +
160
110
CDH17
HCT116

Fig S4C

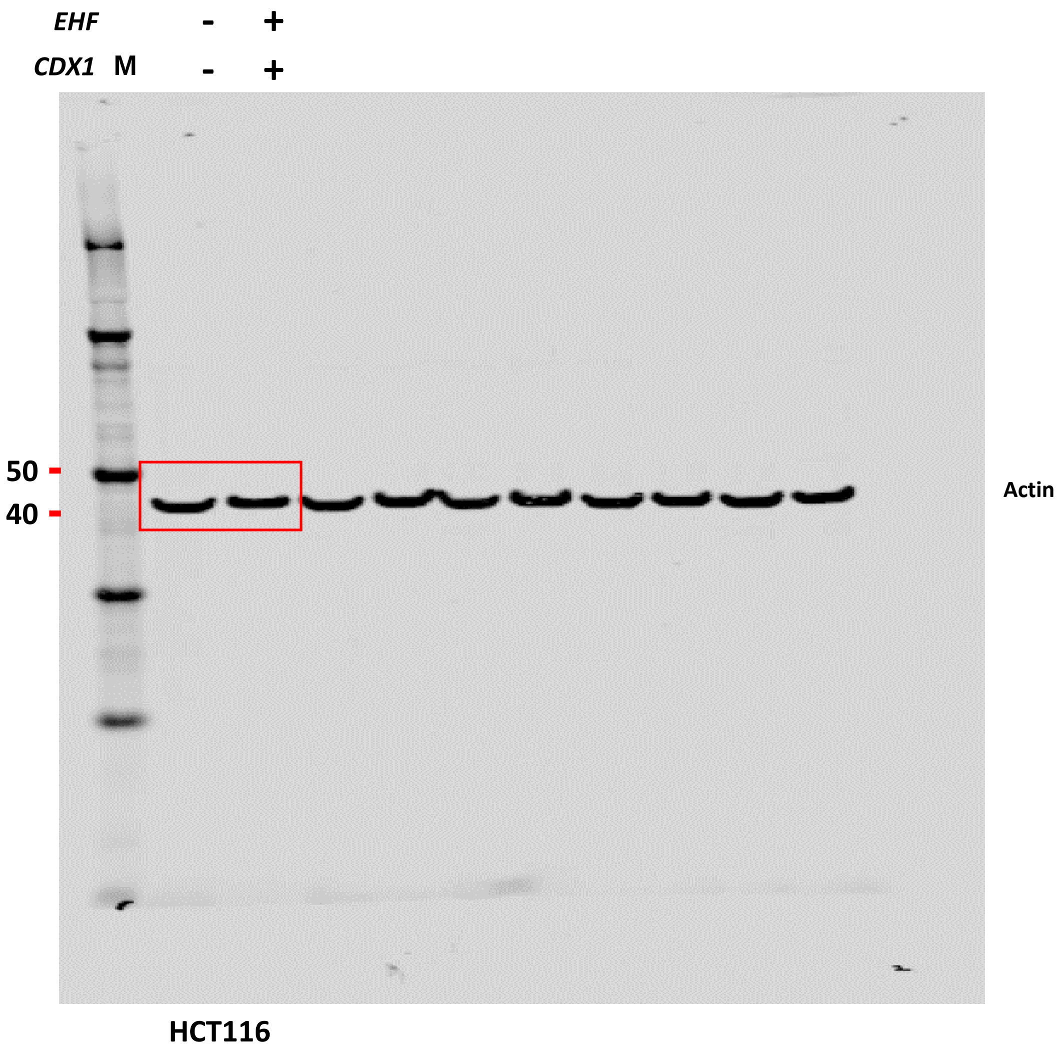

* This image shown represents the full western blot. Edges were not present due to the scanned image border being smaller than the western membrane.

Fig S4C

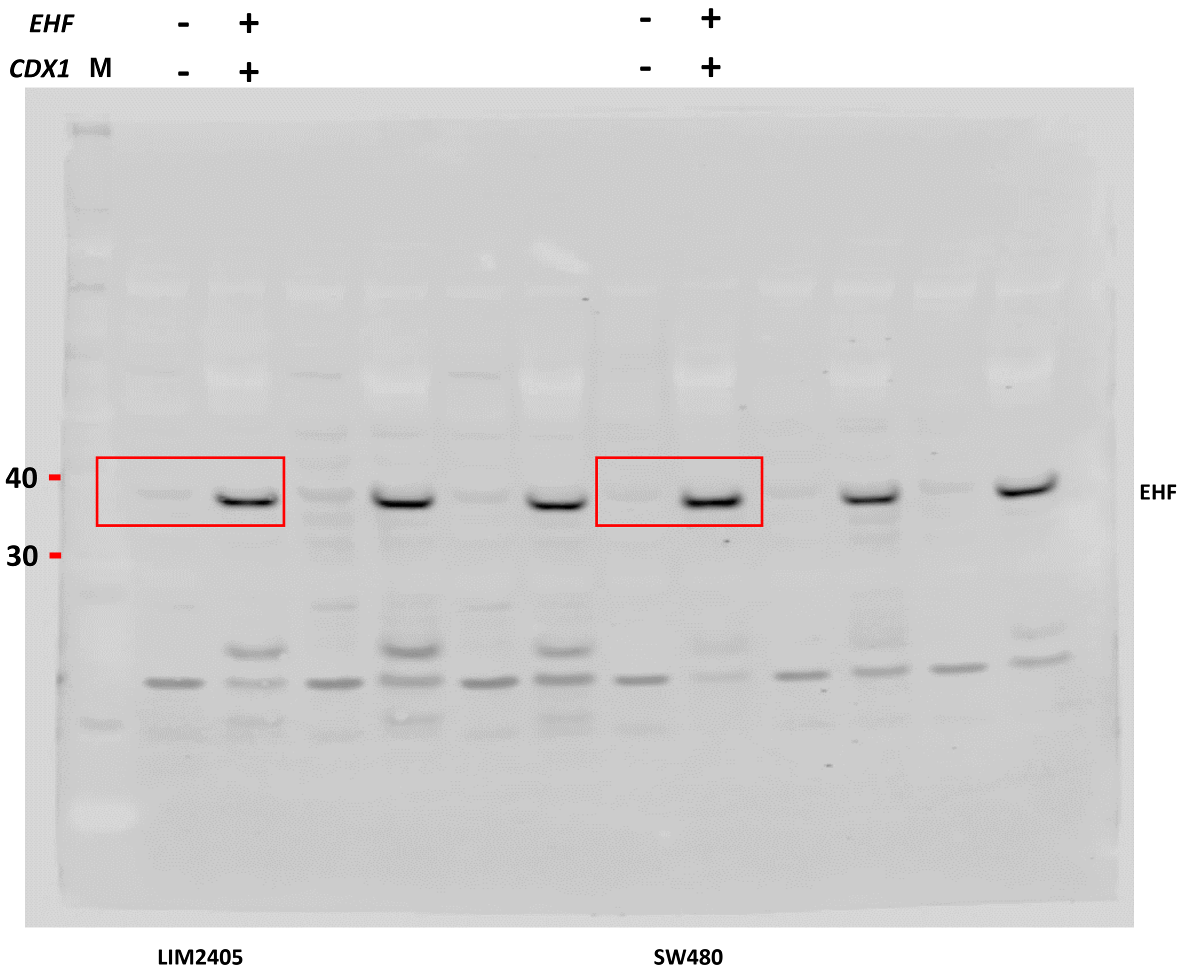

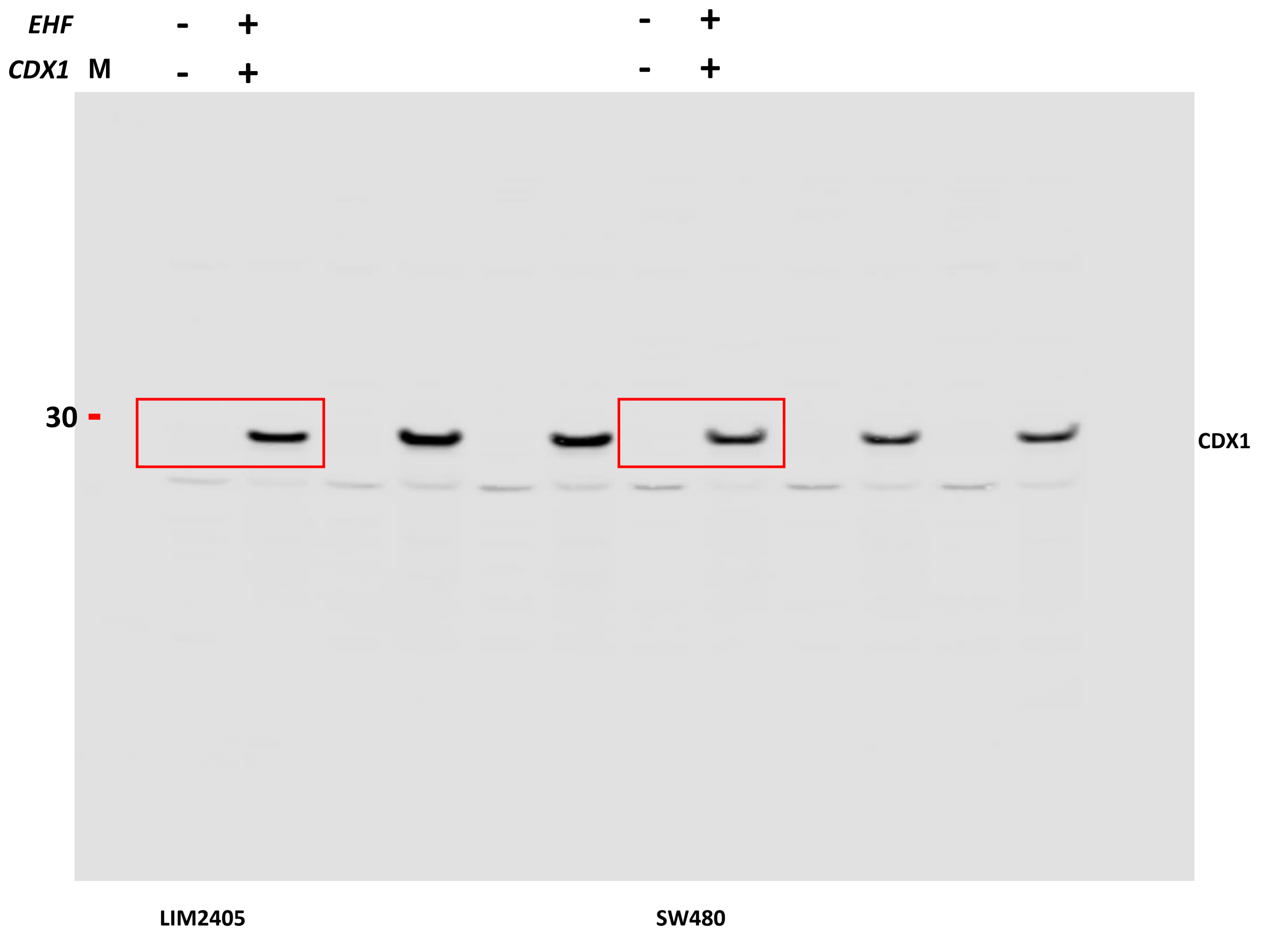

* This image shown represents the full western blot. Edges were not present due to the scanned image border being smaller than the western membrane.

Fig S4C

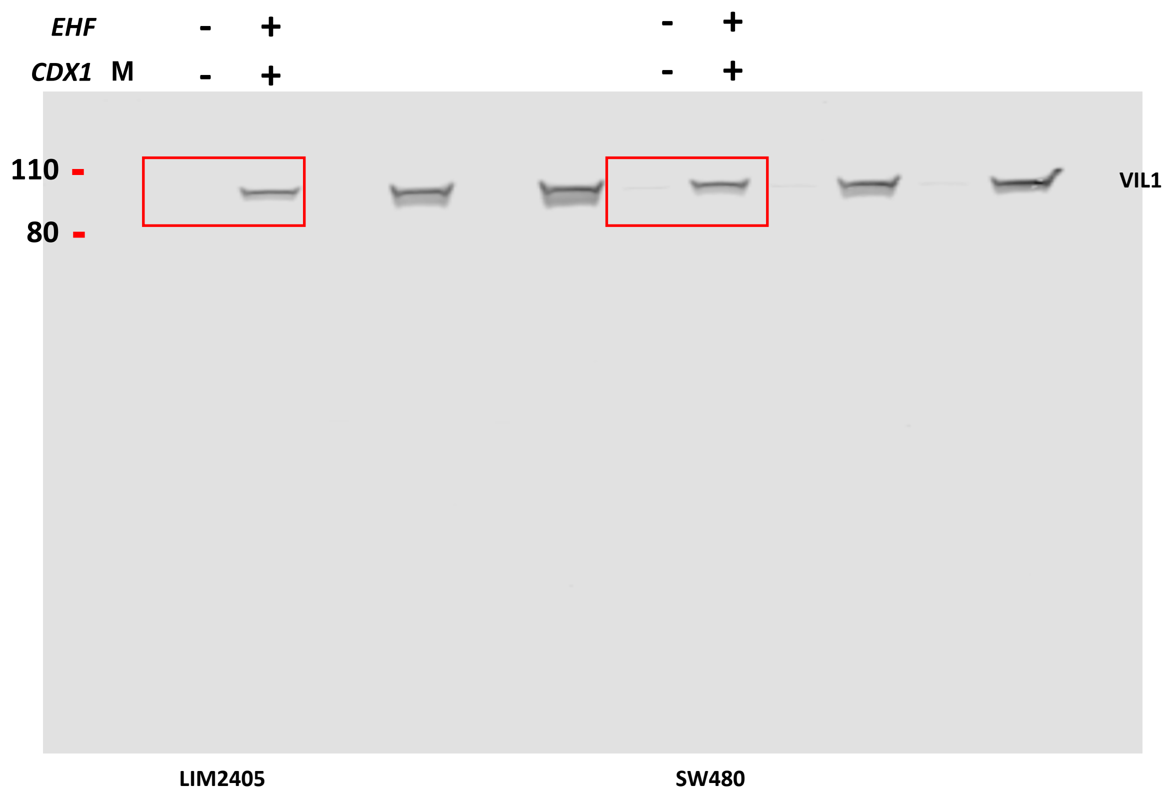

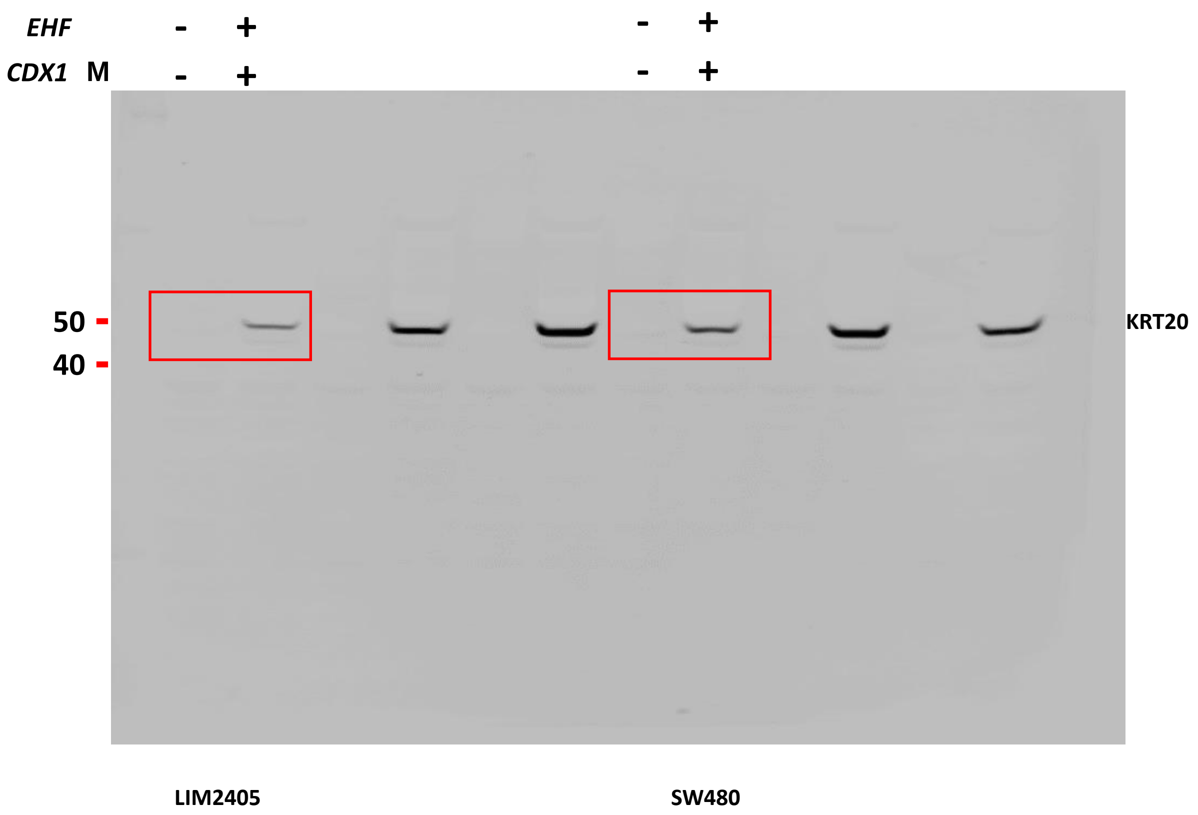

* This image shown represents the full western blot. Edges were not present due to the scanned image border being smaller than the western membrane.

Fig S4C

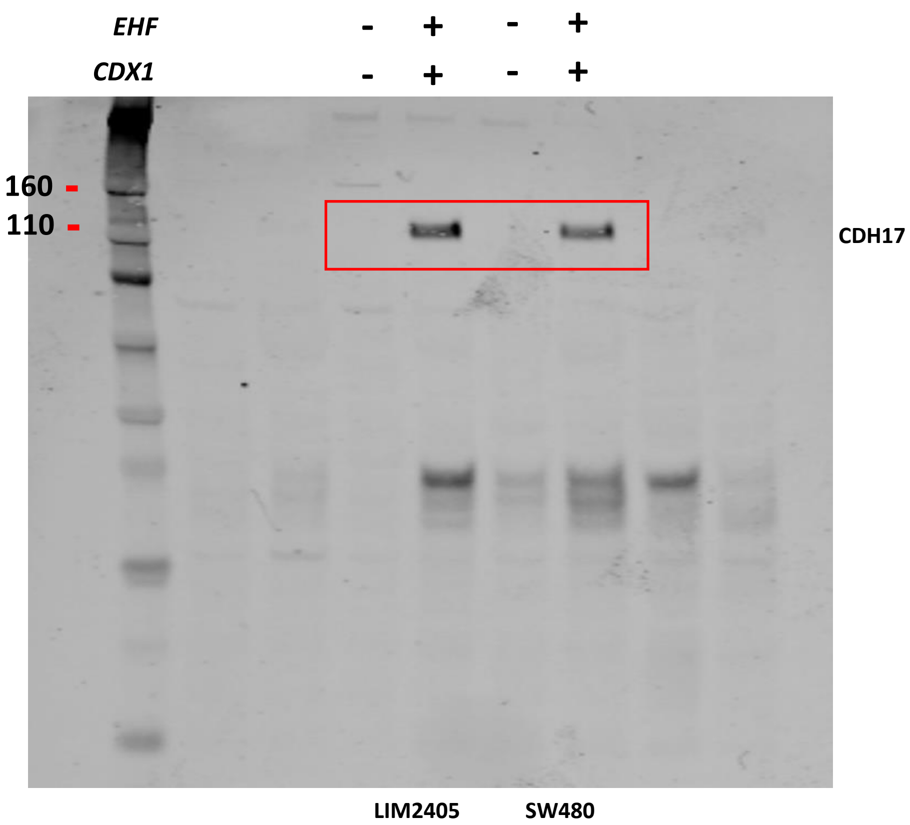

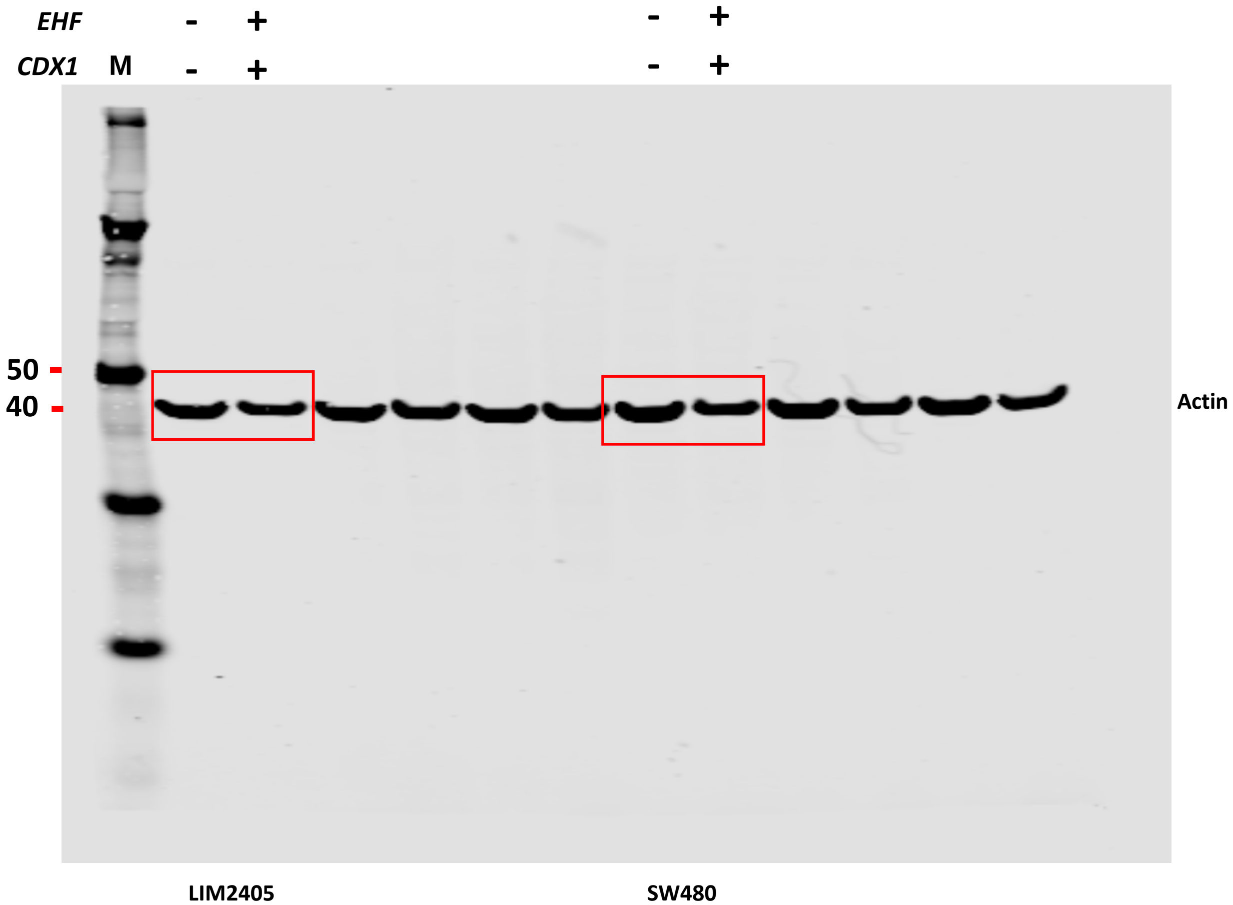

* This image shown represents the full western blot. Edges were not present due to the scanned image border being smaller than the western membrane.

Fig S4D

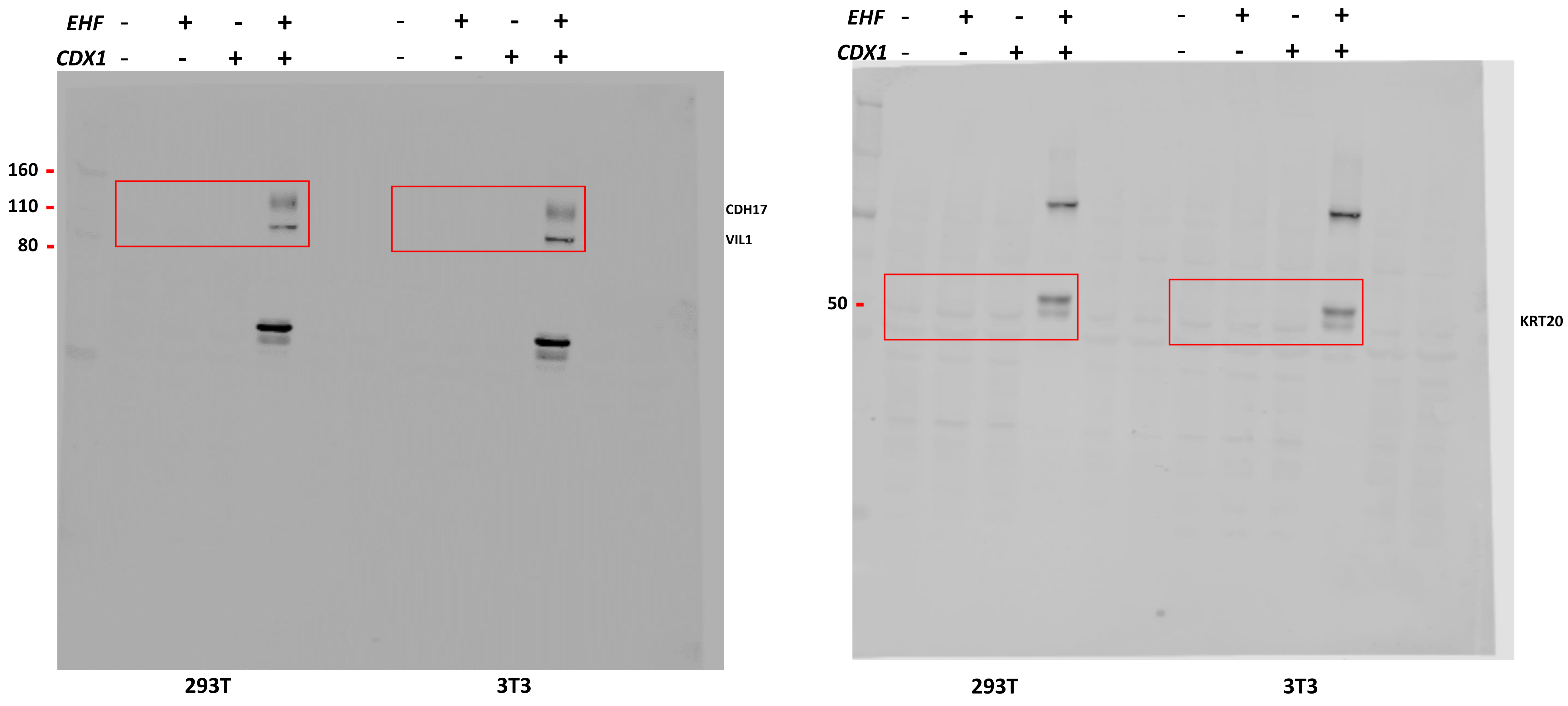
EHF - + - + - + - +
CDX1 - - + + - - + +
160
110
80
CDH17
VIL1
293T
3T3
EHF - + - + - + - +
CDX1 - - + + - - + +
50
KRT20
293T
3T3

Fig S4D

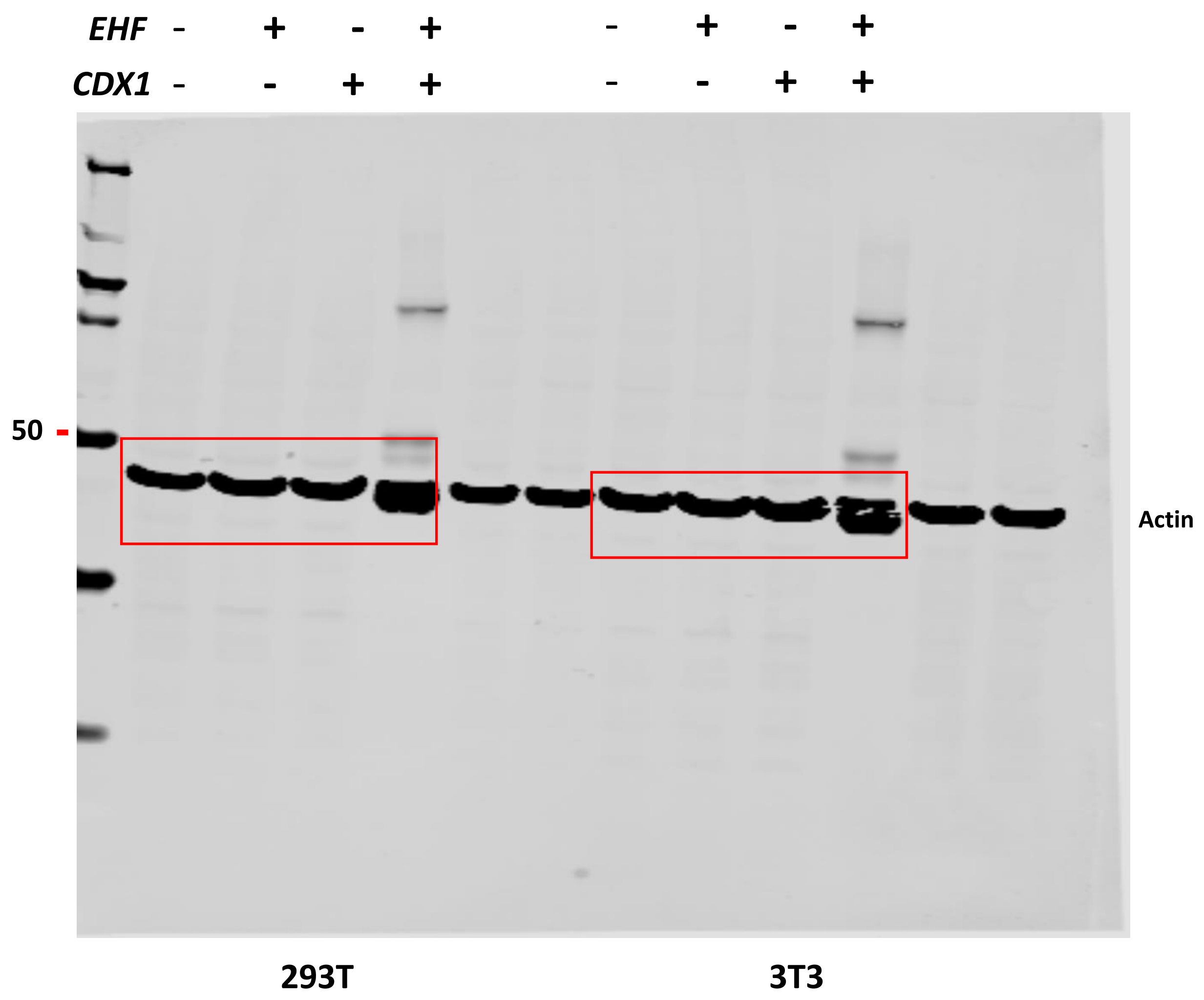

* This image shown represents the full western blot. Edges were not present due to the scanned image border being smaller than the western membrane.
